# CARMA: A platform for analyzing microarray datasets that incorporate replicate measures

**DOI:** 10.1186/1471-2105-7-149

**Published:** 2006-03-17

**Authors:** Kevin A Greer, Matthew R McReynolds, Heddwen L Brooks, James B Hoying

**Affiliations:** 1 Biomedical Engineering Program, Genomics Research Laboratory, University of Arizona, Tucson, Arizona, 85724, USA; 2 Department of Physiology, University of Arizona, College of Medicine, 1501 N Campbell Ave, Tucson, Arizona, 85724, USA

## Abstract

**Background:**

The incorporation of statistical models that account for experimental variability provides a necessary framework for the interpretation of microarray data. A robust experimental design coupled with an analysis of variance (ANOVA) incorporating a model that accounts for known sources of experimental variability can significantly improve the determination of differences in gene expression and estimations of their significance.

**Results:**

To realize the full benefits of performing analysis of variance on microarray data we have developed CARMA, a microarray analysis platform that reads data files generated by most microarray image processing software packages, performs ANOVA using a user-defined linear model, and produces easily interpretable graphical and numeric results. No pre-processing of the data is required and user-specified parameters control most aspects of the analysis including statistical significance criterion. The software also performs location and intensity dependent lowess normalization, automatic outlier detection and removal, and accommodates missing data.

**Conclusion:**

CARMA provides a clear quantitative and statistical characterization of each measured gene that can be used to assess marginally acceptable measures and improve confidence in the interpretation of microarray results. Overall, applying CARMA to microarray datasets incorporating repeated measures effectively reduces the number of gene incorrectly identified as differentially expressed and results in a more robust and reliable analysis.

## Background

High-density microarrays[1,2], in combination with high-throughput sequencing efforts[3,4], are proving invaluable in the investigation of complex systems. Researchers, however, struggle with the challenges associated with analyzing and interpreting the enormous amounts of data generated by microarray experiments. While initial analysis techniques focused on individual hybridizations and selected differentially expressed genes based on the ratio of the measured levels of gene expression between two samples[5], the results of any single microarray hybridization is subject to substantial variability[6], and consequently are unreliable. Therefore, as with any biological assay, robust microarray experiments rely on replication[7].

Kerr et al. [8] first described the use of analysis of variance (ANOVA) in combination with optimal experimental designs incorporating replicate measures, for microarrays. This technique uses a basic additive linear model to account for known sources of variability, thereby improving estimates of differences in gene expression and providing a measure of confidence for those estimates. Others have expanded these techniques to incorporate mixed models[9], multi-factorial designs[10], and biological replication strategies[11]. In addition, advancements in data transformation [12-14] and normalization[13,15,16] have improved the quality of the data to which subsequent analyses are applied.

In an effort to apply many of these powerful statistical techniques to our microarray datasets, we have developed an analysis platform with supporting software named CARMA (**C**omputational **A**nalysis of **R**eplicate **M**easures for **A**rrays). In addition to performing ANOVA on microarray datasets that incorporate replication, CARMA also performs all of the necessary steps for calculating differential expression in a microarray data set including importing, transforming, and normalizing the raw data files. The analysis is designed to be easy to apply, require only a basic understanding of the underlying principles, and provides output in both an easy to interpret graphical format and a delimited text file. Each step in the process was chosen for its broad applicability to microarray data, with user-defined parameters tailoring the analysis to each experiment. To demonstrate the utility of our approach, we present an example analysis of a microarray experiment designed to characterize gene expression in an aquaporin-1 knockout mouse model.

## Implementation

CARMA was implemented using the *R programming language and environment*[17] because of its "wide variety of statistical (linear and nonlinear modeling, classical statistical tests, time-series analysis, classification, clustering, etc) and graphical techniques". In addition R is available at no cost and runs on a variety of computing platforms. Under CARMA, analysis begins with data files and user-defined parameters being read from delimited files. Normalization between the two channels of each array and between both channels of all arrays is achieved using the *loess *function, which was chosen because of its ability to perform simultaneous location and intensity dependent lowess normalization. ANOVA is implemented using the *aov *function utilizing partitioned error for replicates, or the *lme *function for more complicated models, including mixed models. Graphical output and delimited text files are generated to present the results of the normalization, analysis of variance, and the ANOVA contrasts. CARMA is available as an R package for Microsoft Windows [Additional file 2], and as an R source file [Additional file 3]. A user manual is also available [Additional file 1].

## Results and discussion

### Example dataset

The microarray dataset used for this manuscript contains measurements of gene expression in kidneys from adult aquaporin-1 knockout and wild type mice[18] (GEO Accession GSE2402) [additional files 4, 5, 6, 7, 8]. In brief, RNA from kidney medullae of three aquaporin-1 knockout mice and three wild type mice was reverse-transcribed incorporating an amino modified dUTP, labeled using Alexa Fluor 546 and 647 ester dyes, and hybridized to a custom microarray containing the NIA Mouse 15 K cDNA clone set[19] with each clone printed in duplicate. Slides were scanned using an Applied Precision arrayWoRx Biochip Reader and image analysis was accomplished using softWoRx Tracker software. Because this experiment is balanced for both mouse (each mouse sample is hybridized twice with each dye) and genotype (3 mice of each genotype were used), it was possible to perform two different analyses; one to determine differences in gene expression between mice and the other to determine differences in gene expression between the aquaporin-1 knockout and wild type groups. The results of the comparison between the aquaporin-1 knockout and wild type groups have been reported previously [18], therefore this paper presents the comparison of gene expression between individual mice in order to demonstrate the functionality of CARMA.

### Experimental design

**Figure 1:**
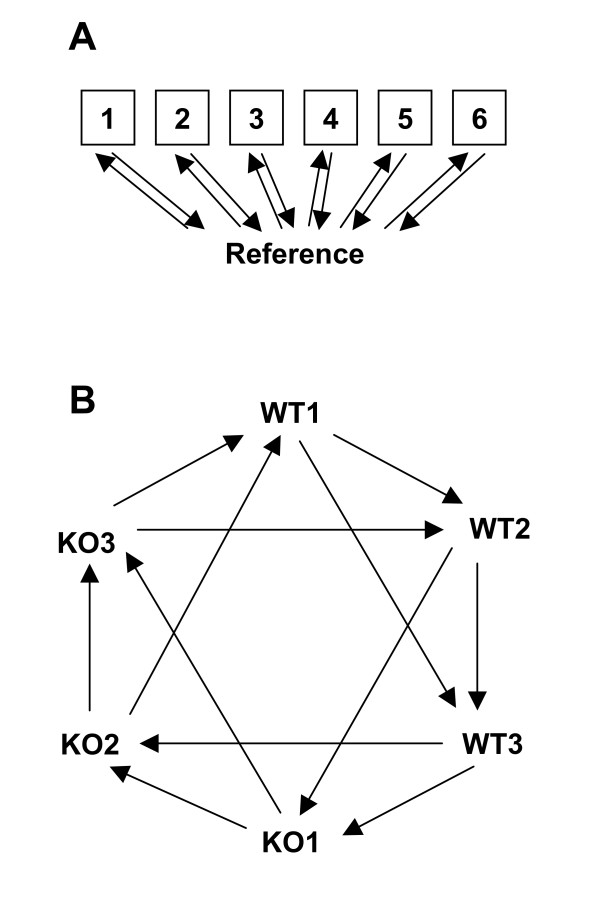
Hybridization scheme for 6 samples where all samples are hybridized against a reference sample. **B **Interwoven loop hybridization scheme for the aquaporin-1 knockout experiment used in this study, where W1-W3 and KO1-KO3 denote each of the wild type and aquaporin-1 deficient mice respectively. Each arrow in both schemes represents one hybridization, with the tail of the arrow denoting labeling with the Alexa 546 dye and the head of the arrow denoting labeling with the Alexa 647 dye.

Successful microarray data analysis depends on a sound experimental design. The most common approach consists of all samples of interest being hybridized against a common reference sample (with or without swapping the dyes) (Figure 1). While this approach is useful for experiments with large numbers of samples, or cases where all samples are not available at one time, it is usually possible to implement a more efficient design. In the "Interwoven Loop" design[20] employed in the aquaporin-1 knockout experiment (Figure 1), samples are hybridized against each other, thus providing twice as much data for the samples of interest than the reference design for the same number of hybridizations. Applying this balanced design, in which each variable of interest participates in the same number of hybridizations, enables the calculation of both experimental and biological effects.

### Analysis of variance (ANOVA) and linear model

In the simplest sense, microarray data is the measured intensities, at defined wavelengths, of elements (or "spots"), which have been arrayed on a glass slide. Included in these measurements are numerous sources of variability. Given that a two-channel microarray experiment consists of multiple samples labeled with two dyes hybridized to multiple arrays containing multiple spots (that represent genes), there are four main sources of variability termed: Array, Dye, Gene, and Variety[8]. The Array term refers to the variability in the measured intensities associated with a specific hybridization, due to either variability between slides, variability between hybridizations (e.g. differences in the amount of cDNA used in each hybridization), or both. The Dye term refers to variability caused by differences in fluorochrome chemistry, coefficient of extinction, incorporation efficiency, photobleachability, scanner sensitivity, etc. The Gene term refers to each element (or replicate elements) on the microarray. Since each element will hybridize to its complementary sequence in each sample, the intensity of each spot will depend on its nucleotide sequence, which is considered unique. The Variety term refers to the distinguishing feature of interest (such as time, treatment, or dosage) in the experimental samples. The goal of most microarray experiments is to determine the effect of this Variety term on the measured intensity for each element. In other words, how does dosage (or time, treatment, genotype, etc.) affect the expression of each gene?

Based on the four main effects (Array, Dye, Gene, Variety) and allowing for all interactions between those effects, there are 16 possible terms that could be included in the model[20], however many of the interaction effects do not make practical sense. In addition, performing a log-based transformation (see later sections) on the microarray dataset before applying the linear model allows the use of an additive linear model. Equation 1.1 describes the collection of mathematical equations that can be used to calculate values for each of the known factors that contribute to the transformed measured intensities:

*i*_*ijkl *_= *μ + A*_*i *_*+ D*_*j *_*+ G*_*k *_*+ (AD)*_*ij *_*+ (GA)*_*ki *_*+ (GD)*_*kj *_*+ (GV)*_*kl*_*+ ε *_*ijkl *_   (1.1)

Equation 1.1 defines a relatively complete model for the sources of variability in a microarray experiment; however there are practical limitations to its implementation. Utilizing a least squares approach to solve equation 1.1, even for reasonably small microarray datasets (e.g. 4 hybridization of a 10,000 element array), requires gigabytes of memory, precluding the use of a personal computer. In addition, it assumes equal variance between all genes. Splitting equation 1.1 into two equations, one containing all Gene independent terms, and another equation containing all gene-dependent terms, yields equations 1.2 and 1.3 respectively:

*i*_*ij *_*= μ +A*_*i *_*+ D*_*j *_*+AD*_*ij *_*+ ε *_*ij *_   (1.2)

*i*_*ijkl *_*= G*_*k *_*+ GA*_*ki *_*+ (GD)*_*kj *_*+ (GV)*_*kl*_*+ ε *_*ijkl *_   (1.3)

Applying these two models sequentially[9] to a dataset significantly reduces the memory requirements and time required for computation, and allows for gene specific variances. When using these two models, equation 1.2 effectively serves to perform a linear global normalization between the two channels for each array and between arrays. Instead, because only the Gene × Variety effect is usually of interest, and in order to allow for non-linear global normalization, CARMA performs a lowess normalization between the two channels of each array and between both channels of all arrays, followed by a gene-by-gene ANOVA implementing equation 1.3 alone. Limiting equation 1.3 to the subset of data for each gene yields equation 1.4:

iijkl=Gk+GAki+GDkj+GVkl+εijkl⇒iijl=μ+Ai+Dj+Vl+εijl     (1.4)
 MathType@MTEF@5@5@+=feaafiart1ev1aaatCvAUfKttLearuWrP9MDH5MBPbIqV92AaeXatLxBI9gBaebbnrfifHhDYfgasaacH8akY=wiFfYdH8Gipec8Eeeu0xXdbba9frFj0=OqFfea0dXdd9vqai=hGuQ8kuc9pgc9s8qqaq=dirpe0xb9q8qiLsFr0=vr0=vr0dc8meaabaqaciaacaGaaeqabaqabeGadaaakeaacqWGPbqAdaWgaaWcbaGaemyAaKMaemOAaOMaem4AaSMaemiBaWgabeaakiabg2da9iqbdEeahzaawaWaaSbaaSqaaiabdUgaRbqabaGccqGHRaWkcqWGhbWrcuWGbbqqgaGfamaaBaaaleaacqWGRbWAcqWGPbqAaeqaaOGaey4kaSIaem4raCKafmiraqKbaybadaWgaaWcbaGaem4AaSMaemOAaOgabeaakiabgUcaRiabdEeahjqbdAfawzaawaWaaSbaaSqaaiabdUgaRjabdYgaSbqabaGccqGHRaWkiiGacqWF1oqzdaWgaaWcbaGaemyAaKMaemOAaOMaem4AaSMaemiBaWgabeaakiabgkDiElabdMgaPnaaBaaaleaacqWGPbqAcqWGQbGAcqWGSbaBaeqaaOGaeyypa0Jae8hVd0Maey4kaSIaemyqae0aaSbaaSqaaiabdMgaPbqabaGccqGHRaWkcqWGebardaWgaaWcbaGaemOAaOgabeaakiabgUcaRiabdAfawnaaBaaaleaacqWGSbaBaeqaaOGaey4kaSIae8xTdu2aaSbaaSqaaiabdMgaPjabdQgaQjabdYgaSbqabaGccaWLjaGaaCzcamaabmaabaGaeGymaeJaeiOla4IaeGinaqdacaGLOaGaayzkaaaaaa@7407@    

Implementing this form of the equation reduces computation times to a few hours on a basic personal computer (500 MHz Pentium III with 512 MB of memory) for even relatively large data sets (e.g. an experiment involving 32,000 elements and 12 hybridization).

### Background subtraction and transformation

Most microarray scanners generate 16 bit numbers, resulting in measurements ranging from 0 to 65,535 for each pixel in the generated images. Microarray image processing software is then used to analyze each image, generating a multitude of measures for each spot, which are usually further processed to produce one measure of intensity for each channel for each spot. The most controversial part of this process is whether to subtract some measure of background (defined as the pixels around the areas delineated as spots) from each spot (defined as the pixels within each area delineated as a spot). Background subtraction is often used to reduce the negative impact of local image artifacts, and provide better estimates of gene expression by subtracting fluorescent signal that is unrelated to the fluorescently labeled hybridized target cDNA. Nevertheless, some researchers have concluded that background subtraction increases variance and degrades the performance of subsequent analyses[21]. In most cases, however, this increase in variance is not simply due to background subtraction, but the combination of background subtraction and a log-based transformation of the data. In effect, subtracting the background intensities from the spot intensities reduces the values for low intensity measurements to the point that error is no longer multiplicative, but additive, causing a log-based transformation to inflate the variance of these small values. In other words, the measurement error associated with small values is not proportional to the true value, but is a combination of some random error added to the true value. For instance, a log base 2 transformation assigns the same significance to the difference between 2 and 32 (log_2_(32) - log_2_(2) = 4) as the difference between 2000 and 32000 (log_2_(32000) - log_2_(2000) = 4), even though a difference of 32 is well within the noise of current microarray scanning technology and of no real significance.

The logarithm base 2 of the expression ratio is the most widely used transformation for microarray data due to its continuous range of values and similar treatment of up- and down-regulation[22]. It has the added effects of improving linearity and variance homogeneity, and converting multiplicative error into additive error, thus allowing the application of a linear additive model[8]. While log transformations work well for moderate to high signal intensities, they have the undesirable effect of magnifying small differences in intensity at low signal intensities[23]. In addition, log transformations cannot be applied to negative numbers, which can result from background subtraction, and they transform numbers between 0 and 1 into negative values. To address these problems some researchers have proposed discarding data below a threshold[24], while others have proposed variance-stabilizing transformations [12-14]. CARMA implements a version of the linlog transformation[13] that has been adapted to better cope with large negative numbers (where the local background signal intensity is significantly higher than the spot signal intensity) as follows.

Zik={log⁡2(−1/Yik)+2*log⁡2(di)−2/ln⁡(2)Yik≤−dilog⁡2(di)+Yik/(di*ln⁡(2))−1/ln⁡(2)−di<Yik<dilog⁡2(Yik)Yik≥di
 MathType@MTEF@5@5@+=feaafiart1ev1aaatCvAUfKttLearuWrP9MDH5MBPbIqV92AaeXatLxBI9gBaebbnrfifHhDYfgasaacH8akY=wiFfYdH8Gipec8Eeeu0xXdbba9frFj0=OqFfea0dXdd9vqai=hGuQ8kuc9pgc9s8qqaq=dirpe0xb9q8qiLsFr0=vr0=vr0dc8meaabaqaciaacaGaaeqabaqabeGadaaakeaacqWGAbGwdaWgaaWcbaGaemyAaKMaem4AaSgabeaakiabg2da9maaceaabaqbaeaabmGaaaqaaiGbcYgaSjabc+gaVjabcEgaNnaaBaaaleaacqaIYaGmaeqaaOGaeiikaGIaeyOeI0IaeGymaeJaei4la8IaemywaK1aaSbaaSqaaiabdMgaPjabdUgaRbqabaGccqGGPaqkcqGHRaWkcqaIYaGmcqGGQaGkcyGGSbaBcqGGVbWBcqGGNbWzdaWgaaWcbaGaeGOmaidabeaakiabcIcaOiabdsgaKnaaBaaaleaacqWGPbqAaeqaaOGaeiykaKIaeyOeI0IaeGOmaiJaei4la8IagiiBaWMaeiOBa4MaeiikaGIaeGOmaiJaeiykaKIaaCjaVdqaaiabdMfaznaaBaaaleaacqWGPbqAcqWGRbWAaeqaaOGaeyizImQaeyOeI0Iaemizaq2aaSbaaSqaaiabdMgaPbqabaaakeaacyGGSbaBcqGGVbWBcqGGNbWzdaWgaaWcbaGaeGOmaidabeaakiabcIcaOiabdsgaKnaaBaaaleaacqWGPbqAaeqaaOGaeiykaKIaey4kaSIaemywaK1aaSbaaSqaaiabdMgaPjabdUgaRbqabaGccqGGVaWlcqGGOaakcqWGKbazdaWgaaWcbaGaemyAaKgabeaakiabcQcaQiGbcYgaSjabc6gaUjabcIcaOiabikdaYiabcMcaPiabcMcaPiabgkHiTiabigdaXiabc+caViGbcYgaSjabc6gaUjabcIcaOiabikdaYiabcMcaPaqaaiabgkHiTiabdsgaKnaaBaaaleaacqWGPbqAaeqaaOGaeyipaWJaemywaK1aaSbaaSqaaiabdMgaPjabdUgaRbqabaGccqGH8aapcqWGKbazdaWgaaWcbaGaemyAaKgabeaaaOqaaiGbcYgaSjabc+gaVjabcEgaNnaaBaaaleaacqaIYaGmaeqaaOGaeiikaGIaemywaK1aaSbaaSqaaiabdMgaPjabdUgaRbqabaGccqGGPaqkaeaacqWGzbqwdaWgaaWcbaGaemyAaKMaem4AaSgabeaakiabgwMiZkabdsgaKnaaBaaaleaacqWGPbqAaeqaaaaaaOGaay5Eaaaaaa@A48F@

where Z_ik _represents the transformed intensities, Y_ik _represents the untransformed intensities, and d_i _represents the threshold between the log and linear portions of the transformation. The subscripts i and k denote the array and element (spot) for each intensity, respectively. This modified linlog transformation is symmetrical around 0 (raw signal) and both it and its first derivative are continuous. It is similar to a log_2 _transformation for both large positive and large negative intensities, and a linear transformation at low intensities (positive or negative). For the example analysis in this manuscript, the crossover point (d_*i*_) between the linear and log portions of the transformation was calculated based on the median of one standard deviation of the local background of all spots. This method of calculating the cutoff for the linlog function was based on the observation that the variability in the measured intensities at background levels provides a good indication of the minimum intensity that the scanner can accurately measure (the point at which error becomes multiplicative). This approach has worked well in practice, and has the advantage of not assuming any distribution for the data. Recently CARMA has been enhanced to include the capability of calculating the linlog crossover point based on minimizing the absolute deviation of the inner quartile range (IQR) for each bin from the median IQR, for 20 bins spanning the range of intensities for each hybridization.

**Figure 2:**
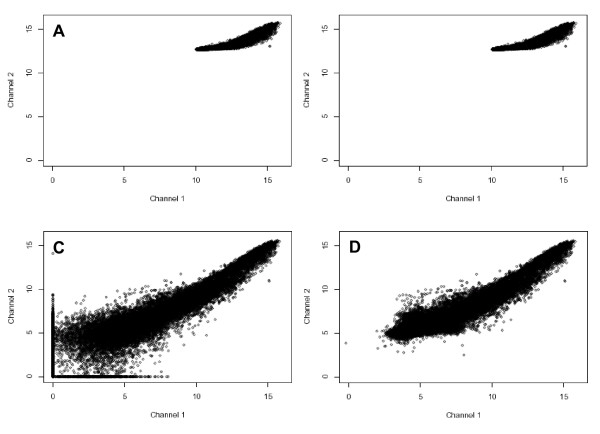
Plot of the pre-normalized data, channel one (green) vs. channel 2 (red), for one hybridization in the aquaporin-1 dataset after applying **A **log2 transformation without background subtraction or **B **linlog transformation without background subtraction or **C **log2 transformation after background subtraction or **D **linlog transformation after background subtraction.

Utilizing the aquaporin-1 dataset, we compared the effect of local background versus no background subtraction, and the consequences of applying either a log_2 _or linlog transformation, on the ability to detect differentially expressed genes. Because this dataset consists of four replicate measurements for 6 mice, one success criterion is minimizing the within mouse variance while maximizing the between mouse variance[25], which equates to maximizing the ANOVA *F *value for the Variety (mouse) term, in equation 1.4. In the case of log_2 _background subtracted data, values less than 1 were set to 1, and in the case of the linlog transformation, the crossover point between the linear and logarithmic segments was set to the median of one standard deviation of the background for all spots. Figure 2 illustrates the effect of each background subtraction/transformation method on one of the hybridizations in the dataset. The most obvious change due to background subtraction is the expansion of the range of the data resulting from the removal of the relatively large signal floor associated with the longer exposure times and CCD-based image capture employed in the Applied Precision arrayWoRx Biochip Reader. This large signal floor also negates any difference between the log_2 _and linlog transformation on the non-background subtracted data because error remains multiplicative across the entire range of values. The difference in transformation applied to the background subtracted data however, is obvious as indicated by the larger spread of the lower range of the log_2 _transformed data as compared to the linlog transformed data. Table 1 presents a summary quantitative comparison of the effect of each transformation on the ratio of the between group mean squares over the within group mean squares after location and intensity dependent lowess normalization and gene-by-gene analysis of variance. On a row-by-row basis after ranking, the ANOVA *F *values for the linlog transformed local background subtracted data were larger than any of the other combinations of background subtraction and transformation, for every element in the dataset. Also, applying a step-up *p*-value procedure[26] to the ANOVA *p*-value controlling at a 5% false discovery rate resulted in 8 genes being identified as differentially expressed for both of the non-background subtracted datasets, and 22 and 129 genes being identified as differentially expressed for the log_2 _transformed and linlog transformed background subtracted data, respectively.

### Normalization

Differences in fluorochrome characteristics, scanner wavelength sensitivities and settings, dye incorporation, and other non-biological effects all contribute to differences in the intensities between the two channels of any hybridization. And while global normalization techniques apply the same adjustment to every spot for a hybridization[22], it is often necessary to correct for intensity and location specific effects[13,15,27-29]. CARMA implements a locally weighted regression (lowess)[15,16] transformation that can adjust for either intensity or location (or both) dependent effects. In addition, CARMA normalizes between both channels of all arrays, serving to minimize the Array term in the linear model and aiding in the visualization of the normalized data.

**Figure 3:**
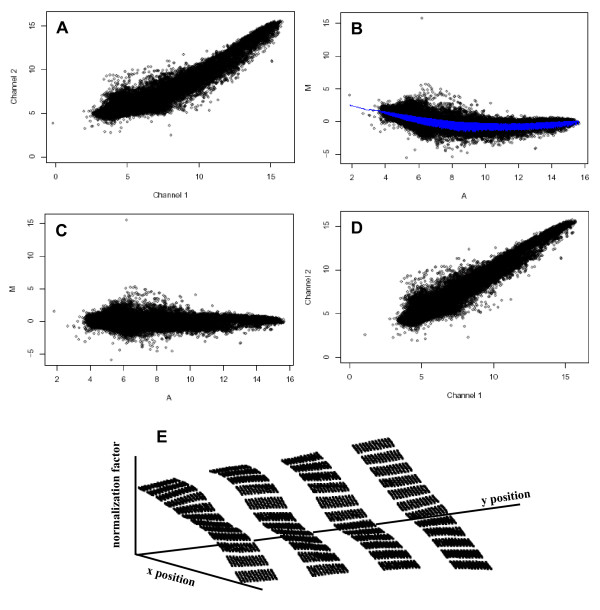
Normalization of a two-channel hybridization. **A **The linlog transformed data before normalization plotted as channel 1 (Alexa 546 dye) versus channel 2 (Alexa 647 dye) intensities. **B**,**C **Ratio-Intensity plot before (**B**) and after (**C**) spatial and intensity lowess normalization. "M" refers to the log ratio of the two channels and "A" refers to the geometric mean of the spot intensity for both channels. The blue line in **B **is the spatial/intensity lowess normalization fitted curve, with the curvature of the line representing the intensity-dependent fit and the width of the line representing the spatial component of the lowess normalization. **D **The final normalized data used for the ANOVA. **E **A plot of the correction factors, by array position, used to normalize intensities due to spatial effects only. Shown are the amount adjusted for each of the 650 elements in the 12 × 4 subarrays (in the X and Y directions, respectively) of the mouse cDNA microarray used in the study. A location depended effect can be observed as a general increase over the length of the slide (bottom to top) and a dip near the center of the slide.

Figure 3 displays the data for one of the hybridizations in the aquaporin-1 dataset, both before and after normalization. As is common with two dye hybridizations, pre-normalized data shows curvature of the data at lower intensities (Figure 3A). Following lowess normalization for both intensity and position on the array, the curvature (intensity bias) of the plotted data is removed and the distribution of the data is narrowed (spatial bias) as compared to the pre-normalized data (Figure 3D). Our observations indicate that the location dependent effect is due to spatial hybridization variability and spatial scanning biases often caused by photo bleaching. In practice, the location of an element on the array can play a significant role in normalization, particularly with epifluorescence-based scanners (unpublished observation), and can vary by as much as a factor of 2 from one end of the slide to the other (Figure 3E). The extent to which location affects the intensity measurements is specific to each hybridization and scanning process.

### Variance shrinking

Microarray datasets usually contain thousands of elements, each representing one gene, but only a few measurements for each element. Whereas performing a global ANOVA on the entire dataset assumes equal variance within the data for each gene, it is generally accepted that independent analysis of the subset of data for each gene does not utilize sufficient data to determine an adequate representation of the variance associated with each gene. On the other hand, the major consequence of performing a gene-by-gene ANOVA is the overestimation of the significance of the calculated differences in expression for genes with abnormally small variances, and the underestimation of the significance of the calculated differences in expression for genes with abnormally large variances. Researchers have addressed these issues by including information from all genes on the array when assessing the significance of differences in expression for each gene [30-34]. CARMA calculates four variances and associated p-values for each gene[35]: gene specific variance, pooled variance (average for all genes), half of the gene and half of the pooled variance, and an estimator based on the James-Stein-Lindley shrinkage concept[36] that uses a formula to calculate the variance based on both the gene and pooled variances.

### Data filtering and outlier detection

Given that the samples hybridized to most large microarrays will contain transcripts for only a subset of the genes represented on the microarray, removing spots that exhibit low intensities for all samples can both reduce the number of genes incorrectly identified as differentially expressed between samples and decrease the computing time required for subsequent analyses. As implemented in CARMA, the ANOVA is usually (based on typical user settings) performed on only those genes that have background subtracted intensities greater than 1 or 2 background standard deviations for at least 51% of the measurements for at least one sample in the hybridization scheme. Furthermore, in the case of microarrays with replicate elements, usually at least 51% of the replicates must have background subtracted intensities greater than 1 or 2 background standard deviations in order for any of the replicates to be included in the ANOVA. In other words, the ANOVA is only performed on genes that are consistently expressed at measurable levels in at least one of the samples.

CARMA also has the capability to remove anomalous measurements through outlier detection. Dust, impurities, surface inhomogeneities, fluorochrome-specific effects, local hybridization effects, technician effects, etc. all contribute to non-systematic variability in the measured intensities. Anomalous measurements can be identified by their incongruity with other measurements within a hybridization and through inconsistencies between replicated measures[22,27]. Following the ANOVA of the normalized data for a gene, CARMA applies the following formula to identify outliers:

*r*_ti _*> |quantile*((*OP/2*)*/n, df -1*)*|*

**Figure 4:**
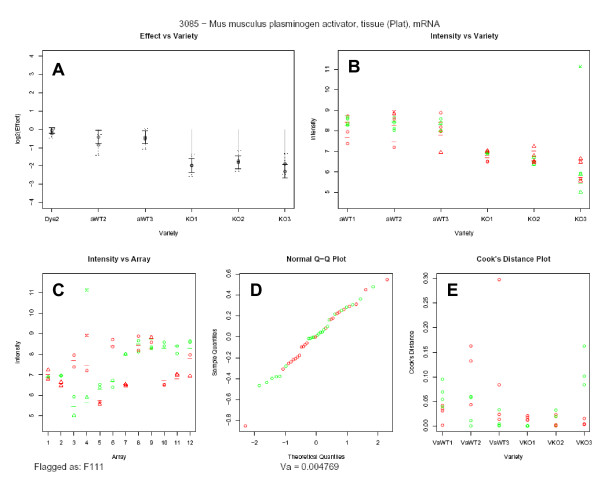
Results of ANOVA for the Mus Musculus *Plat *gene. In panels B-E of this figure the color of the plotted data points represents the fluorochrome that was used to label the sample (green = Alexa 546, Red = Alexa 647). **A **Graphical display of the Variety term estimate and standard error for the relative *Plat *gene expression for mice WT2, WT3, KO1, KO2, KO3 referenced to WT1 (the a in front of WT1, WT2 and WT3 is simply a label marker). Solid lines represent the final estimates after removal of outliers, while the dashed lines represent estimates before removal of outliers. The Dye2 (Alexa 647) effect and its standard error are also shown. **B, C **Transformed and normalized intensities plotted by sample (**B**) or hybridization (**C**). Colored circles (confident) and triangles (below user defined confidence threshold) represent the normalized measured intensities for each element (i.e. spot), and dashes represent the calculated intensities from the ANOVA model. An x denotes a point that was identified as an outlier. **D **A normal Q-Q plot for all data providing an indicator of the normality of the residuals. **E **The Cook's distance plot illustrating the influence of each data point on the fit of the model.

Where r_ti _= studentized residual of the ith element, OP = user defined outlier probability, n = the number of measurements, and df = degrees of freedom. ANOVA is performed recursively, removing the most extreme outlier after each step, until all outliers (which meet the criteria above) have been removed. The results of both the original ANOVA (using all data points), and the last ANOVA, performed on the dataset with all outliers removed, are shown in the graphical output (Figure 4A).

### Missing data

Most microarray hybridizations will have regions that are obviously problematic. While it may not be worthwhile to flag small abnormalities such as fine dust particles, large abnormalities should be flagged for exclusion from analysis. Missing data, whether flagged manually, filtered out, removed through outlier detection, or unavailable because of a failed hybridization may result in some experimental imbalance. This imbalance, however, is less problematic than including erroneous data. Therefore, CARMA utilizes functions that can accommodate missing data, to apply the linear model and perform the ANOVA.

### Output and display

Any analysis is only as good as its ability to portray relevant information to the researcher. In addition to generating tab delimited output files, CARMA creates an Adobe Portable Document Format (pdf) file containing easy to interpret graphical output (Figure 4) including an estimate, and its standard error, for each level (possible value) of the Variety effect, as well as plots of the normalized data and other statistical quality control information. This graphical output allows the researcher to refine and prioritize the list of differentially expressed genes by helping to identify cases of non-normality, outliers, unexpected patterns, etc. Visualizing the normalized data and the results of the ANOVA also helps to identify and correct cases of mislabeled samples and misaligned grids. All of the numbers used and generated by the ANOVA, and a file containing the contrasts between all pair-wise combinations of the levels of the Variety effect are also provided in delimited text files.

## Conclusion

Microarray experiments are intended to determine relative differences in gene expression between various treatments or conditions. As with nearly all experiments, and exacerbated by the number of measures obtained from microarray hybridizations, experimental noise can confound measurements and lead to the incorrect identification of random variations as significant differences in gene expression. We have employed a generalized approach and developed supporting software (CARMA) for performing ANOVA on microarray datasets that is easy to implement, generates readily interpretable graphical results, and accounts for experimental sources of variability. Applying ANOVA to microarray datasets incorporating replicated measures improves estimates of differences in gene expression between samples and provides a statistical basis for determining the significance of these differences. Also, because each sample is involved in multiple hybridizations, it is possible to identify and remove incongruous data points that are caused by dust particles, local background, etc, as well as allow for missing data caused by occasional failed or abnormal hybridizations. CARMA provides a clear quantitative and statistical characterization of each measured element on the microarray that can be used to assess marginally acceptable measures and improve confidence in the interpretation of microarray results. Overall, applying CARMA to microarray datasets incorporating replicated measures effectively reduces the number of gene incorrectly identified as differentially expressed and results in a more robust and reliable analysis.

In some situations it is advantageous to study the effects of more than one Variety on gene expression. For example, in gene knockout studies both genotype and treatment dependent effects may be important. Also, in cases where subjects are exposed to multiple treatments, accounting for individual-specific effects may expose differences in gene expression due to treatment that might otherwise be obscured. In these cases equation 1.4 can be modified to include terms for each of the varieties of interest. For example, the following model could be used to examine the effects of genotype (V1), drug treatment (V2), and the interaction between genotype and drug treatment (V1V2):

*i*_*ijlm *_*= μ +A*_*i *_*+ D*_*j *_*+ V*1_*l *_*+ V*2_*m *_*+ V*1*V*2_*lm *_*+ε *_*ijlm *_   (1.5)

Theoretically equation 1.5 could be expanded to include any number of varieties, however because of an exponential increase in the number of hybridization that must be performed most researchers limit their experiments to studying a maximum of two varieties.

Implementation of CARMA depends on a few assumptions. First, the lowess normalization assumes that the expression levels for the majority of genes in each sample are the same. While this is usually the case, even under conditions where the expression levels of the majority of genes are affected, lowess normalization produces the desired result of adjusting the dataset such that genes that behave dissimilarly from the majority are more likely to be identified as differentially expressed. Second, when determining the significance of differences in gene expression between samples, it is assumed that the data is normally distributed after the application of the linear model. We make this assumption in order to utilize the readily available R packages that perform ANOVA and their superior computational efficiency over non-parametric approaches. Lastly, in order to apply the ANOVA to each gene individually, it is assumed that after transformation and normalization each gene is independent of the other genes on each array. Theoretically, because each element (gene) is on every array there is not complete independence between genes, however we chose to implement the ANOVA on each gene independently in order to allow genes to have dissimilar variances, and to efficiently implement the ANOVA.

Background subtraction has been criticized for inflating the variance of microarray datasets, but this increased variability is almost always limited to elements that exhibit low intensity measures. This increased variability is not simply due to the smaller numbers, but rather the methods by which these numbers are measured and transformed. Both photomultiplier tubes (PMT) and charge-coupled devices (CCD) cannot accurately distinguish small differences in intensity. Therefore the assumption that error is multiplicative for these values is inaccurate and thus the application of a basic log-based transformation is inappropriate. The commonly seen flaring of the data at lower intensities, in either a simple green channel vs. red channel or a ratio-intensity plot, is not usually an indication of the inappropriate application of background subtraction, but rather an inappropriate transformation. In addition, not removing the relatively large uniform background associated with some datasets, such as those generated by CCD based scanners, can completely obscure the magnitude of any differences in gene expression. As illustrated by this dataset, different approaches to background subtraction and transformation can have a significant effect on the identification of differentially expressed genes. In fact, at most only 25 % the top 100 genes were shared between the background subtracted and non-background subtracted results.

Many statisticians are hesitant to remove outliers based solely on statistical criteria. In the case of microarray datasets, however, we know that there are invalid measurements in every dataset; yet it is often impractical to manually flag each instance. CARMA not only automatically detects and removes outliers, but also provides supporting graphs to assist in the final determination of the validity of the measurements that were removed. For example, in Figure 4, panels B and C, the green 'x' that indicates one of the excluded outliers is clearly separated from the rest of the measures (the corresponding red measurement is also excluded). Of course the researcher can also investigate each spot on the images from which the measurements were derived to further authenticate measurements flagged as outliers, or turn off outlier detection altogether if so desired. In addition to removing sporadic anomalous measurements, outlier detection has proven invaluable for detecting mislabeled samples and misaligned grids. In these cases, without outlier detection, all or a part of a microarray dataset is often labeled as too variable and of little value. A quick inspection of CARMA's graphical output reveals these problems as a series of genes for which the same hybridization's values were dropped as outliers, providing not only an indication that there is a problem, but also the exact hybridization that is the cause of the problem.

Because of the balanced design of this experiment we were able to assess the affects of inter-individual variability in the aquaporin-1 knockout vs. wildtype microarray dataset. The fact that 3% (129 out of 4361) of the confidently measured genes were identified as differentially expressed between mice of the same genotype highlights the significant amount of variability in gene expression between even genetically similar mice. This finding corroborates the results of an earlier study[37], underscoring the need for including biological replicates in any study, especially those addressing gene expression and molecular activities.

CARMA was designed to be an integrated, easy to use, analysis platform that researchers can apply to their microarray datasets without preprocessing their data or writing any computer code, with an emphasis on providing results in an easily interpretable format. In particular, we have attempted to identify and address issues such as the relatively high background and scanning related photobleaching that can occur with CCD based imaging systems, and include means to address many of the real-world problems associated with microarray experiments. There are already a number of existing microarray analysis tools that prove useful in analyzing microarray datasets [34,38,39]. CARMA implements many of the same statistical and analytical processes employed in these packages and includes the following additional features:

• Ability to read data files generated by most microarray image processing software

• No need to preprocess or combine raw data files

• Automatic exclusion of genes with low confidence measures for all samples

• Modified linlog transformation that better handles large negative numbers (that can result from background subtraction)

• Automatic computation of linlog crossover point

• Simultaneous intensity and location lowess normalization

• Ability to process incomplete datasets for fixed effect models

• Automatic outlier detection and removal

• Detailed graphical output for each gene

• Ability to detect and identify misaligned grids or mislabeled samples

The relative expression values generated by CARMA (i.e. the Variety value for each gene relative to a reference sample), can be further processed by commercial and freeware software packages designed to organize, cluster and display microarray data. In this regard, the relative expression values are analogous to the more traditional ratio-metric measures in that both provide a relative difference in expression between samples. Of course more advanced users can modify or extract portions of CARMA to integrate into their own analyses if so desired. Future development of CARMA may include developing a graphical user interface, improving the implementation of mixed models, adding new methods of normalization, implementing bootstrapping to calculate significance, and adapting CARMA to function as a Bioconductor[38] package.

## Availability and requirements

Project name: CARMA

Project home page: 

Operating system(s): Platform independent, only tested on Microsoft Windows 2000/XP

Programming language: R

Other requirements: R version 1.9 or higher

License: GNU General Public License

Restrictions to use by non-academics: Permission from University of Arizona required

## Authors' contributions

KG designed and developed the software and drafted the manuscript. MM performed all experiments related to the aquaporin-1 experimental dataset. HB conceived of the aquaporin-1 study and participated in its design and coordination. JH participated in the conceptualization, design, review and testing of the software, and assisted in the preparation of the manuscript.

## Grants and support

This research was supported in part by a University of Arizona Foundation Award (HLB), the Diamond Family Foundation, PANDA (People Acting Now Discover Answers) (JBH), University of Arizona BIO5 Graduate Research Award, and NIH awards #DK064706 (HLB) and #HL67067 (JBH).

## Supplementary Material

Additional File 1 **CARMA User Document.doc **A Microsoft Word document describing how to install and use the CARMA software. Click here for file

Additional File 2 **CARMA_5.0.zip **An R package for installing CARMA under the Microsoft Windows operating system. Click here for file

Additional File 3 **CARMA_Source.zip **The R source code for CARMA. Click here for file

Additional File 4 **CARMAAquaporin1.zip **The configuration files used to process the aquaporin-1 example dataset using CARMA. Click here for file

Additional File 5 **Data1.zip **Comma separated value data files containing the microarray experimental data. Click here for file

Additional File 6 **Data2.zip **Comma separated value data files containing the microarray experimental data. Click here for file

Additional File 7 **Data3.zip **Comma separated value data files containing the microarray experimental data. Click here for file

Additional File 8 **Data4.zip **Comma separated value data files containing the microarray experimental data. Click here for file

## Figures and Tables

**Table 1:** Comparison of background and transformation techniques

**Number of Genes**	**Background Subtracted Linlog Transformed**	**Background Subtracted Log2 Transformed**	**Non-Background Subtracted Log2 Transformed**	**Non-Background Subtracted Linlog Transformed**
10	778	375	565	565
100	3091	1464	2087	2087
1000	7084	3565	5019	5019

The sum of the gene Variety term ANOVA *F *values for the first 10, 100, or 1000 genes ranked by Variety term ANOVA *F *value. Larger numbers represent higher ratios of the between mouse variance over the within mouse variance.
