# Supplementary material for: CARMA: A platform for analyzing microarray datasets that incorporate replicate measures
Source: BMC Bioinformatics. 2006 Mar 17;7:149. doi: 10.1186/1471-2105-7-149 (PMC1450302; doi:10.1186/1471-2105-7-149)
Supplement: Additional File 1 — CARMA User Document.doc A Microsoft Word document describing how to install and use the CARMA software. [file 1471-2105-7-149-S1.doc]

# CARMA User Manual

###### Version 5.0

###### April 22, 2005

# Introduction

CARMA is an R package that was developed to provide a user-friendly method to analyze two-channel microarray datasets. This document is contains a description of how to analyze your dataset using CARMA. For a more detailed description of theory behind CARMA please refer to the accompanying article “CARMA: A platform for analyzing microarray datasets that incorporate replicate measures”.

CARMA was originally designed to analyze the microarray datasets generated by our laboratory, however the techniques employed for each aspect of the analysis were chosen for their broad applicability to two-channel microarray datasets. The main functions performed by CARMA are: reading in the data files, transformation, normalization, performing an analysis of variance (including contrasts), and generating both tab delimited files and a graphical representation of the results. User-defined parameters are used to tailor the analysis for dataset. Other features include data filtering, outlier detection and removal, and the ability to process incomplete datasets.

# System Requirements

CARMA was designed and developed under the Microsoft Windows 2000 operating system, however it was designed to be operating system independent. It has also been tested under Microsoft Windows XP. A precompiled version is only available for Microsoft Windows. In addition, the R programming environment (version 2.0 or later) must be installed, as well as the R “nlme” package. At least 1 GB of free hard drive space and 512 MB of RAM are also recommended.

# Installation

CARMA is a package for the R computing environment. If you do not already have R installed please visit [http://www.r-project.org](http://www.r-project.org/). In addition, you must have installed the *nlme* packeage, which can either be downloaded from the R website, or installed through R using the *install.packages* command (Ex. type *install.packages(“nlme”)* at the r command prompt).

CARMA is available at <http://www.u.arizona.edu/~jhoying/CARMA>. Currently a precompiled version of CARMA is only available for Microsoft Windows. To install CARMA, download CARMA.zip and select *Install package(s) from local zip files…* from the *Packages* dropdown menu. If you are using a different operating system then you can download the source file CARMA_Source.zip and either build a package on your system (if you are a very experienced R user), or you can load the file manually using the R source command (Ex. type *source(C:/CARMA/CARMA.R)* at the r command prompt, assuming that you have unzipped the CARMA.R file to the c:/CARMA directory).

# Known Issues

The following is a list of known issues and their solution.

## Error in scan … line ? did not have x elements

This error can be caused by one of two problems. This error is caused by one of the input files not containing the correct number of tabs. Usually the file is the Experiment file. To correct the problem open the file in a word processor that can display tabs, and make sure that each line has the same number of tabs.

# Data

CARMA was designed to read data from the individual data files (one for each hybridization) generated by most image analysis (spot finding) software packages. The user specifies a formula to calculate the spot intensities used in the ANOVA. CARMA will read the data and perform the calculations for each file (hybridization) in the experiment.

# Input

CARMA utilizes four input files in addition to the data files. Two of these files specify information about the array that was used in the experiment, and two files provide information specific to the experiment. Each of the files, a description of their use, and a description of each field are provided below. All files should be tab delimited. Also, all of these files should be tab delimited. Some characters, including a single quotation (‘) mark and the number sign (#), will cause an error if they exist inside of the file. Please remove or replace these characters if they exist in any of the input files.

## ArrayLayout.txt

This file is specific to a microarray, but is common to all experiments that use the microarray. In other words, if I have a microarray that contains 15,000 mouse cDNA clones, I would use the same files for all of the hybridizations that I performed that used this microarray, but I would use a different file for hybridizations involving a different microarray (for example a 12,000 cDNA rat microarray). This file is used to specify information about each spot on the microarray, and is used to describe what expressed sequence tags (which represent genes) are present on the array and how they are arranged on the array. The layout file is dependent on the orientation of the scanned image (i.e. a vertical image would have a different layout than a horizontal image). The location (SetRow, SetColumn, Row, Column) for each element should be specified from the upper left hand corner of the image. In addition to the required fields listed below, user-defined fields can also be provided. These fields will be included in some of the output files, and often include information such as the print plate location, length, or blast e-values for each spot.

| Field Name | Data Type | Description |
| --- | --- | --- |
| Gene | Numeric | A unique identifier (number) for each sequence printed on the array. Duplicate spots should have the same Gene id. |
| SetRow | Numeric | The row of the set (print grid). This value should be between 1 and 4 for vertical images, and 1 and 12 for horizontal images. |
| SetColumn | Numeric | The column of the set (print grid). This value should be between 1 and 12 for vertical images, and 1 and 4 for horizontal images. |
| Row | Numeric | The row of the spot within the print grid. |
| Column | Numeric | The column of the spot within the print grid. |
| Accession | Alpha-Numeric | The accession number for known sequences. This field is often hyperlinked to the NCBI website. |
| Description | Alpha-Numeric | A description of the contents of the spot. Usually a description of the gene if the sequence is known. |

## ArrayParameter.txt

This file is specific to a microarray and the data file generated by the spotfinding software, but is common to all experiments that use the microarray. In other words, if I have a microarray that contains 15,000 mouse cDNA clones, I would use the same files for all of the hybridizations that I performed that used this microarray, but I would use a different file for hybridizations involving a different microarray (for example a 12,000 cDNA rat microarray). This file contains information specific to the array, but common to all spots on the array. Also, it defines the names of columns in the data files that are necessary for the analysis.

| **Field Name** | **Data Type** | **Description** |
| --- | --- | --- |
| DataFileSeperator | Alpha-Numeric | The character that seperates fields in the data files. The tab character should be entered as \t. |
| ReplicateCount | Numeric | Number of replicates for each spot on the array. These replicate spots do no need to be adjacent to each other, however each spot must have the same number of replicates. |
| RowSpacing | Numeric | The spacing in microns between spot rows. |
| ColumnSpacing | Numeric | The spacing in microns between spot columns. |
| SetRowSpacing | Numeric | The spacing in microns between set (print grid) rows. |
| SetColumnSpacing | Numeric | The spacing in microns between set (print grid) columns. |
| SkipRows | Numeric | The number of rows to skip at the beginning of each data file. |
| Ch0Flag | Alpha-Numeric | The name of the Flag field in the hybridization data files. Characters, including spaces, other than number or characters should be entered as a ".". |
| Ch1Status | Alpha-Numeric | The name of the Channel 1 Detection Status field in the hybridization data files. Characters, including spaces, other than number or characters should be entered as a ".". |
| Ch1Spot | Alpha-Numeric | The name of the Channel 1 Spot Intensity field in the hybridization data files. Characters, including spaces, other than number or characters should be entered as a ".". |
| Ch1Background | Alpha-Numeric | The name of the Channel 1 Background Intensity field in the hybridization data files. Characters, including spaces, other than number or characters should be entered as a ".". |
| Ch1BackgroundSD | Alpha-Numeric | The name of the Channel 1 Background Standard Deviation field in the hybridization data files. Characters, including spaces, other than number or characters should be entered as a ".". |
| Ch2Status | Alpha-Numeric | The name of the Channel 2 Detection Status field in the hybridization data files. Characters, including spaces, other than number or characters should be entered as a ".". |
| Ch2Spot | Alpha-Numeric | The name of the Channel 2 Spot Intensity field in the hybridization data files. Characters, including spaces, other than number or characters should be entered as a ".". |
| Ch2Background | Alpha-Numeric | The name of the Channel 2 Background Intensity field in the hybridization data files. Characters, including spaces, other than number or characters should be entered as a ".". |
| Ch2BackgroundSD | Alpha-Numeric | The name of the Channel 2 Background Standard Deviation field in the hybridization data files. Characters, including spaces, other than number or characters should be entered as a ".". |

## Experiment.txt

This file is specific to one microarray experiment. It defines the samples that were used in each hybridization of the experiment. Up to 4 attributes (varieties) can be specified for each sample in each hybridization, although in practice only 1 or 2 are usually used. These attributes are used in the analysis of variance. For example a researcher might be interested in the effect of drug dosage and time on gene expression. The values for each attribute are entered in pairs (one for each channel). For example, the one might enter the values of t8h, t16h, d10, d100 in the Va1, Va2, Vb1, and Vb2 fields respectively, to specify that the cy3 sample was treated with 10ug of a drug for 8 hours, and the cy5 sample was treated with 100 ug of a drug for 16 hours.

| Field Name | Data Type | Description |
| --- | --- | --- |
| FileName | Alpha-Numeric | The name of the data file for a hybridization. |
| Va1 | Alpha-Numeric | The value of the first attribute for channel 1 in the hybridization. |
| Va2 | Alpha-Numeric | The value of the first attribute for channel 2 in the hybridization. |
| Vb1 | Alpha-Numeric | The value of the second attribute for channel 1 in the hybridization. |
| Vb2 | Alpha-Numeric | The value of the second attribute for channel 2 in the hybridization. |
| Vc1 | Alpha-Numeric | The value of the third attribute for channel 1 in the hybridization. |
| Vc2 | Alpha-Numeric | The value of the third attribute for channel 2 in the hybridization. |
| Vd1 | Alpha-Numeric | The value of the fourth attribute for channel 1 in the hybridization. |
| Vd2 | Alpha-Numeric | The value of the fourth attribute for channel 2 in the hybridization. |

## Input.txt

This file is specific to one microarray experiment, however most values will be the same for all experiments. The purpose of this file is to tailor the analysis for each experiment. A more detailed description of many of the fields is provided after the following table. Also, please refer to the Example section of this document for an illustration of how this file is used.

| Field Name | Data Type | Description |
| --- | --- | --- |
| Version | Numeric | Version of CARMA. |
| ExperimentFile | Alpha-Numeric | Name and path of the file that defines the attributes of the samples used in each hybridization. |
| ExportDefinitionFile | Alpha-Numeric | Name and path of the file that contains the list of columns to be exported for a data repository. |
| LayoutFile | Alpha-Numeric | Name and path of the file that defines the layout of the array used in all of the hybridizations. |
| ParameterFile | Alpha-Numeric | Name and path of the file that defines the parameters for the array used in all of the hybridizations. |
| DataDirectory | Alpha-Numeric | Path of the directory containing the hybridization data files. |
| OutputDirectory | Alpha-Numeric | Path of the directory to which the output will be written. |
| IntensityFormula | Alpha-Numeric | Formula to calculate the spot intensity. |
| MinimumIntensityFormula | Alpha-Numeric | Formula used to calculate the cutoff between the log and linear portion of the linlog transformation. |
| CriteriaFormula | Alpha-Numeric | Formula that defines the minimum criteria that needs to be met for in order for a spot to be considered a good measure. |
| ExcludeFormula | Alpha-Numeric | Formula for the criteria used to exclude data. |
| ReplicatePercent | Numeric | Percentage of replicates that need to pass the criteria in order for any of the replicates to be used in the analysis. |
| SpotPercent | Numeric | The minimum percentage of spots that must pass the criteria formula in order for the gene to be analyzed. |
| AnovaFormula | Alpha-Numeric | ANOVA formula. |
| ReplicationFormula | Alpha-Numeric | Replication formula. |
| LoessFormula | Alpha-Numeric | Lowess regression formula. |
| LoessSpan | Numeric | Percentage of data points used for the lowess regression. |
| OutlierProbability | Numeric | The probabilty at which to remove outliers. |
| AnovaProbability | Numeric | Maximum p-value for a gene to be selected. |
| Effect | Numeric | Minimum difference (log base 2) between any two levels of a variety for a gene to be selected. |

The following bullets contain a more detailed description and common values for many of the fields in the above table.

**IntensityFormula** – This formula describes how the intensity value used in the ANOVA is calculated. This formula is described based on the fields in the ArrayParameter.txt file (without the Ch1 or Ch2 prefix), which currently include: Status, Spot, Background, and BackgroundSD. The most common values for this field are *Spot* for non-background subtracted data, and *Spot-Background* for background subtracted data.

#### MinimumIntensityFormula – This formula is used to describe the cutoff between the log and linear portions of the linlog transformation. The most common values are: *BackgroundSD*, which sets the cutoff to the median of one standard deviation of the background of all spots on the array; *Background*, which sets the cutoff to the median of the background of all spots on the entire array; *Optimize*, which attempts to optimize the cutoff based on the assumption of equal variance over the entire range of intensities; and *1*, which sets the cutoff to 1.

**CriteriaFormula** – This formula defines the criteria for a spot to be identified as a reliable measure. Common values are: *BackgroundSD*, which sets the criteria to the median of one standard deviation of the background of all spots on the array; and *Background*, which sets the cutoff to the median of the background of all spots on the entire array; or *1* which designates all spots as reliable measures.

#### ExcludeFormula – This formula defines the criteria to exclude spots from the analysis. Common values are: *Flag > 0*, which excludes all spots for which the flag is greater than 0; or *Flag <> Normal*, which excludes all spots for which the flag is not equal to NORMAL.

**ReplicatePercent** – The percentage of replicates of a element on the array that must pass the CriteriaFormula for the any of the replicates to be used in the ANOVA. For example, if the microarray that I am using is printed in triplicate, and my ReplicatePercent was set to .51, then for any given element at least two of the three spots must be identified as reliable measures (based on the CriteriaFormula) and not be excluded (based on the ExcludeFormula) in at least one of the channels, for any of the measures for the three spots to be include in the ANOVA. The most common value is *.51*.

**SpotPercent** – The percentage of measurements for a given element on the array that must be identified as reliable and non-excluded (based on the CriteriaFormula, ReplicatePercent, and ExcludeFormula). The most common value is *.51*. This percentage essentially defines the percentage of times an element must be measured reliably for the element to be analyzed in the ANOVA. For example, if I was using an array printed in triplicate, and each sample in my hybridization scheme were hybridized 4 times, then for each element on the array I would have 12 measurements per sample. If my SpotPercent were set at .51, then 7 of those measurements for at least one sample would have to be identified as reliable.

**AnovaFormula** – The formula used to fit the data in the ANOVA. The available terms for this formula are I (Intensity), A (Array), D (Dye), Va (Variety a), Vb (Variety b), Vc (Variety c), Vd (Variety d). The left side of the formula is always I and the left and right sides of the formula are separated by a ~. The most common values are: *I~A+D+Va*, which is used for experiments involving one variety (variable of interest); *I~A+D+Va+Vb*, which is used for experiments involving two varieties (variables of interest) without an interaction term; or *I~A+D+Va+Vb+VaVb*; which is used for experiments involving two varieties (variables of interest) with an interaction term.

#### ReplicationFormula – This formula defines the replication used in the microarray experiment. The most common values are: *R*, if the only replication is replicate spots for each element on the array; *Vb*, if there is replication at the sample level; or *Vb/R* if there is replication at the sample and array levels.

#### LoessFormula – The formula for the locally weighted regression used to normalize the data. The available terms are: M (the product of the intensities of the two channels), A (the ratio of the intensities of the two channels), R (row), C (column). The most common values are: *M~A*, for an intensity based loess normalization; *M~R+*C, for a location based loess normalization, M*~A+R+C*, for a intensity and location based loess normalization; *M~A+R*, for an intensity an row based loess normalization; *M~A+C*, for an intensity and column based normalization.

**LoessSpan** – The percentage of spots used in the locally weighted regression. Common values range from .2 to .4.

**OutlierProbability** – The probability at which to remove outliers. Common values are: *.05*, 95% confidence that a spot is an outlier is required for it to be excluded from use in the ANOVA; *.01*, 99% confidence that a spot is an outlier is required for it to be excluded from use in the ANOVA; *0*, no spots are excluded from use in the ANOVA based on the outlier calculation.

**AnovaProbability** – The maximum *p*-value for the ANOVA variety term allowable for an element on the array to be included in the list of selected elements. If there is more than one variety in the linear model defined for the ANOVA, then if either variety has a *p*-value less than the value defined in this field then the element is flagged as selected. Common values range from .05 to .001.

**Effect** – The magnitude (in the log_2_ scale) of the difference between any two levels of the variety (i.e. Difference between any two treatements) that is necessary for an element to be included in the list of selected elements. Common values are: *.585*, or *1*, *0* which correspond to a 1.5 fold change, a 2 fold change, or no fold change criteria, respectively.

# Output

## Normalized.txt

This file contains the normalized intensities for all hybridization in the experiment. It also has a Pass columns indicating whether each intensity measurement was identified as a reliable.

| Field Name | Description |
| --- | --- |
| Number | The spot number. |
| Gene | The gene (element) number. |
| Column | The column of the spot in microns calculated from the SetColumn and Column. |
| Row | The row of the spot in microns calculated from the SetRow and Row. |
| V… | A series of columns that start with the letter V, which contain the normalized intensity for one channel, with a separate row for each spot. There is one column for each channel of each array. They are organized in the order that the data files are listed in the experiment.txt file, with the two channels for each hybridization adjacent to each other. |
| Pass…. | A series of columns that start with the word Pass, each row contains a 1 if the spot passes the criteria to be considered reliable, otherwise it contains a 0. There is one column for each channel of each array and the columns are ordered the same way as the V… columns. |

## NormalizedMean.txt

This file contains the average normalized intensities by element for all replicate spots for all hybridization in the experiment. It also has Pass columns that average the Pass values for all replicate spots for each element.

| Field Name | Description |
| --- | --- |
| Gene | The gene (element) number. |
| Mean | The mean normalized intensity for all measures for the array element. |
| V… | A series of columns that start with the letter V, which contain the normalized intensity for one channel, with a separate row for each element (average of replicate spots). There is one column for each channel of each array. They are organized in the order that the data files are listed in the experiment.txt file, with the two channels for each hybridization adjacent to each other. |
| Pass…. | A series of columns that start with the word Pass, each row contains the average of the spot values (see Normalized.txt) for all replicate spots of an element. There is one column for each channel of each array and the columns are ordered the same way as the V… columns. |

## Output.txt

This file contains the calculated values for each level of the variety effect. For example, if we were analyzing the effect of four different dosages (10, 20, 40, 80) of a drug on gene expression there would be three calculated values Value.20, Value.40, and Value.80. Each of these values is a calculation (reported as log_2_) of how the corresponding dosage affected gene expression relative to the lowest dosage level (10). This file also contains measures of the significance (*p­­*-value) of the Variety term and calculations of the standard error associated with each level of the Variety Effect.

| Field Name | Description |
| --- | --- |
| Gene | The gene (element) number. |
| Accession | The accession number for known sequences. This field is often hyperlinked to the NCBI website. This field is copied from the ArrayLayout.txt file. |
| Description | A description of the element on the array. Usually a description of the gene if the sequence is known. This field is copied from the ArrayLayout.txt file. |
| Flag | This field consists of an F followed by three binary (0 or 1) numbers. The first number is an indicator of whether an ANOVA was performed for the element. The second number is whether the element passed the fold change criteria. The third number is whether the element passed the ANOVA *p*-value criteria. |
| ResidualDF | The residual degrees of freedom from the analysis of variance. |
| ANOVAp | The *p*-value for the variety term (Va) in the ANOVA. This *p*-value is calculated based on the variance of the measurements for just the one array element. |
| ShrunkANOVAp | The *p*-value for the variety term (Va) in the ANOVA. This *p*-value is calculated based on the James-Stein-Lindley shrinkage concept and includes part of the variance of the measurements for just the one array element and part of the average of the variance of the measurements for all elements on the array. |
| MixANOVAp | The *p*-value for the variety term (Va) in the ANOVA. This *p*-value is calculated based on half of the variance of the measurements for just the one array element and half of the average of the variance of the measurements for all elements on the array. |
| PoolANOVAp | The *p*-value for the variety term (Va) in the ANOVA. This *p*-value is calculated based on the average of the variance of the measurements for all elements on the array. |
| Mean | The mean normalized intensity for all measures for the array element. |
| Value… | This series of columns contains the calculated values for each level of the variety effect. For example, if we were analyzing the effect of four different dosages (10, 20, 40, 80) of a drug on gene expression there would be three columns Value.20, Value.40, and Value.80. Each of these columns is a calculation (reported as log_2_) of how the corresponding dosage affected gene expression relative to the lowest dosage level (10). |
| Unlog(Value… | This series of columns is the same as the Value… columns, except that the numbers transformed from the log scale to the linear scale. Down regulation, however, is transformed to a negative number instead of a fraction. |
| StdErr… | This series of columns contains the calculated standard errors that are associated with each of the Value… columns. |

## OutputSelect.txt

This file is the same as the Output.txt file, however it only contains elements that were identified as differentially expressed based on the AnovaProbability and Effect parameters specified in the Input.txt file.

## AnovaContrast.txt

| Field Name | Description |
| --- | --- |
| Gene | The gene (element) number. |
| Accession | The accession number for known sequences. This field is often hyperlinked to the NCBI website. This field is copied from the ArrayLayout.txt file. |
| Description | A description of the element on the array. Usually a description of the gene if the sequence is known. This field is copied from the ArrayLayout.txt file. |
| Flag | This field consists of an F followed by three binary (0 or 1) numbers. The first number is an indicator of whether an ANOVA was performed for the element. The second number is whether the element passed the fold change criteria. The third number is whether the element passed the ANOVA *p*-value criteria. |
| ResidualDF | The residual degrees of freedom from the analysis of variance. |
| ANOVAp | The *p*-value for the variety term (Va) in the ANOVA. This *p*-value is calculated based on the variance of the measurements for just the one array element. |
| ShrunkANOVAp | The *p*-value for the variety term (Va) in the ANOVA. This *p*-value is calculated based on the James-Stein-Lindley shrinkage concept and includes part of the variance of the measurements for just the one array element and part of the average of the variance of the measurements for all elements on the array. |
| MixANOVAp | The *p*-value for the variety term (Va) in the ANOVA. This *p*-value is calculated based on half of the variance of the measurements for just the one array element and half of the average of the variance of the measurements for all elements on the array. |
| PoolANOVAp | The *p*-value for the variety term (Va) in the ANOVA. This *p*-value is calculated based on the average of the variance of the measurements for all elements on the array. |
| Mean | The mean normalized intensity for all measures for the array element. |
| p… | This series of columns contains the calculated *p*-values for the ANOVA contrasts (*t*-tests) between each of the combinations of variety levels. For example, in the case of an experiment that compared four dosage levels of a drug (10, 20, 40, 80), there would be 6 columns (p.20, p.40, p.80, p.20-40, p.20-80, p.40-80). These columns represent the following comparisons respectively: 10 vs. 20, 10 vs. 40, 10 vs. 80, 20 vs. 40, 20 vs. 80, 40 vs. 80. |
| Value… | This series of columns contains the calculated values for each level of the variety effect. For example, if we were analyzing the effect of four different dosages (10, 20, 40, 80) of a drug on gene expression there would be three columns Value.20, Value.40, and Value.80. Each of these columns is a calculation (reported as log_2_) of how the corresponding dosage affected gene expression relative to the lowest dosage level (10). |
| StdErr… | This series of columns contains the calculated standard errors that are associated with each of the Value… columns. |
| p.Mix... | This series of columns identical to the p… columns with the exception of the standard error that is used in the calculation of the *p*-value. For these columns, the standard error is calculated based on half of the variance of the measurements for just the one array element and half of the average of the variance of the measurements for all elements on the array. |

## AnovaContrastSelect.txt

This file is the same as the Output.txt file, however it only contains elements that were identified as differentially expressed based on the AnovaProbability and Effect parameters specified in the Input.txt file.

## PlotNormalize.pdf

This file contains graphs of the data for each hybridization both before and after normalization. There is one page for between channel normalization for each hybridization and one page for between array normalization for each hyrbridization.


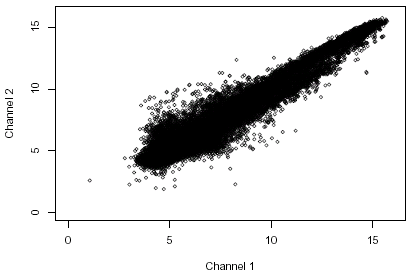

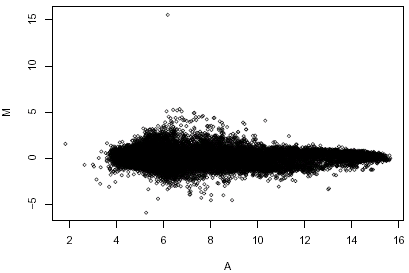

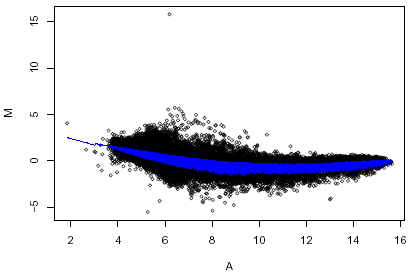

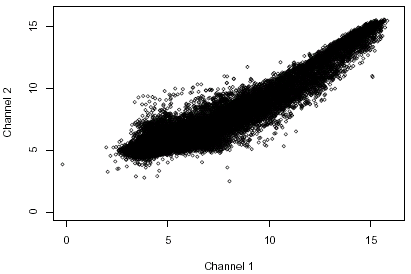


**A**

**B**

**C**

**D**

Normalization of a two-channel hybridization. **A** The linlog transformed data before normalization plotted as channel 1 (Alexa 546 dye) versus channel 2 (Alexa 647 dye) intensities. **B**,**C** Ratio-Intensity plot before (**B**) and after (**C**) spatial and intensity lowess normalization. “M” refers to the log ratio of the two channels and “A” refers to the geometric mean of the spot intensity for both channels. The blue line in **B** is the spatial/intensity lowess normalization fitted curve, with the curvature of the line representing the intensity-dependent fit and the width of the line representing the spatial component of the lowess normalization. **D** The final normalized data used for the ANOVA.

## Plot.pdf

This file contains one page for each expressed sequence tag (gene) on the array that was analyzed (only EST’s that had enough confident measures are analyzed). The following figure is for one gene in an experiment that compared the gene expression of 6 different mice. Three of the mice were wildtype (WT) and three were aquaporin-1 knockouts.


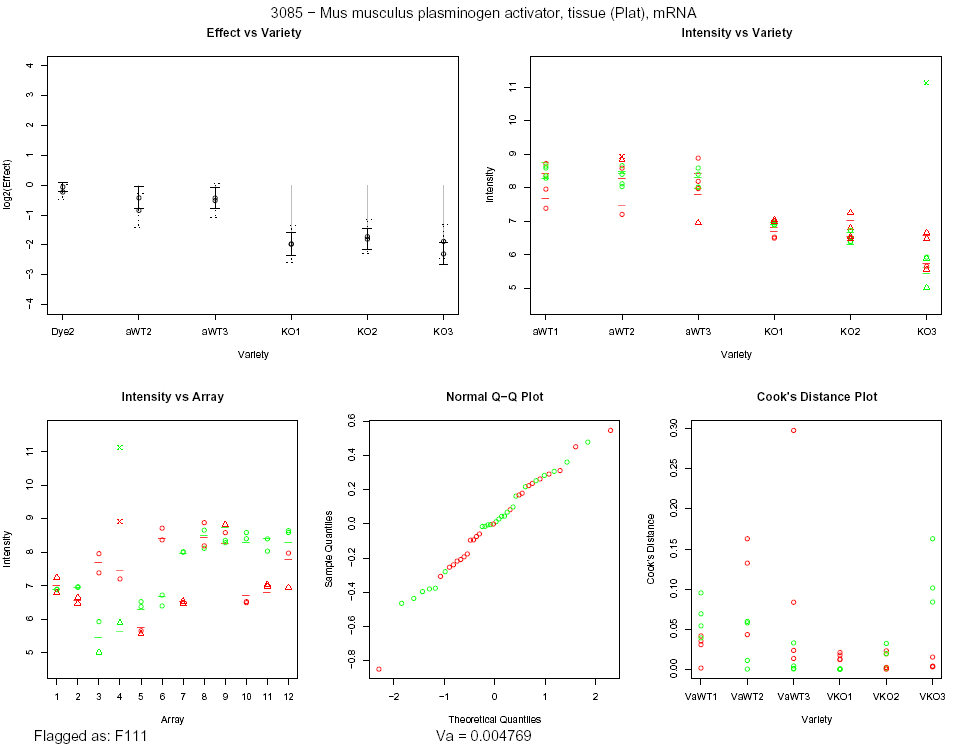


**A**

**B**

**C**

**D**

**E**

Results of ANOVA for the Mus Musculus *Plat* gene. In panels B-E of this figure the color of the plotted data points represents the fluorochrome that was used to label the sample (green = Alexa 546, Red = Alexa 647). **A** Graphical display of the Variety term estimate and standard error for the relative *Plat* gene expression for mice WT2, WT3, KO1, KO2, KO3 referenced to WT1 (the a in front of WT1, WT2 and WT3 is simply a label marker). Solid lines represent the final estimates after removal of outliers, while the dashed lines represent estimates before removal of outliers. The Dye2 (Alexa 647) effect and its standard error are also shown. **B,C** Transformed and normalized intensities plotted by sample (**B**) or hybridization (**C**). Colored circles (confident) and triangles (below user defined confidence threshold) represent the normalized measured intensities for each element (i.e. spot), and dashes represent the calculated intensities from the ANOVA model. An x denotes a point that was identified as an outlier. **D** A normal Q-Q plot for all data providing an indicator of the normality of the residuals. **E** The Cook’s distance plot illustrating the influence of each data point on the fit of the model.

## PlotSelect.pdf

This file is the same as the Plot.pdf file, however it only contains elements that were identified as differentially expressed based on the AnovaProbability and Effect parameters specified in the Input.txt file.

# Examples of Input Files

This section if divided into two parts. The first part contains examples of the files used to define the layout of the microarray and the data files generated by the spot finding software. These files are common to all experiments that use the same microarray and scanner/spotfinding software. The second part contains examples of the input files used to define specific experiments.

## ArrayLayout.txt – This example contains the first 10 rows of the layout file for a rat oligo array array. Each element on the array is printed in triplicate. In addition to the required fields, this file contains a location column that specified the plate location of each element on the array.

| **Gene** | **SetRow** | **SetColumn** | **Row** | **Column** | **Location** | **Accession** | **Description** |
| --- | --- | --- | --- | --- | --- | --- | --- |
| 1 | 1 | 1 | 1 | 1 | P001A01 |  |  |
| 1 | 1 | 1 | 1 | 2 | P001A01 |  |  |
| 1 | 1 | 1 | 1 | 3 | P001A01 |  |  |
| 2 | 1 | 1 | 1 | 4 | P001A13 | [NM_019905](http://www4.ncbi.nlm.nih.gov/htbin-post/Entrez/query?db=n&form=6&uid=NM_019905&dopt=g) | Calpactin I heavy chain |
| 2 | 1 | 1 | 1 | 5 | P001A13 | [NM_019905](http://www4.ncbi.nlm.nih.gov/htbin-post/Entrez/query?db=n&form=6&uid=NM_019905&dopt=g) | Calpactin I heavy chain |
| 2 | 1 | 1 | 1 | 6 | P001A13 | [NM_019905](http://www4.ncbi.nlm.nih.gov/htbin-post/Entrez/query?db=n&form=6&uid=NM_019905&dopt=g) | Calpactin I heavy chain |
| 3 | 1 | 1 | 1 | 7 | P001E01 | [NM_019385](http://www4.ncbi.nlm.nih.gov/htbin-post/Entrez/query?db=n&form=6&uid=NM_019385&dopt=g) | Golgi peripheral membrane protein p65 |
| 3 | 1 | 1 | 1 | 8 | P001E01 | [NM_019385](http://www4.ncbi.nlm.nih.gov/htbin-post/Entrez/query?db=n&form=6&uid=NM_019385&dopt=g) | Golgi peripheral membrane protein p65 |
| 3 | 1 | 1 | 1 | 9 | P001E01 | [NM_019385](http://www4.ncbi.nlm.nih.gov/htbin-post/Entrez/query?db=n&form=6&uid=NM_019385&dopt=g) | Golgi peripheral membrane protein p65 |

## ArrayParameter.txt – The following tables are examples of the ArrayParameter.txt file. Because this file can be dependent upon the type of scanner/software used, I have included two examples. These examples are both for a rat oligo array.

ArrayParamter.txt for ArrayWoRx scanner

| DataFileSeperator | , | The character that seperates fields in the data files. The tab character should be entered as \t. |
| --- | --- | --- |
| **ReplicateCount** | 3 | Number of replicates for each spot on the array. These replicate spots do no need to be adjacent to each other, however each spot must have the same number of replicates. |
| **RowSpacing** | 220 | The spacing in microns between spot rows. |
| **ColumnSpacing** | 220 | The spacing in microns between spot columns. |
| **SetRowSpacing** | 4500 | The spacing in microns between set (print grid) rows. |
| **SetColumnSpacing** | 4500 | The spacing in microns between set (print grid) columns. |
| **SkipRows** | 0 | The number of rows to skip at the beginning of each data file. |
| **Ch0Flag** | Flagged.As | The name of the Flag field in the hybridization data files. Special characters (i.e. not alphanumeric characters), including spaces, should be entered as a ".". |
| **Ch1Status** | Detection.Status..w595. | The name of the Channel 1 Detection Status field in the hybridization data files. Special characters (i.e. not alphanumeric characters), including spaces, should be entered as a ".". |
| **Ch1Spot** | Spot.Mean.Intensity..w595. | The name of the Channel 1 Spot Intensity field in the hybridization data files. Special characters (i.e. not alphanumeric characters), including spaces, should be entered as a ".". |
| **Ch1Background** | Background.Median.Intensity..w595. | The name of the Channel 1 Background Intensity field in the hybridization data files. Special characters (i.e. not alphanumeric characters), including spaces, should be entered as a ".". |
| **Ch1BackgroundSD** | Background.Standard.Deviation..w595. | The name of the Channel 1 Background Standard Deviation field in the hybridization data files. Special characters (i.e. not alphanumeric characters), including spaces, should be entered as a ".". |
| **Ch2Status** | Detection.Status..w685. | The name of the Channel 2 Detection Status field in the hybridization data files. Special characters (i.e. not alphanumeric characters), including spaces, should be entered as a ".". |
| **Ch2Spot** | Spot.Mean.Intensity..w685. | The name of the Channel 2 Spot Intensity field in the hybridization data files. Special characters (i.e. not alphanumeric characters), including spaces, should be entered as a ".". |
| **Ch2Background** | Background.Median.Intensity..w685. | The name of the Channel 2 Background Intensity field in the hybridization data files. Special characters (i.e. not alphanumeric characters), including spaces, should be entered as a ".". |
| **Ch2BackgroundSD** | Background.Standard.Deviation..w685. | The name of the Channel 2 Background Standard Deviation field in the hybridization data files. Special characters (i.e. not alphanumeric characters), including spaces, should be entered as a ".". |

ArrayParameter.txt for GenePix scanner – In this case there are no Status fields in the data files, so those columns should be set to the name of another existing column in the data files (in this case they were set to the Flags column).

| DataFileSeperator | , | The character that seperates fields in the data files. The tab character should be entered as \t. |
| --- | --- | --- |
| **ReplicateCount** | 3 | Number of replicates for each spot on the array. These replicate spots do no need to be adjacent to each other, however each spot must have the same number of replicates. |
| **RowSpacing** | 220 | The spacing in microns between spot rows. |
| **ColumnSpacing** | 220 | The spacing in microns between spot columns. |
| **SetRowSpacing** | 4500 | The spacing in microns between set (print grid) rows. |
| **SetColumnSpacing** | 4500 | The spacing in microns between set (print grid) columns. |
| **SkipRows** | 31 | The number of rows to skip at the beginning of each data file. |
| **Ch0Flag** | Flags | The name of the Flag field in the hybridization data files. Special characters (i.e. not alphanumeric characters), including spaces, should be entered as a ".". |
| **Ch1Status** | Flags | The name of the Channel 1 Detection Status field in the hybridization data files. Special characters (i.e. not alphanumeric characters), including spaces, should be entered as a ".". |
| **Ch1Spot** | F635.Mean | The name of the Channel 1 Spot Intensity field in the hybridization data files. Special characters (i.e. not alphanumeric characters), including spaces, should be entered as a ".". |
| **Ch1Background** | B635.Median | The name of the Channel 1 Background Intensity field in the hybridization data files. Special characters (i.e. not alphanumeric characters), including spaces, should be entered as a ".". |
| **Ch1BackgroundSD** | B635.SD | The name of the Channel 1 Background Standard Deviation field in the hybridization data files. Special characters (i.e. not alphanumeric characters), including spaces, should be entered as a ".". |
| **Ch2Status** | Flags | The name of the Channel 2 Detection Status field in the hybridization data files. Special characters (i.e. not alphanumeric characters), including spaces, should be entered as a ".". |
| **Ch2Spot** | F532.Mean | The name of the Channel 2 Spot Intensity field in the hybridization data files. Special characters (i.e. not alphanumeric characters), including spaces, should be entered as a ".". |
| **Ch2Background** | B532.Median | The name of the Channel 2 Background Intensity field in the hybridization data files. Special characters (i.e. not alphanumeric characters), including spaces, should be entered as a ".". |
| **Ch2BackgroundSD** | B532.SD | The name of the Channel 2 Background Standard Deviation field in the hybridization data files. Special characters (i.e. not alphanumeric characters), including spaces, should be entered as a ".". |

## Input.txt and Experiment.txt – The following examples of the experiment.txt and input.txt files are all for an experiment that involved a comparison between 3 aquaporin-1 knockout mice, and 3 wildtype mice. The experimental design consisted of RNA from each mouse being hybridized against four of the other mice, using a mouse cDNA array with each element printed in duplicate.

The following two tables were used to analyze the dataset for differences in gene expression between mice, irrespective of genotype. The a was added in front of WT in order that WT be alphabetically smaller than KO. This insured that mouse WT1 would be used as the reference.

Experiment.txt

| **FileName** | **Va1** | **Va2** | **Vb1** | **Vb2** | **Vc1** | **Vc2** | **Vd1** | **Vd2** |
| --- | --- | --- | --- | --- | --- | --- | --- | --- |
| Slide108rescan.csv | KO1 | KO2 |  |  |  |  |  |  |
| 126ko1Gko3R073003.csv | KO1 | KO3 |  |  |  |  |  |  |
| 113ko3Gcon1R072503.csv | KO3 | aWT1 |  |  |  |  |  |  |
| 112ko3Gcon2R072503.csv | KO3 | aWT2 |  |  |  |  |  |  |
| 111ko2Gko3R072503.csv | KO2 | KO3 |  |  |  |  |  |  |
| 110ko2Gcon1R072403.csv | KO2 | aWT1 |  |  |  |  |  |  |
| 107con3Gko2R072403.csv | aWT3 | KO2 |  |  |  |  |  |  |
| 106con2Gcon3R072403.csv | aWT2 | aWT3 |  |  |  |  |  |  |
| 105con1Gcon2R072403.csv | aWT1 | aWT2 |  |  |  |  |  |  |
| 103con3Gko1R072303.csv | aWT3 | KO1 |  |  |  |  |  |  |
| 102con2Gko1R072303.csv | aWT2 | KO1 |  |  |  |  |  |  |
| 101con1Gcon3R072303.csv | aWT1 | aWT3 |  |  |  |  |  |  |

Input.txt

| Version | 5 |
| --- | --- |
| **ExperimentFile** | .\Experiment.txt |
| **ExportDefinitionFile** | .\ExportDefinition.txt |
| **LayoutFile** | C:\ArrayLayouts\Mouse15K\Mouse15KLayoutScanTop.txt |
| **ParameterFile** | C:\ArrayLayouts\Mouse15K\Mouse15KParameter.txt |
| **DataDirectory** | ..\Data\ |
| **OutputDirectory** | ..\Output\ |
| **IntensityFormula** | Spot-Background |
| **MinimumIntensityFormula** | BackgroundSD |
| **CriteriaFormula** | ((Spot-Background) > (2*BackgroundSD)) & (Spot-Background)>1 |
| **ExcludeFormula** | Flag!="NORMAL" |
| **ReplicatePercent** | 0.51 |
| **SpotPercent** | 0.51 |
| **AnovaFormula** | I~A+D+Va |
| **ReplicationFormula** | R |
| **LoessFormula** | M~A+R+C |
| **LoessSpan** | 0.2 |
| **OutlierProbability** | 0.05 |
| **AnovaProbability** | 0.05 |
| **Effect** | 0.585 |

The following two tables were used analyze differences between the aquaporin-1 group and the wildtype group.

| **FileName** | **Va1** | **Va2** | **Vb1** | **Vb2** | **Vc1** | **Vc2** | **Vd1** | **Vd2** |
| --- | --- | --- | --- | --- | --- | --- | --- | --- |
| Slide108rescan.csv | KO | KO | KO1 | KO2 |  |  |  |  |
| 126ko1Gko3R073003.csv | KO | KO | KO1 | KO3 |  |  |  |  |
| 113ko3Gcon1R072503.csv | KO | aWT | KO3 | aWT1 |  |  |  |  |
| 112ko3Gcon2R072503.csv | KO | aWT | KO3 | aWT2 |  |  |  |  |
| 111ko2Gko3R072503.csv | KO | KO | KO2 | KO3 |  |  |  |  |
| 110ko2Gcon1R072403.csv | KO | aWT | KO2 | aWT1 |  |  |  |  |
| 107con3Gko2R072403.csv | aWT | KO | aWT3 | KO2 |  |  |  |  |
| 106con2Gcon3R072403.csv | aWT | aWT | aWT2 | aWT3 |  |  |  |  |
| 105con1Gcon2R072403.csv | aWT | aWT | aWT1 | aWT2 |  |  |  |  |
| 103con3Gko1R072303.csv | aWT | KO | aWT3 | KO1 |  |  |  |  |
| 102con2Gko1R072303.csv | aWT | KO | aWT2 | KO1 |  |  |  |  |
| 101con1Gcon3R072303.csv | aWT | aWT | aWT1 | aWT3 |  |  |  |  |

Input.txt

| Version | 5 |
| --- | --- |
| **ExperimentFile** | .\Experiment.txt |
| **ExportDefinitionFile** | .\ExportDefinition.txt |
| **LayoutFile** | C:\ArrayLayouts\Mouse15K\Mouse15KLayoutScanTop.txt |
| **ParameterFile** | C:\ArrayLayouts\Mouse15K\Mouse15KParameter.txt |
| **DataDirectory** | ..\Data\ |
| **OutputDirectory** | ..\Output\ |
| **IntensityFormula** | Spot-Background |
| **MinimumIntensityFormula** | BackgroundSD |
| **CriteriaFormula** | ((Spot-Background) > (2*BackgroundSD)) & (Spot-Background)>1 |
| **ExcludeFormula** | Flag!="NORMAL" |
| **ReplicatePercent** | 0.51 |
| **SpotPercent** | 0.51 |
| **AnovaFormula** | I~A+D+Va |
| **ReplicationFormula** | ~1|Vb/R |
| **LoessFormula** | M~A+R+C |
| **LoessSpan** | 0.2 |
| **OutlierProbability** | 0.05 |
| **AnovaProbability** | 0.05 |
| **Effect** | 0.585 |

## Mixed Effects Models – All of the examples presented so far have been fixed effects models. However, to get a better estimate of the significances of the difference in gene expression, a mixed effects model should be used in some cases. In the previous example, the Vb terms should really be specified as a random effect in the ReplicationFormula. Many statisticians also recommend specifying the array term as a random effect. Please refer to the help documentation for the random parameter of the r lme function for a more detailed description of the implementation of random effects.

## Multiple Variety Terms – The AnovaFormula field in the Input.txt file can be used to specify more than one (up to 4) variety (condition) term in the ANOVA. For example, if we wanted to investigate the effects of dosage and time in a microarray experiment, we would modify the AnovaFormula to read I~A+D+Va+Vb. If we also wanted to include a term for the interaction of dosage and time we would modify the AnovaFormula to read I~A+D+Va+Vb+VaVb

## Common Reference Designs – Many researchers perform microarray experiments where all experimental samples are hybridized against a common reference design. In this case, differences in gene expression between the common reference and the experimental samples are usually of no interest. These types of designs can be accommodated in CARMA by specifying the common reference as CARMAref in the Experiment.txt file, as in the following example.

| **FileName** | **Va1** | **Va2** | **Vb1** | **Vb2** | **Vc1** | **Vc2** | **Vd1** | **Vd2** |
| --- | --- | --- | --- | --- | --- | --- | --- | --- |
| Slide1.csv | CARMAref | Exp1 |  |  |  |  |  |  |
| Slide1.csv | Exp1 | CARMAref |  |  |  |  |  |  |
| Slide1.csv | CARMAref | Exp1 |  |  |  |  |  |  |
| Slide1.csv | Exp1 | CARMAref |  |  |  |  |  |  |
| Slide1.csv | CARMAref | Exp2 |  |  |  |  |  |  |
| Slide1.csv | Exp2 | CARMAref |  |  |  |  |  |  |
| Slide1.csv | CARMAref | Exp2 |  |  |  |  |  |  |
| Slide1.csv | Exp2 | CARMAref |  |  |  |  |  |  |
