# Supplementary material for: CARMA: A platform for analyzing microarray datasets that incorporate replicate measures
Source: BMC Bioinformatics. 2006 Mar 17;7:149. doi: 10.1186/1471-2105-7-149 (PMC1450302; doi:10.1186/1471-2105-7-149)
Supplement: Additional File 2 — CARMA_5.0.zip An R package for installing CARMA under the Microsoft Windows operating system. [file 1471-2105-7-149-S2.zip › CARMA/html/00Index.html]

R: CARMA

# CARMA

---

## Documentation for package `CARMA' version 5.0

## Help Pages

|  |  |
| --- | --- |
| CARMA | CARMA (Computational Analysis of Replicate Measures for Arrays) |
