# Supplementary material for: CARMA: A platform for analyzing microarray datasets that incorporate replicate measures
Source: BMC Bioinformatics. 2006 Mar 17;7:149. doi: 10.1186/1471-2105-7-149 (PMC1450302; doi:10.1186/1471-2105-7-149)
Supplement: Additional File 2 — CARMA_5.0.zip An R package for installing CARMA under the Microsoft Windows operating system. [file 1471-2105-7-149-S2.zip › CARMA/html/CARMA.html]

R: CARMA (Computational Analysis of Replicate Measures for Arrays)

|  |  |
| --- | --- |
| CARMA {CARMA} | R Documentation |

## CARMA (Computational Analysis of Replicate Measures for Arrays)

### Description

CARMA is a microarray analysis platform that reads data files generated by
most microarray image processing software packages, performs ANOVA using a
user-defined linear model, and produces easily interpretable graphical and
numeric results. No pre-processing of the data is required and user-specified
parameters control most aspects of the analysis including statistical
significance criterion. The software also performs location and intensity
dependent lowess normalization, automatic outlier detection and removal, and
accommodates missing data. In addition, multiple effects of interest can be
modeled, allowing studies that examine combinations of effects such as genotype
and treatment, dosage and time, or genotype and time.

### Usage

```
CARMA(pchrFileInput = "", pchrFileNormalized = "")
```

### Arguments

|  |  |
| --- | --- |
| `pchrFileInput` | the name and path of the Input.txt file. If this argument is left blank, a dialogue box will appear to allow the file to be selected. |
| `pchrFileNormalized` | the name and path of the Normalized.txt file. If this argument is left blank, a dialogue box will appear to allow the file to be selected. This argument is used to allow the analysis of variance (ANOVA) to be run on a dataset that has been previously processed by CARMA. This prevents the data from being re-read and re-normalized, and thus speeding up the analysis. |

### Value

No value is returned. Output files are created in the Output directory specified
in the Input.txt file.

### Note

Please refer to http://www.u.arizona.edu/~jhoying/CARMA.html to obtain the
necessary files and instructions for an example analysis of a microarray dataset.

### Author(s)

Kevin Greer kgreer@u.arizona.edu and Jay Hoying jhoying@u.arizona.edu

### References

http://www.u.arizona.edu/~jhoying/CARMA.html

### Examples

```
 CARMA()
```

---

[Package *CARMA* version 5.0 Index]
