# Supplementary material for: CARMA: A platform for analyzing microarray datasets that incorporate replicate measures
Source: BMC Bioinformatics. 2006 Mar 17;7:149. doi: 10.1186/1471-2105-7-149 (PMC1450302; doi:10.1186/1471-2105-7-149)
Supplement: Additional File 4 — CARMAAquaporin1.zip The configuration files used to process the aquaporin-1 example dataset using CARMA. [file 1471-2105-7-149-S4.zip › Microarray/Aquaporin1/Output/Plot.pdf]

2 − UNKNOWN

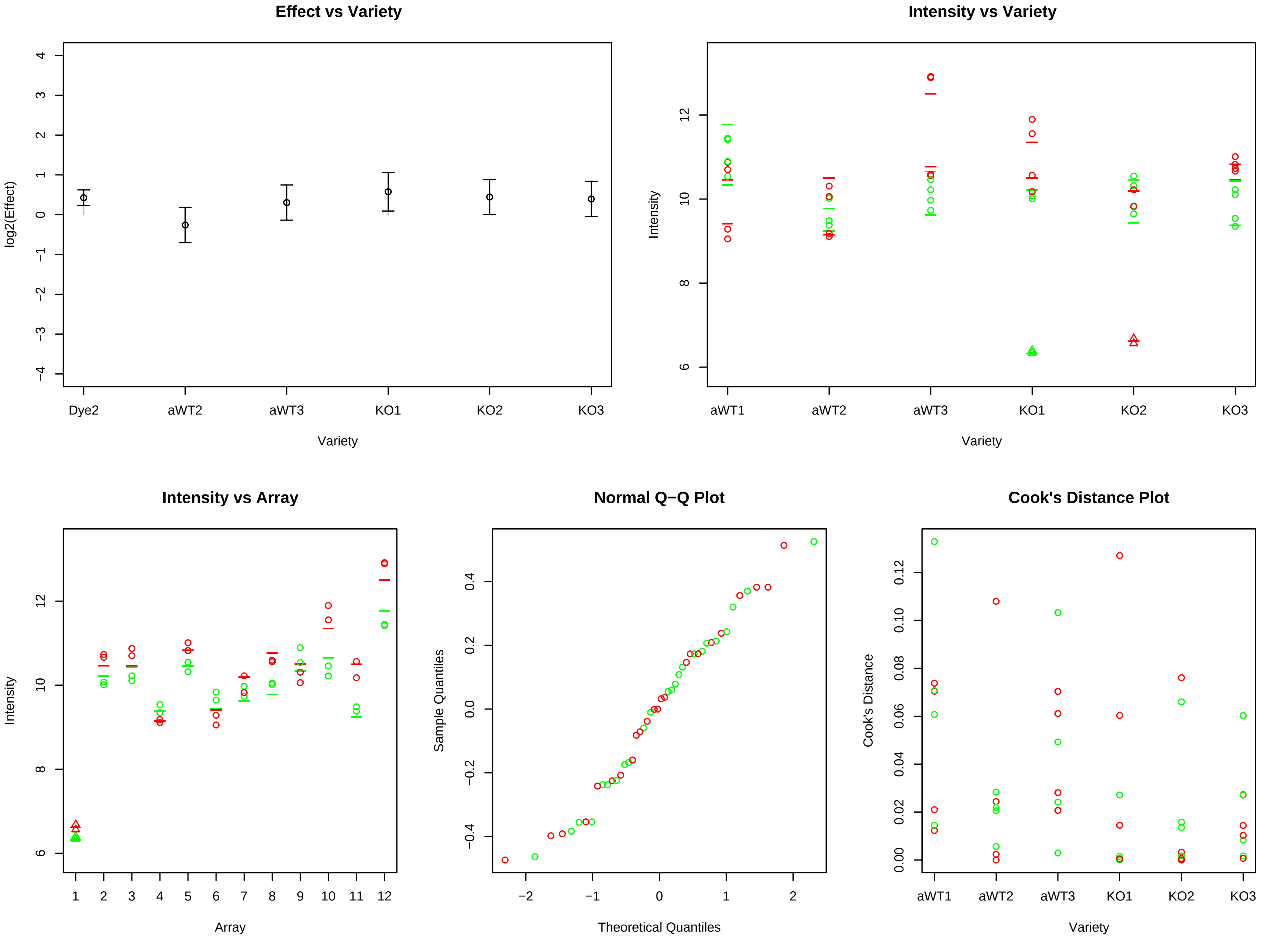

Flagged as: 101

Va = 0.5378

Probability > 0.05.

4 − 1200006L06RIK PROTEIN (FRAGMENT) homolog [Mus musculus]

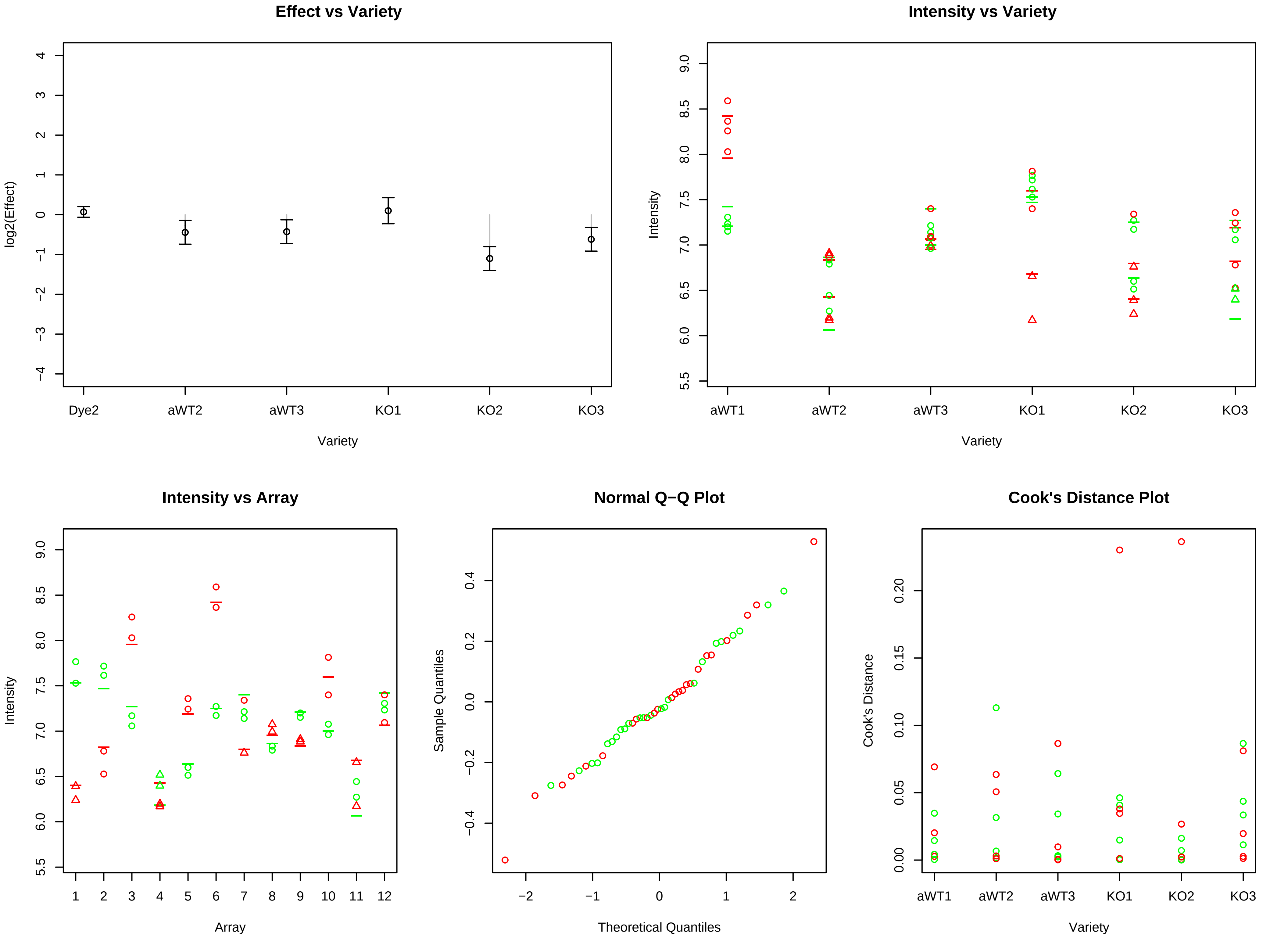

Flagged as: 111

Va = 0.03965

7 − Mus musculus RIKEN cDNA 1210001E11 gene (1210001E11Rik), mRNA

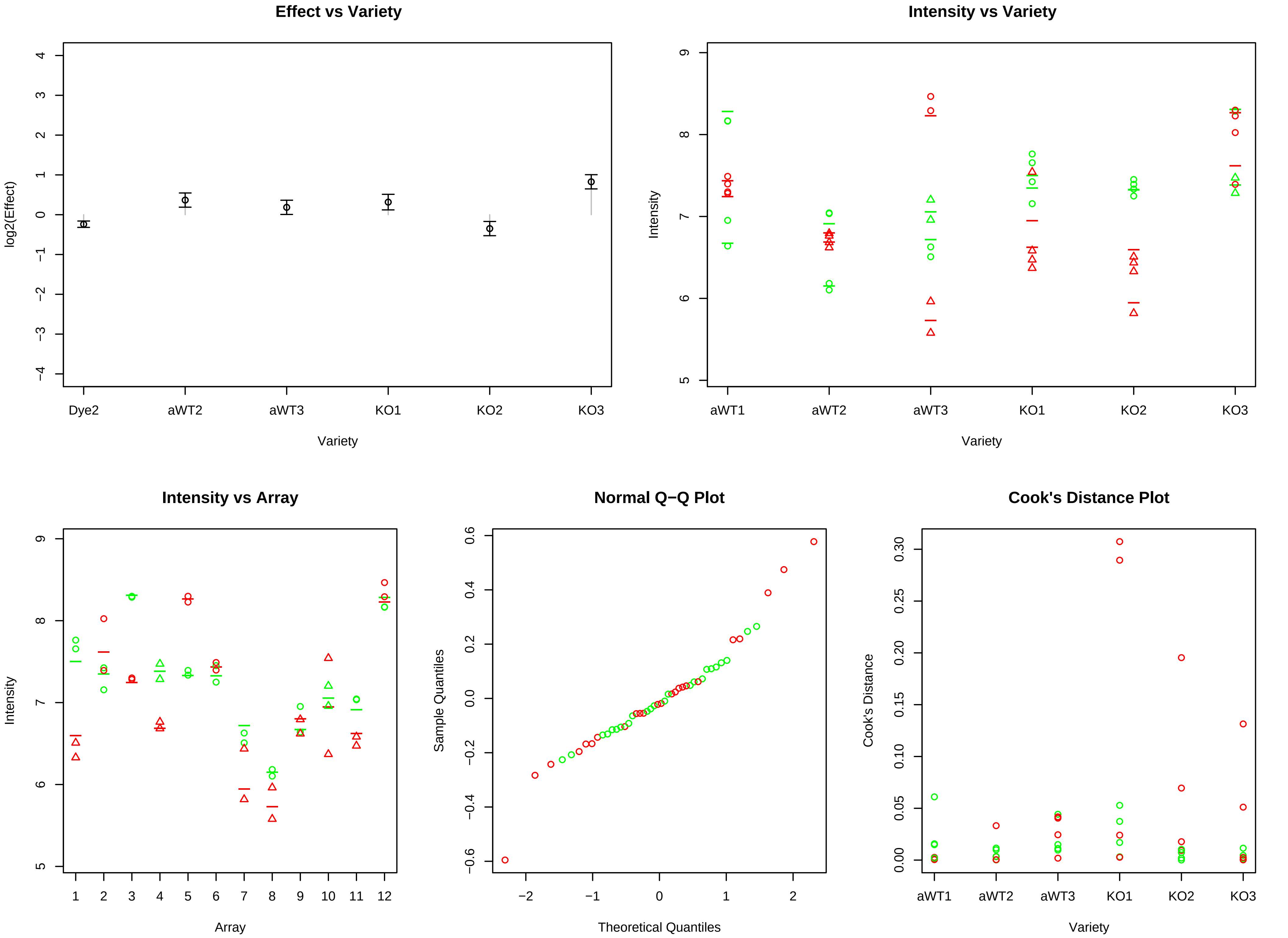

Flagged as: 111

Va = 0.008267

21 − Mus musculus gamma−aminobutyric acid (GABA−A) transporter 3 (Gabt3), mRNA

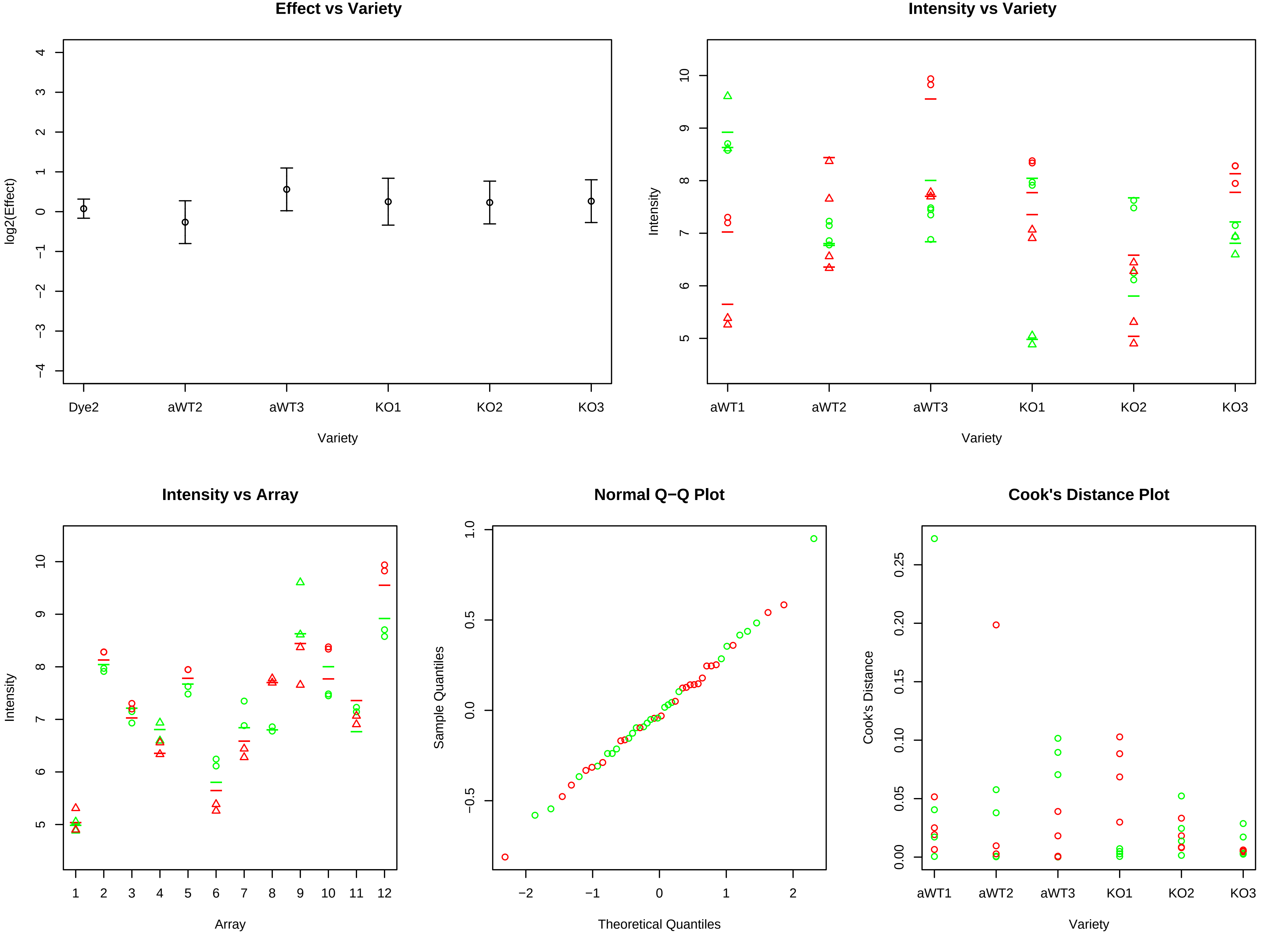

Flagged as: 101

Va = 0.7304

Probability > 0.05.

23 − Mus musculus Fc receptor, IgG, alpha chain transporter (Fcgrt), mRNA

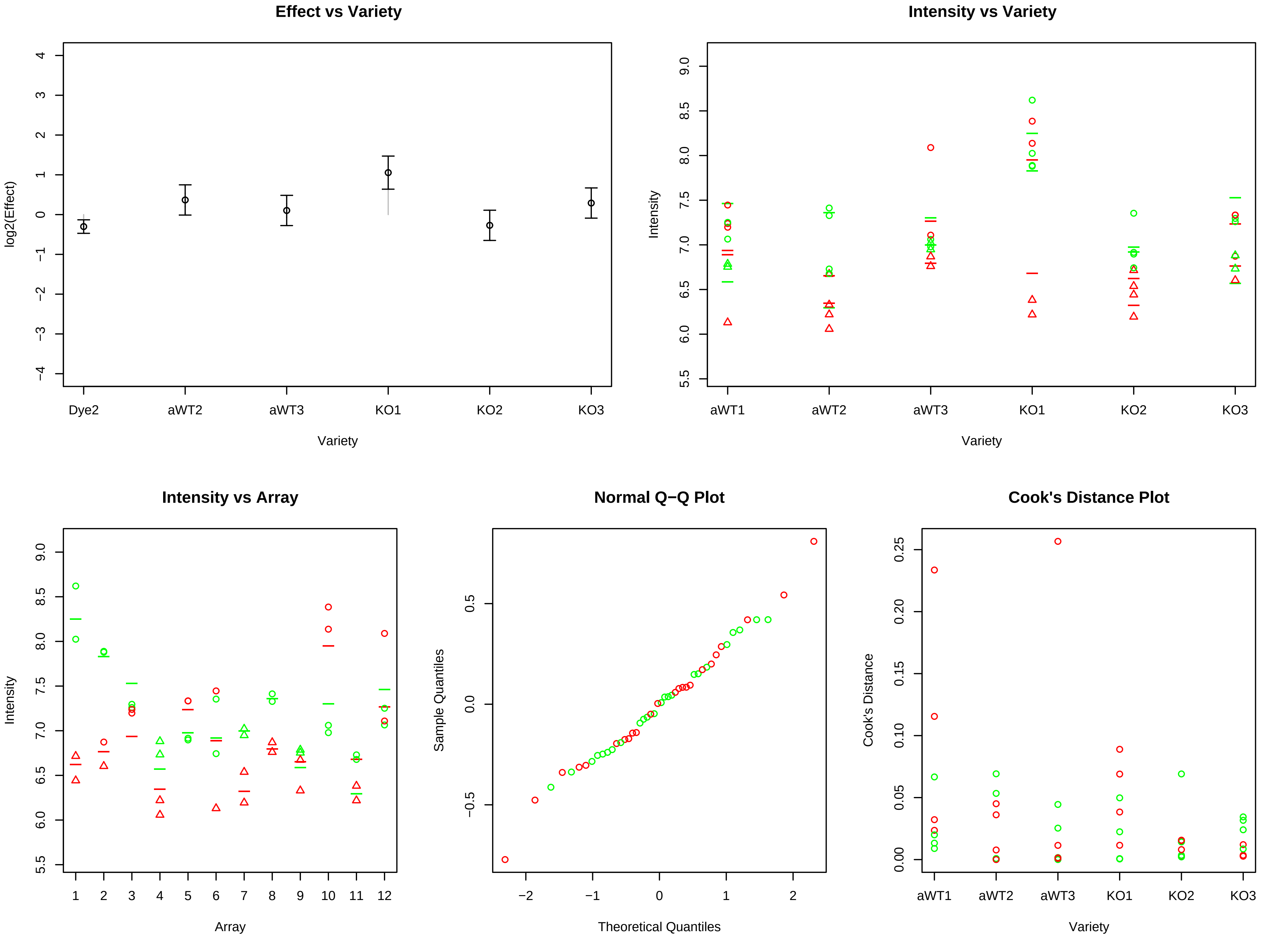

Flagged as: 101

Va = 0.1357

Probability > 0.05.

24 − Mus musculus Vhlh−interacting deubiquitinating enzyme 1 (Vdu1−pending), mRNA

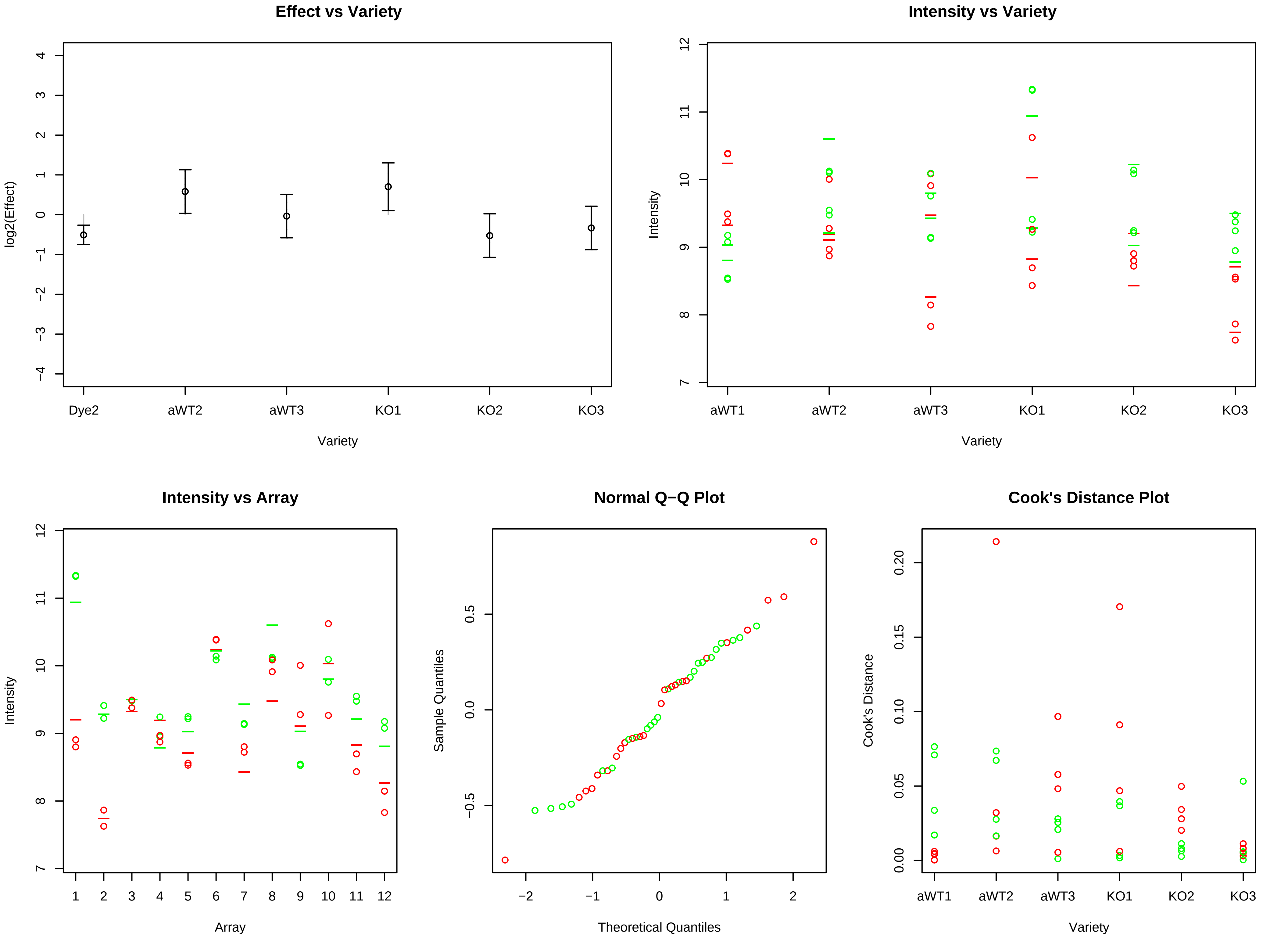

Flagged as: 101

Va = 0.3171

Probability > 0.05.

26 − Mus musculus insulin−like growth factor binding protein 3 (Igfbp3), mRNA

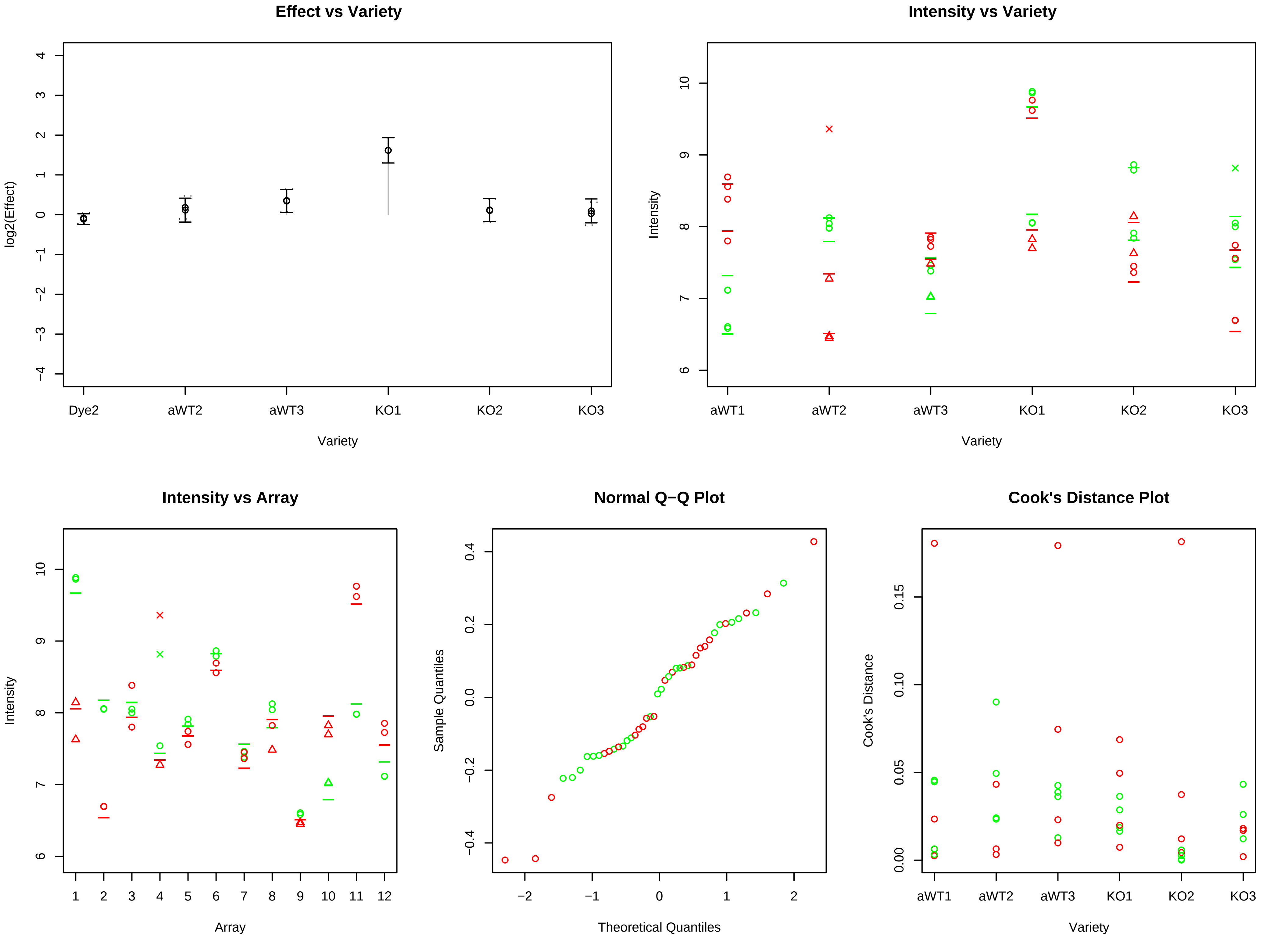

Flagged as: 111

Va = 0.01046

32 − Mus musculus secretory carrier membrane protein 2 (Scamp2), mRNA

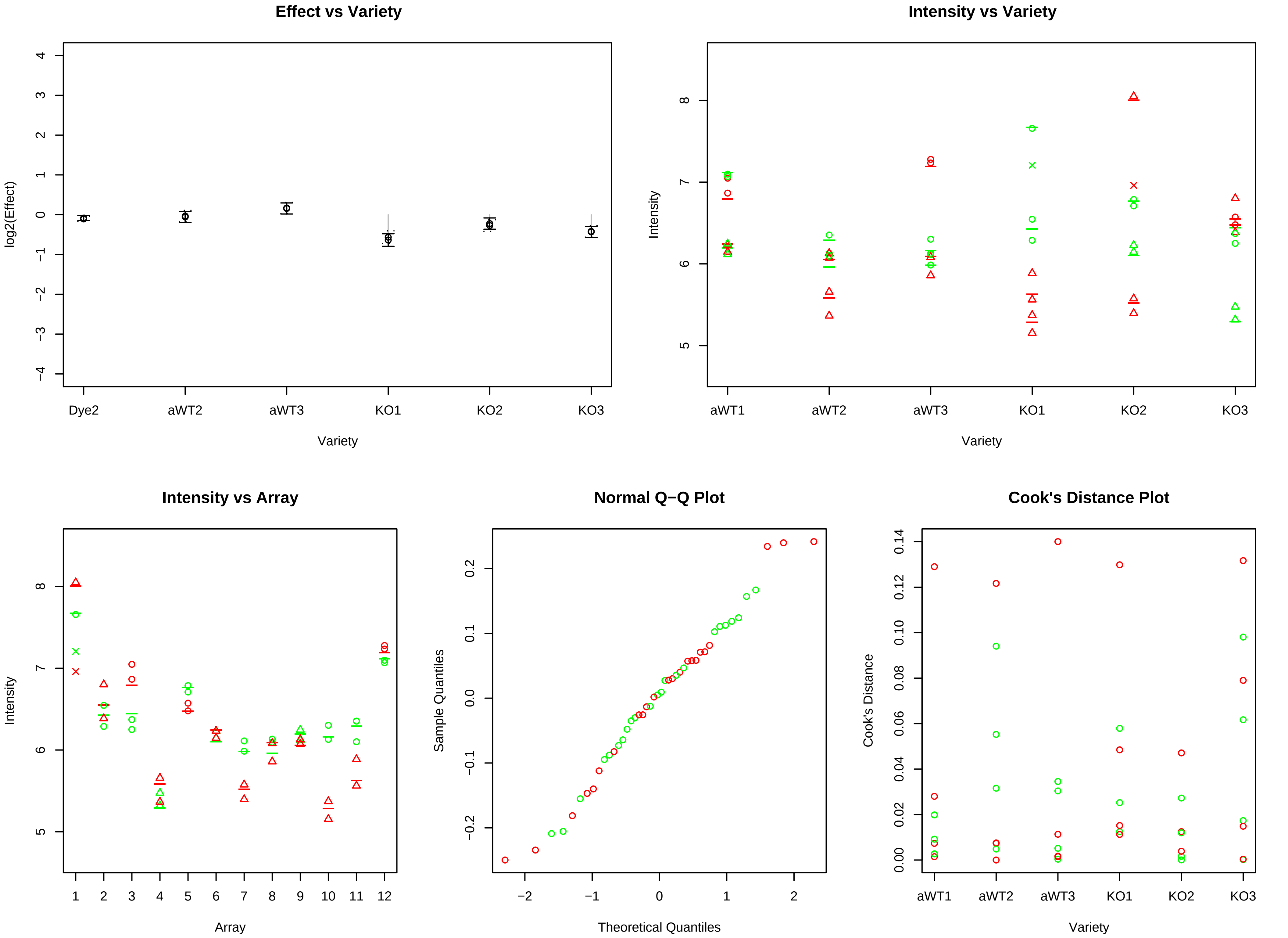

Flagged as: 111

Va = 0.01476

47 − Mus musculus vacuolar protein sorting 45 (yeast) (Vps45), mRNA

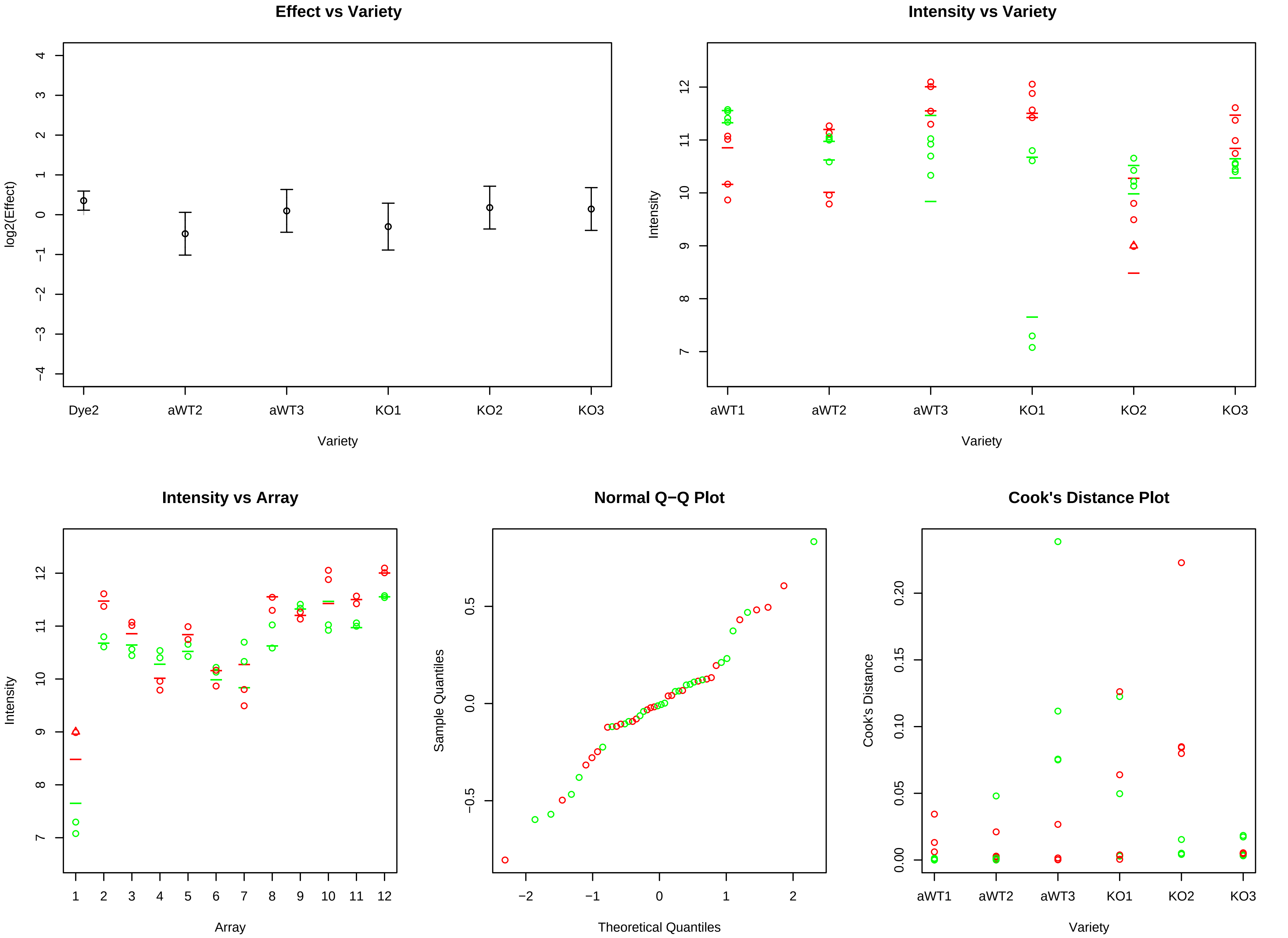

Flagged as: 101

Va = 0.788

Probability > 0.05.

48 − Mus musculus vacuolar protein sorting 26 (yeast) (Vps26), mRNA

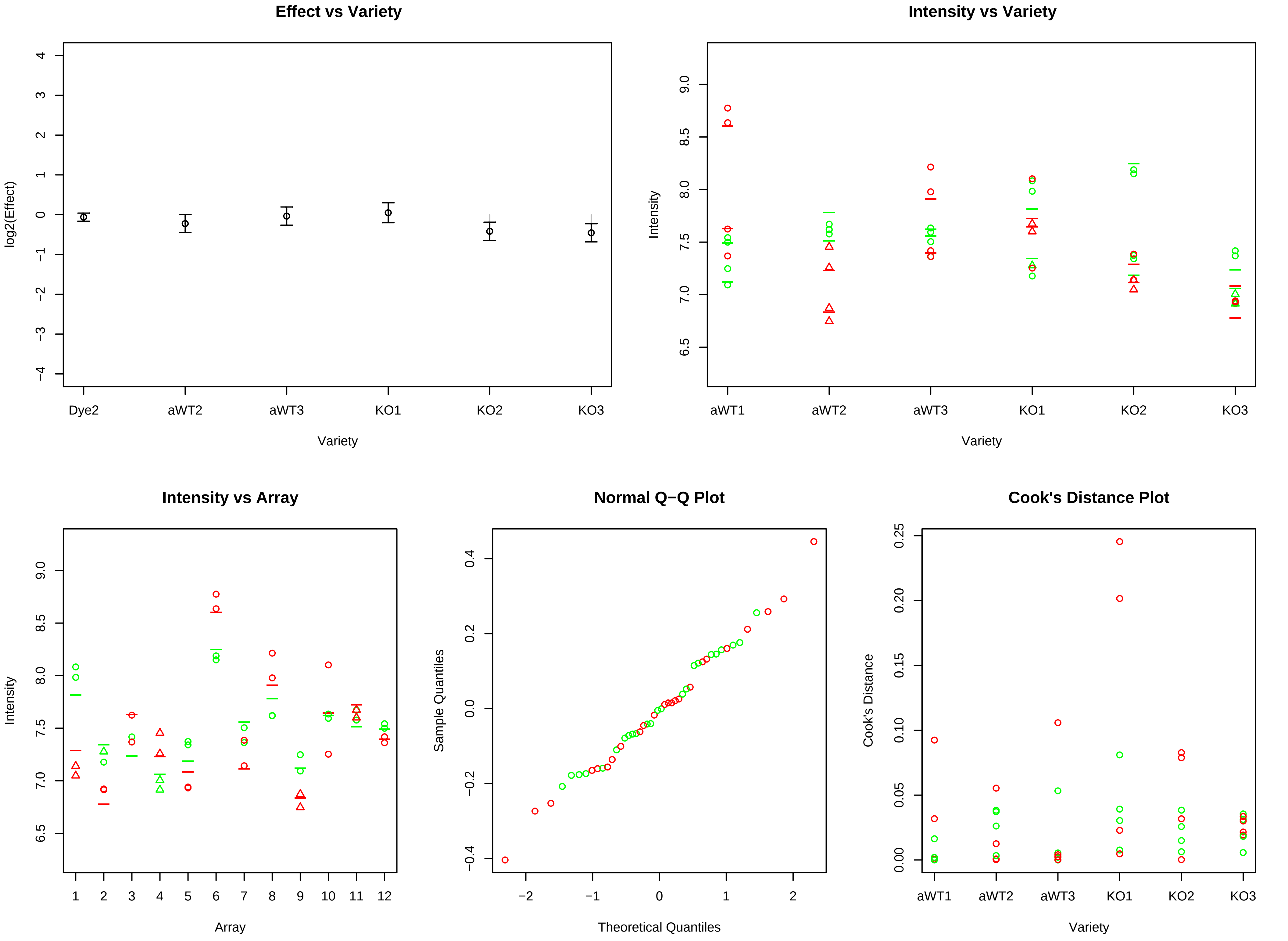

Flagged as: 100

Va = 0.2208

Probability > 0.05. Effect < 0.585.

49 − H3139G01

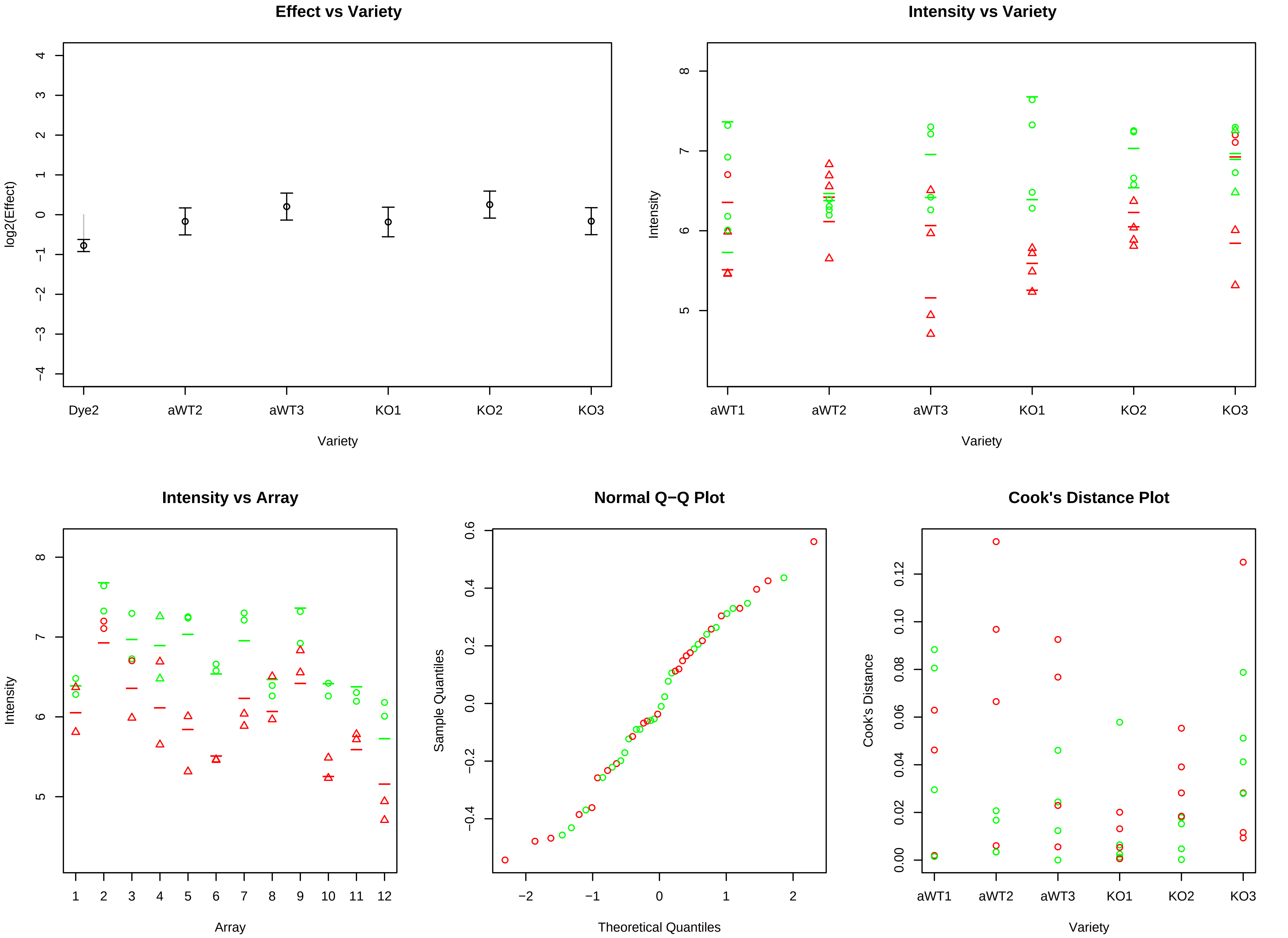

Flagged as: 100

Va = 0.7093

Probability > 0.05. Effect < 0.585.

50 − H3139G07

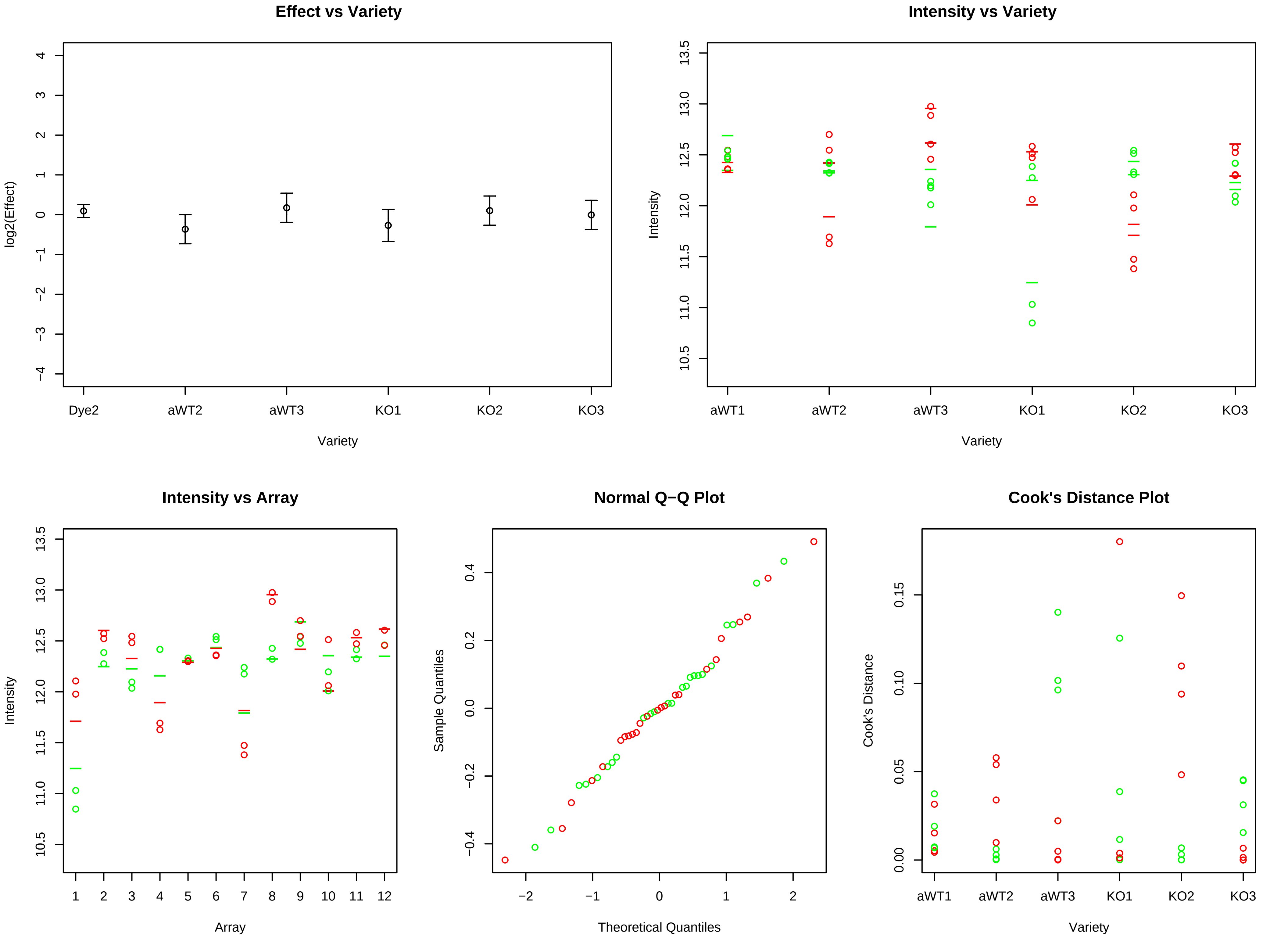

Flagged as: 100

Va = 0.6847

Probability > 0.05. Effect < 0.585.

51 − Mus musculus timeless homolog (Drosophila) (Timeless), mRNA

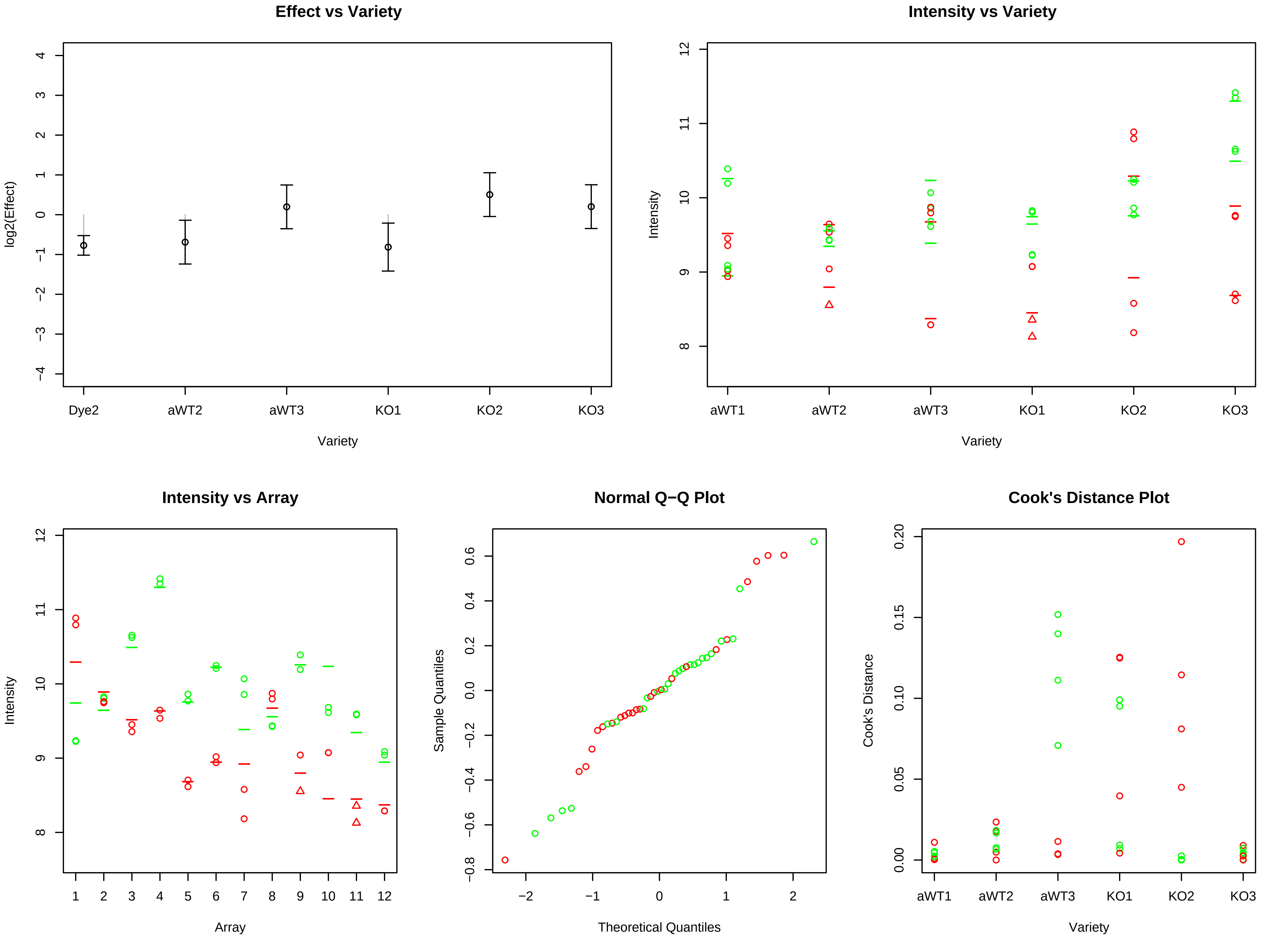

Flagged as: 101

Va = 0.2551

Probability > 0.05.

53 − UNKNOWN

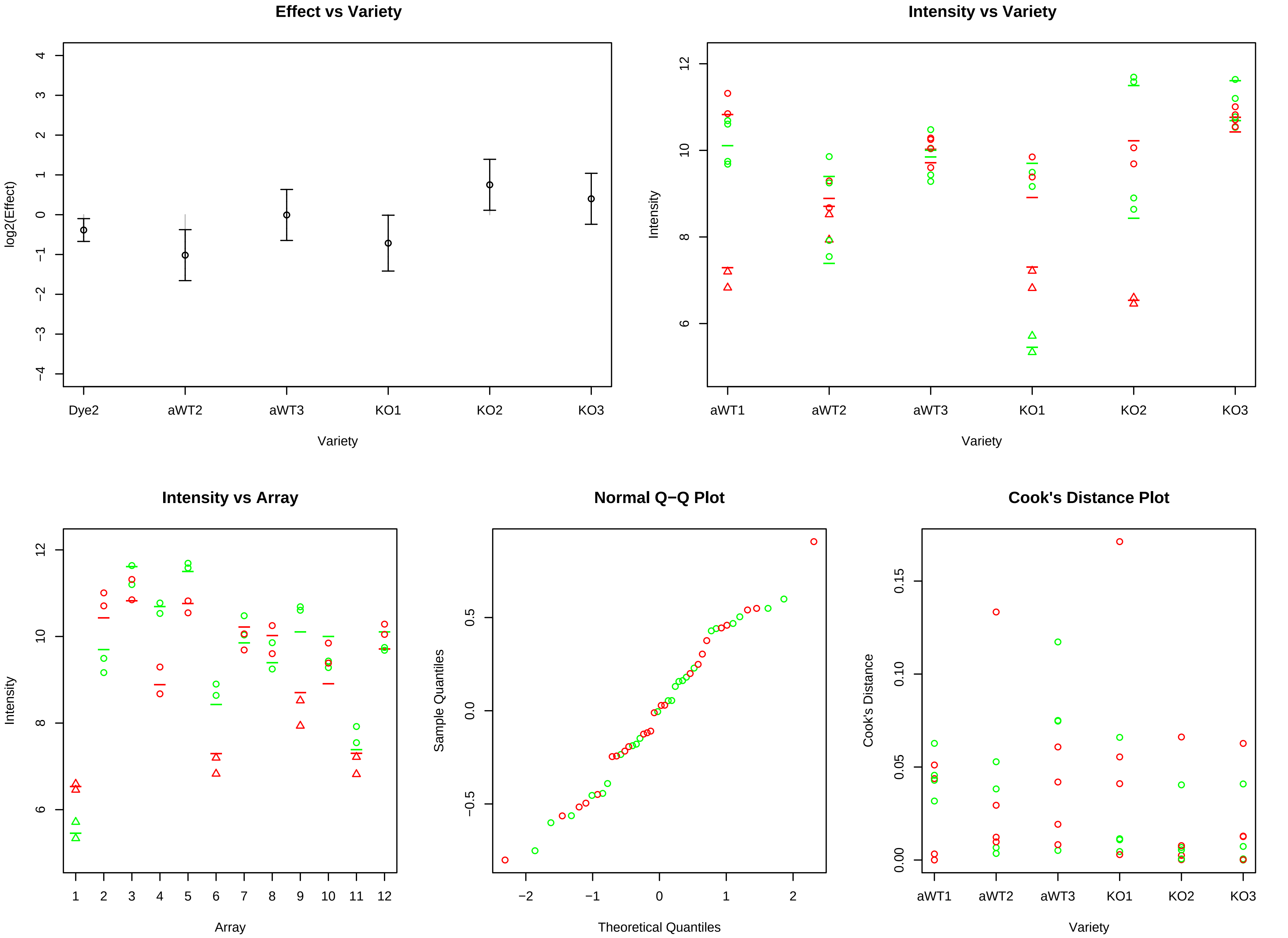

Flagged as: 101

Va = 0.2247

Probability > 0.05.

55 − UNKNOWN

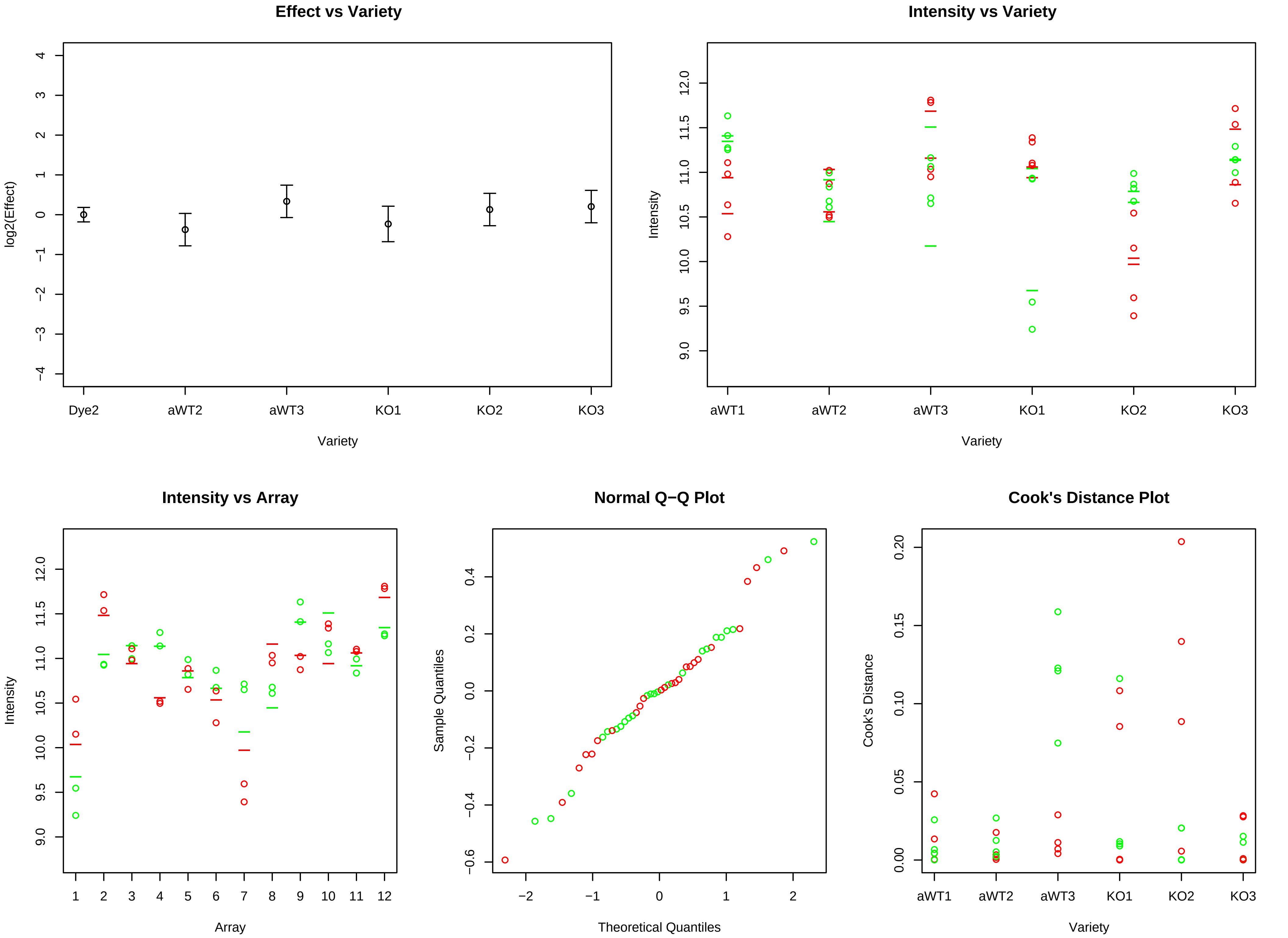

Flagged as: 101

Va = 0.5181

Probability > 0.05.

57 − TRIPARTITE MOTIF PROTEIN TRIM33 (FRAGMENT).

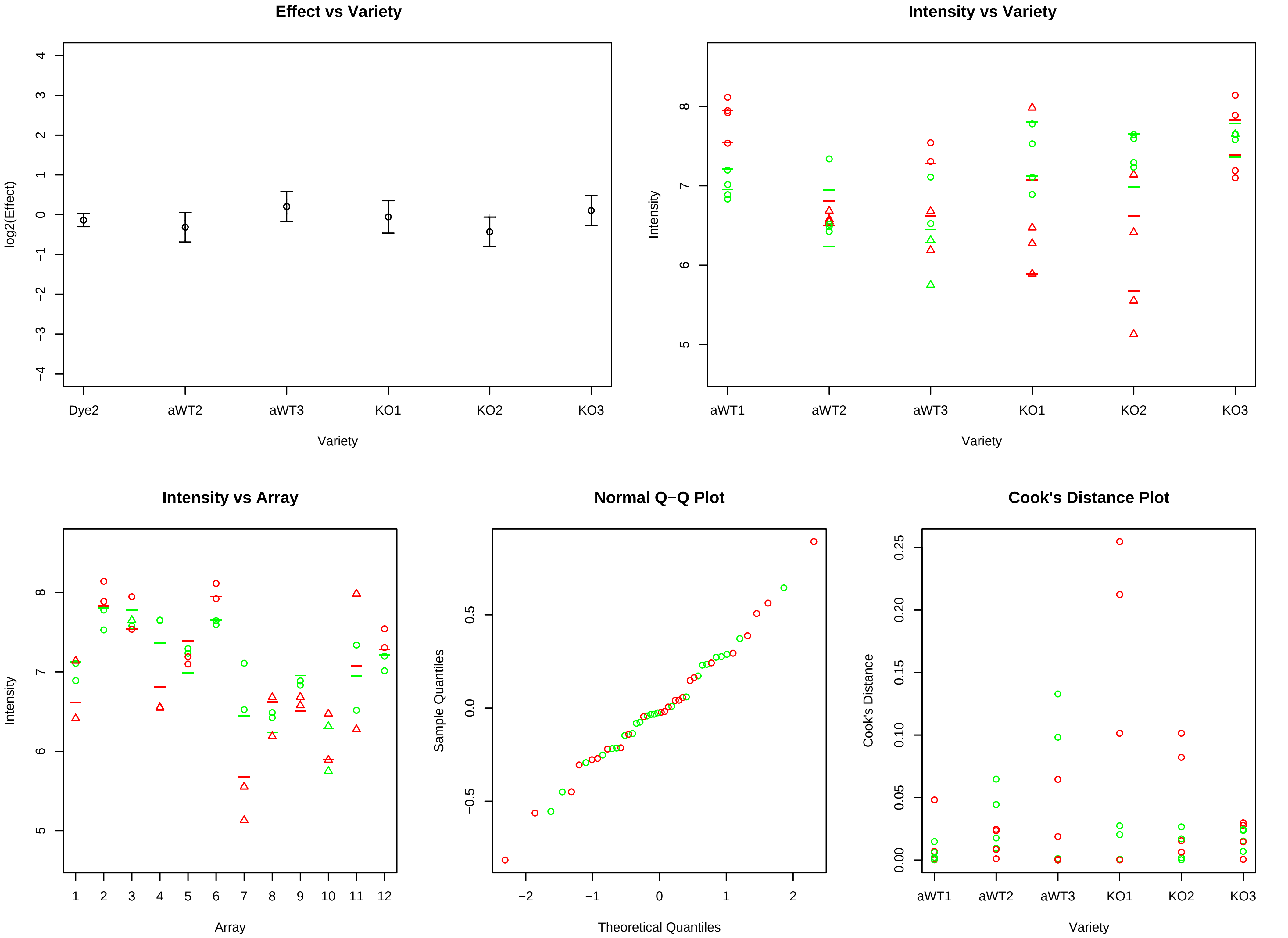

Flagged as: 101

Va = 0.4579

Probability > 0.05.

65 − Mus musculus oxidation resistance 1 (Oxr1), mRNA

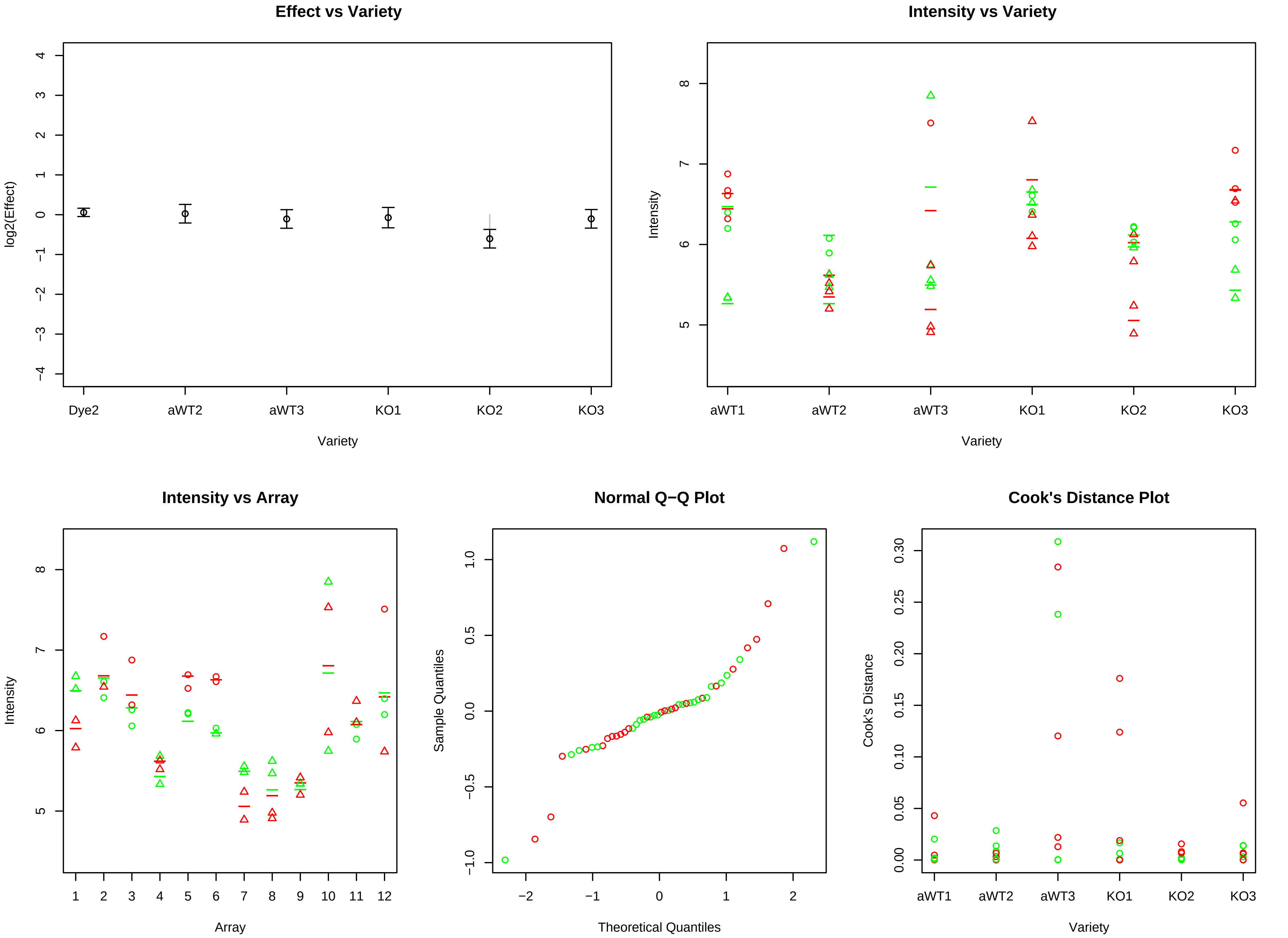

Flagged as: 101

Va = 0.2372

Probability > 0.05.

67 − Mus musculus sterol carrier protein 2, pseudogene 2 (Scp2−ps2)

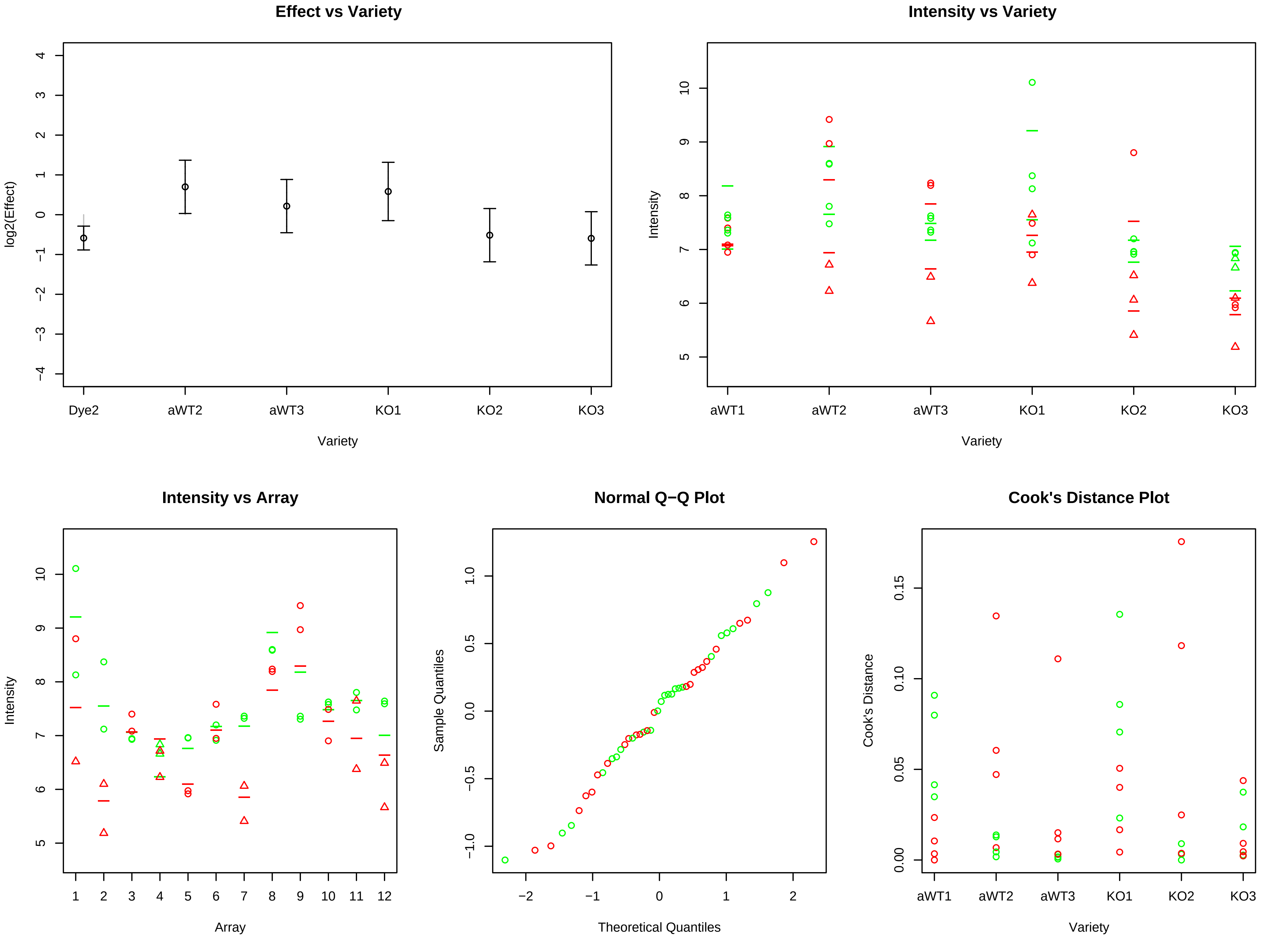

Flagged as: 101

Va = 0.4162

Probability > 0.05.

69 − UNKNOWN: Similar to Mus musculus RIKEN cDNA 2210407J23 gene (2210407J23Rik), mRNA

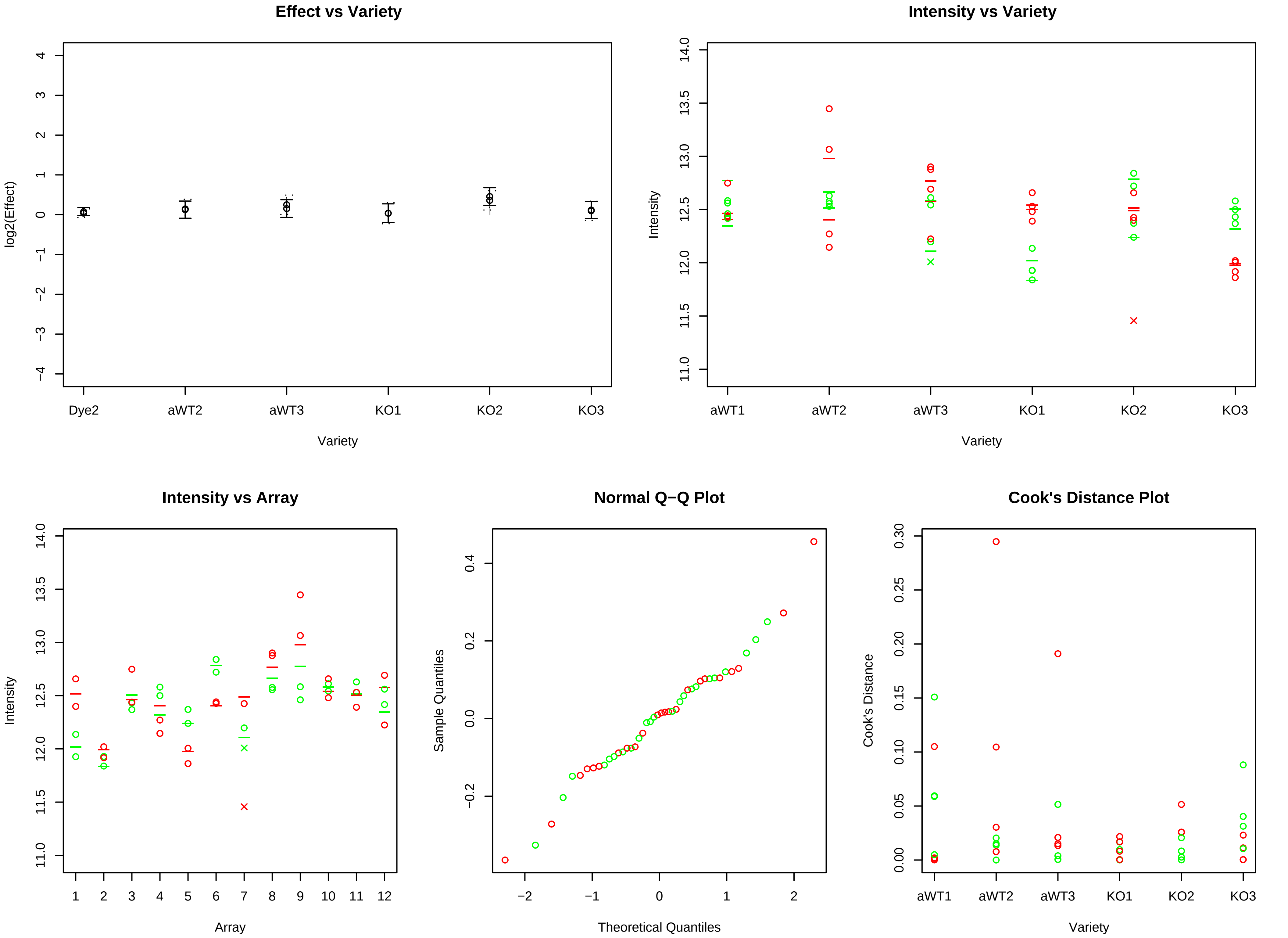

Flagged as: 100

Va = 0.4375

Probability > 0.05. Effect < 0.585.

71 − UNKNOWN

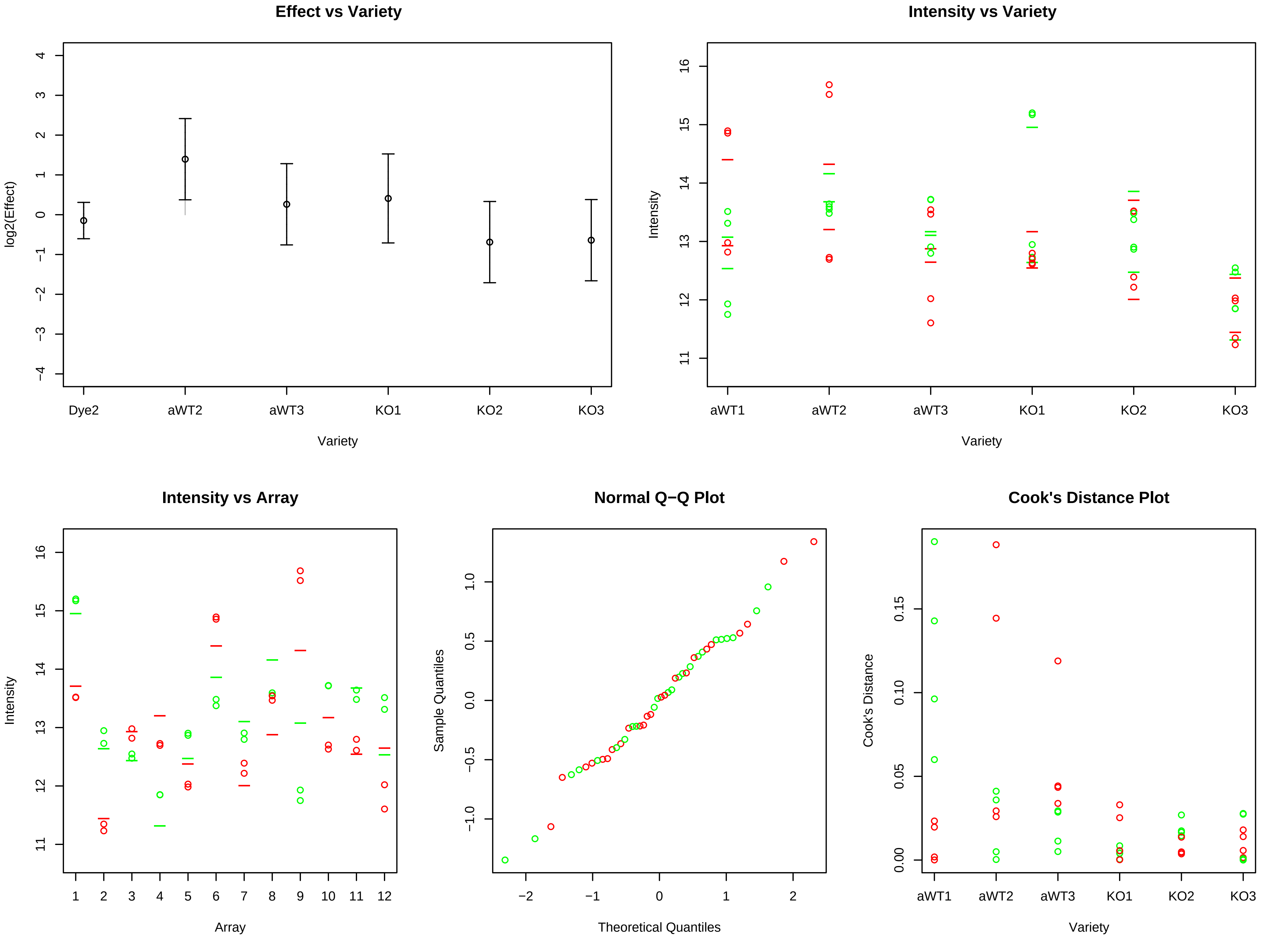

Flagged as: 101

Va = 0.4885

Probability > 0.05.

80 − Mus musculus transmembrane 4 superfamily member 6 (Tm4sf6), mRNA

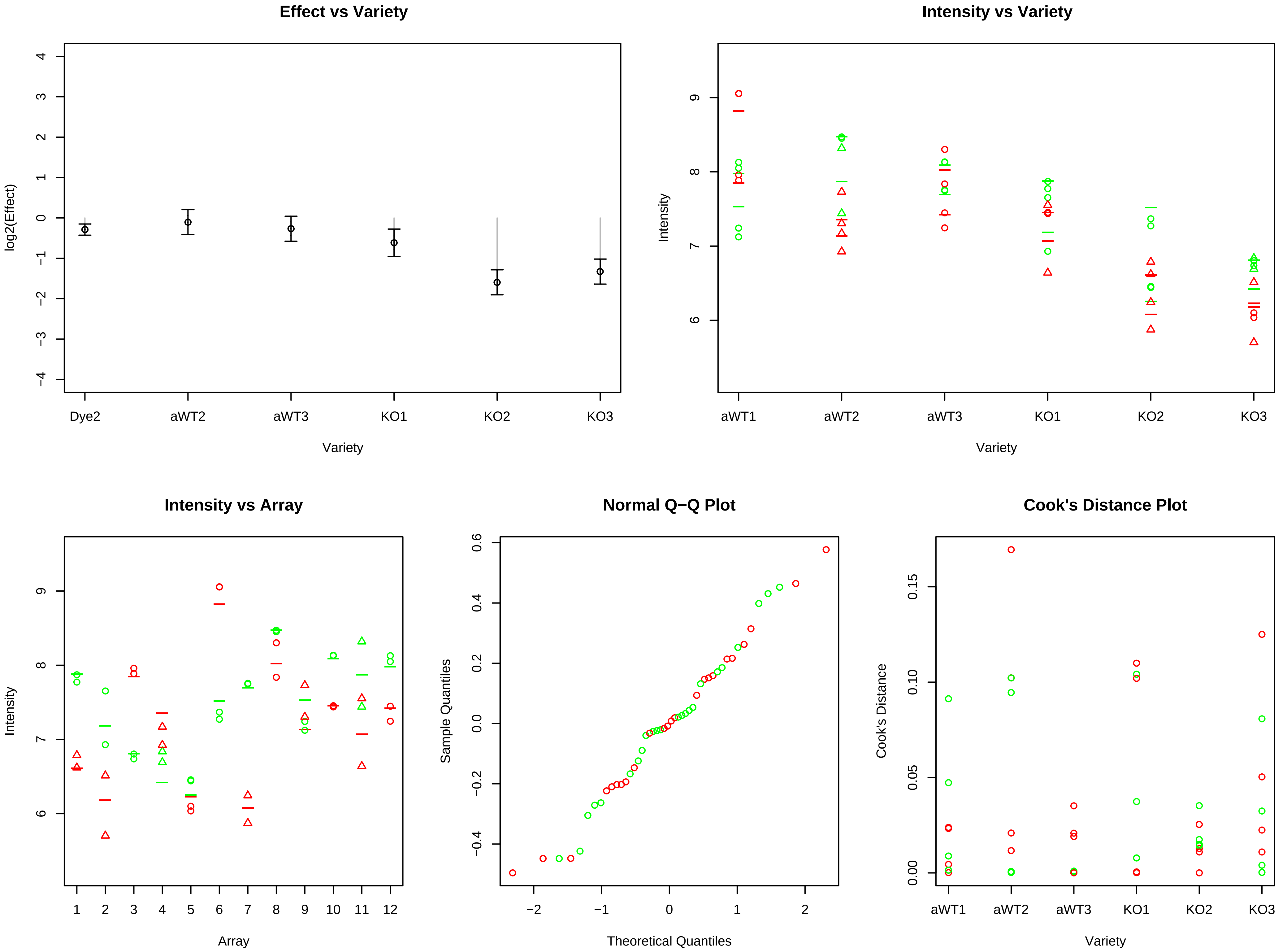

Flagged as: 111

Va = 0.01127

83 − H3118G01

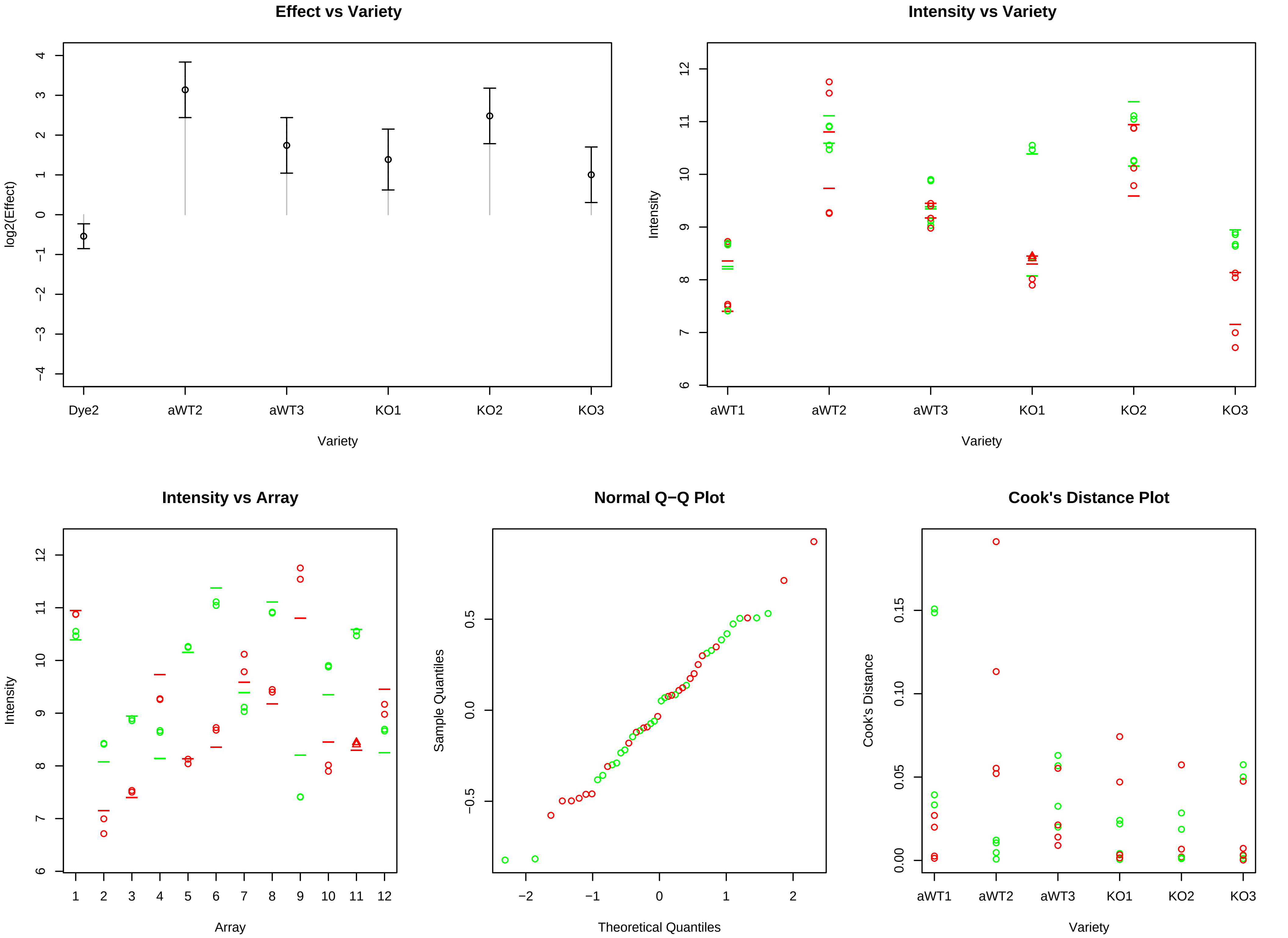

Flagged as: 111

Va = 0.02700

84 − Mus musculus eukaryotic translation elongation factor 1 beta 2 (Eef1b2), mRNA

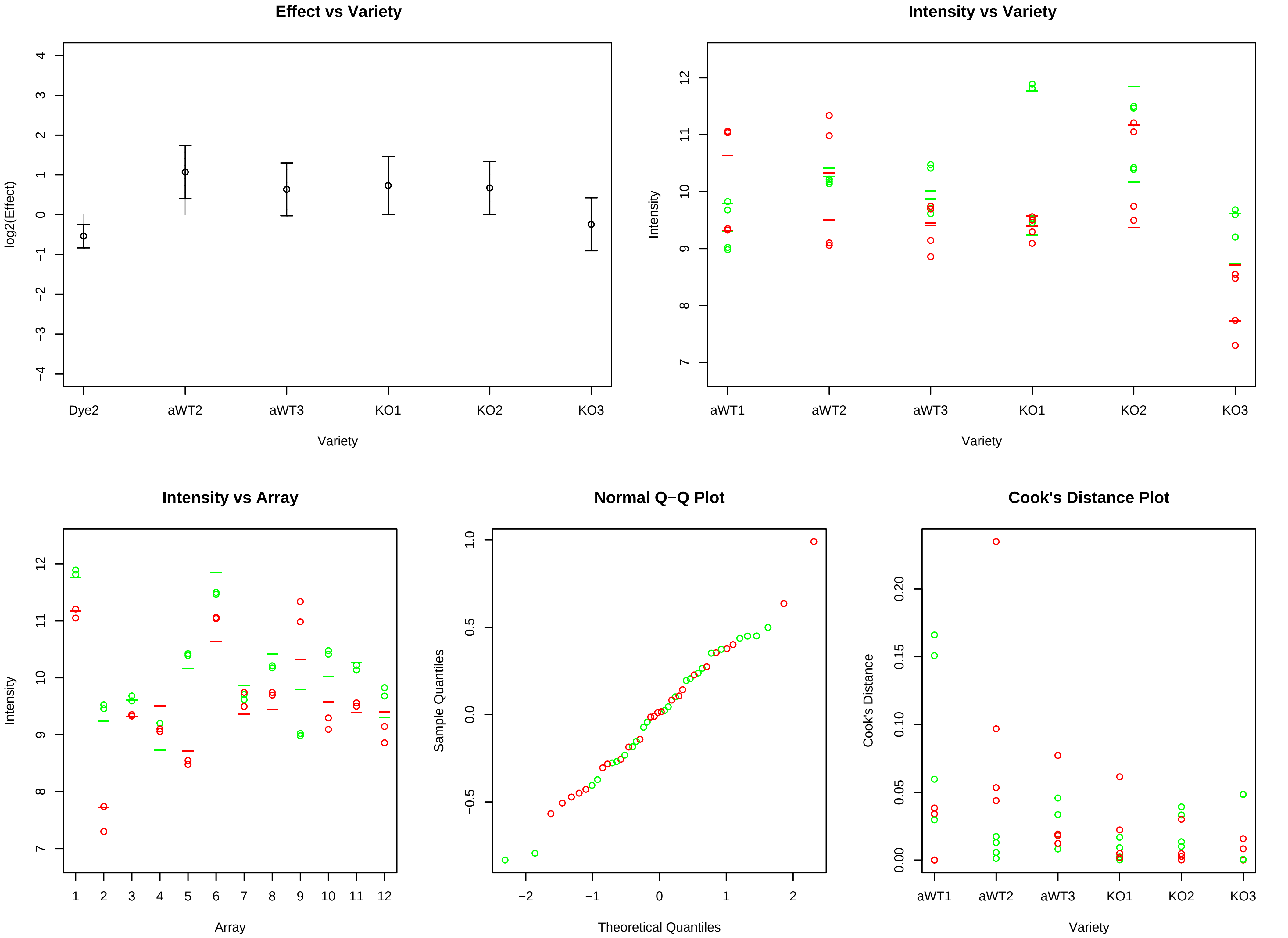

Flagged as: 101

Va = 0.4428

Probability > 0.05.

86 − Mus musculus RIKEN cDNA 2610027O18 gene (2610027O18Rik), mRNA

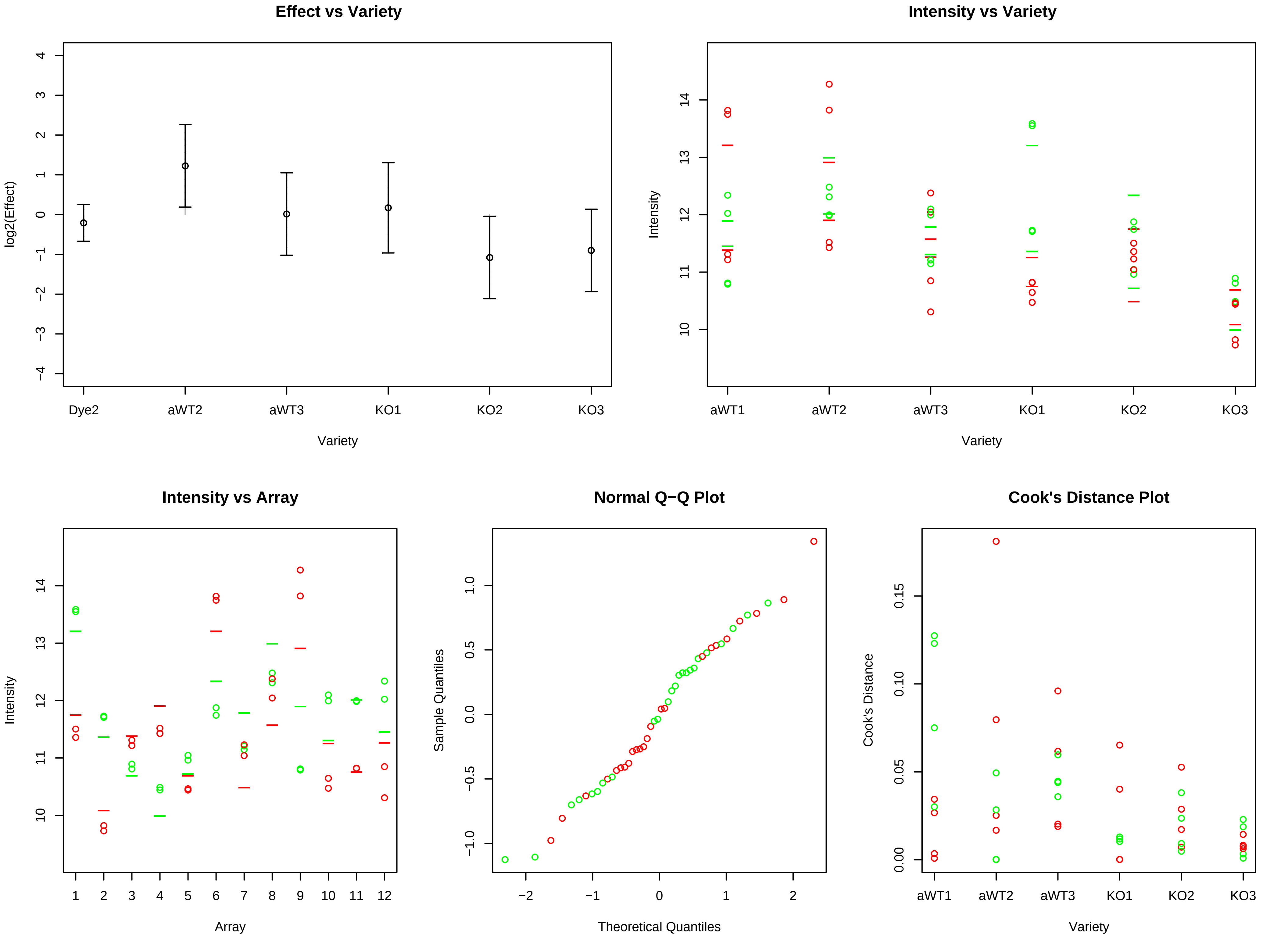

Flagged as: 101

Va = 0.4359

Probability > 0.05.

89 − Mus musculus RIKEN cDNA 0610007P06 gene (0610007P06Rik), mRNA

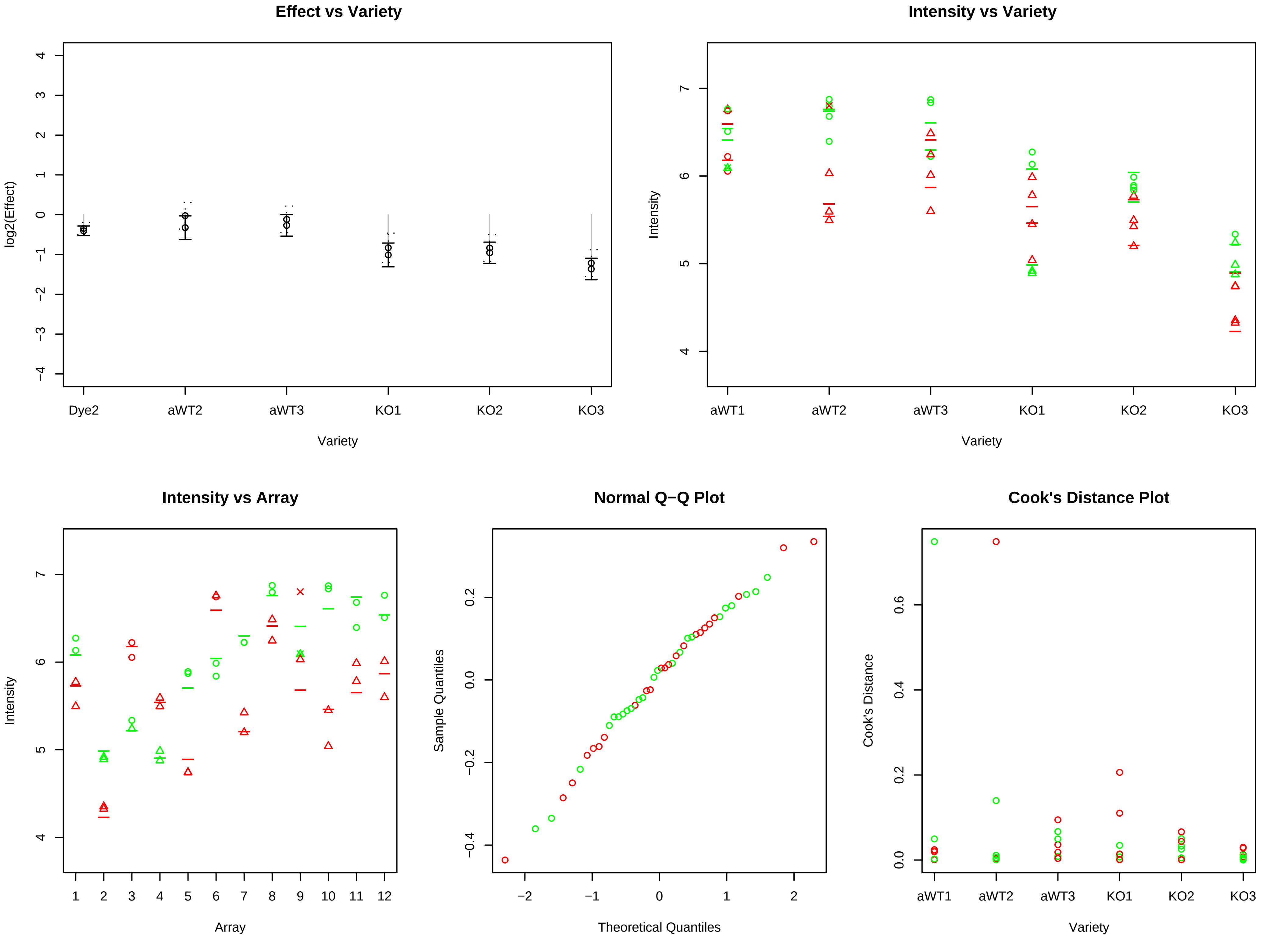

Flagged as: 111

Va = 0.01719

90 − Mus musculus RIKEN cDNA 5830475I06 gene (5830475I06Rik), mRNA

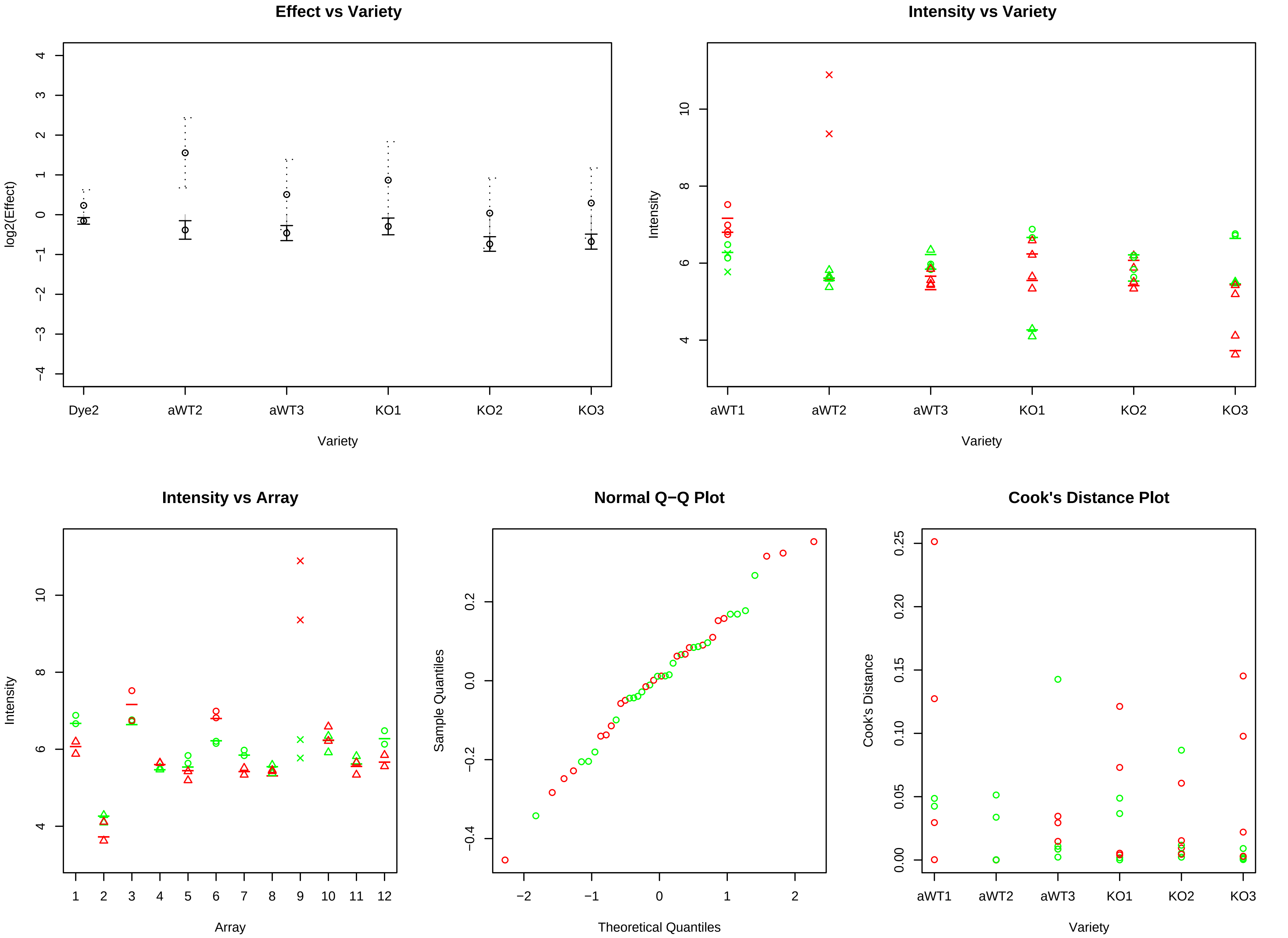

Flagged as: 101

Va = 0.05207

Probability > 0.05.

98 − Mus musculus RIKEN cDNA 3830408D24 gene (3830408D24Rik), mRNA

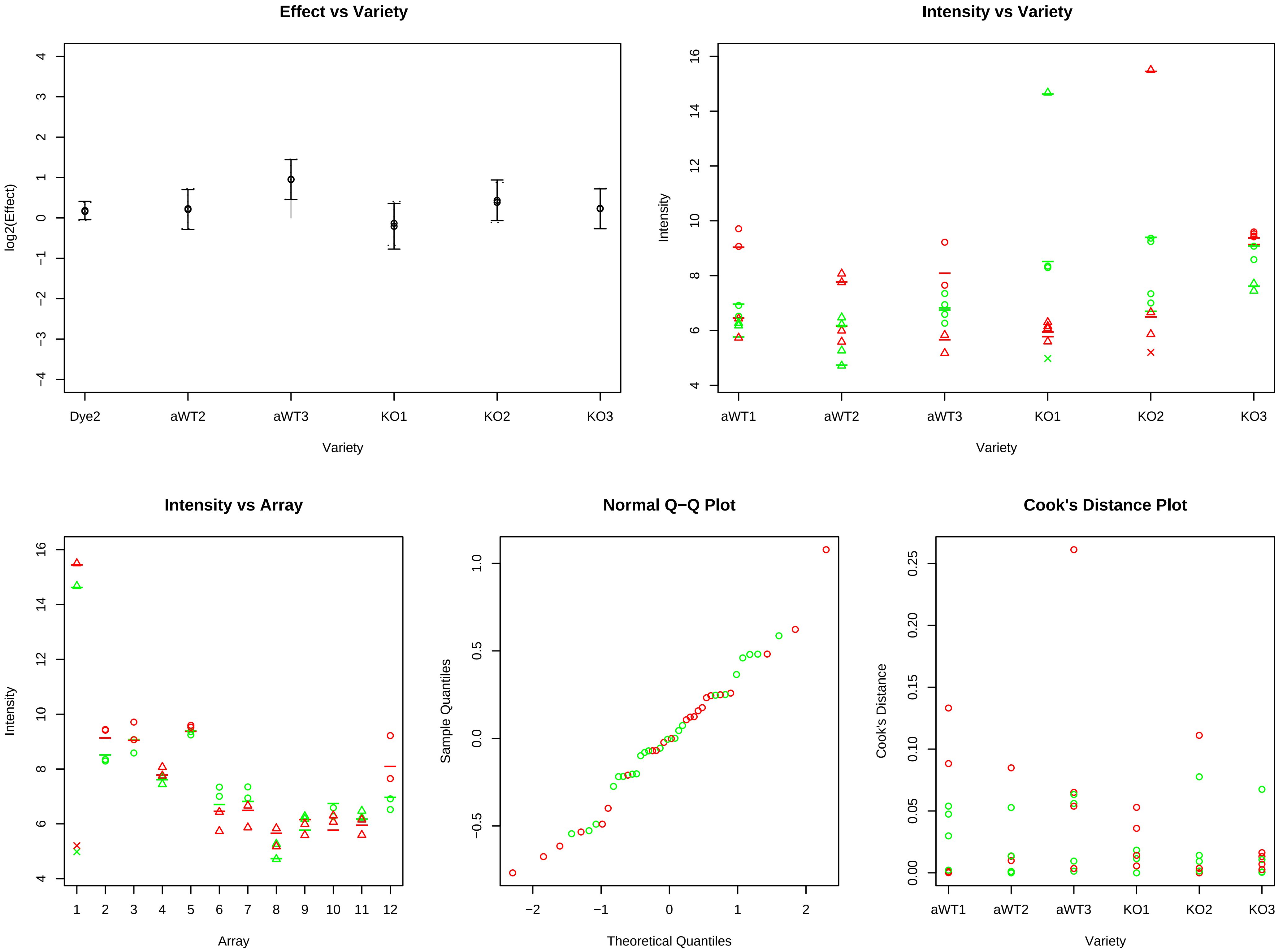

Flagged as: 101

Va = 0.3604

Probability > 0.05.

99 − Mus musculus ribosomal protein S5 (Rps5), mRNA

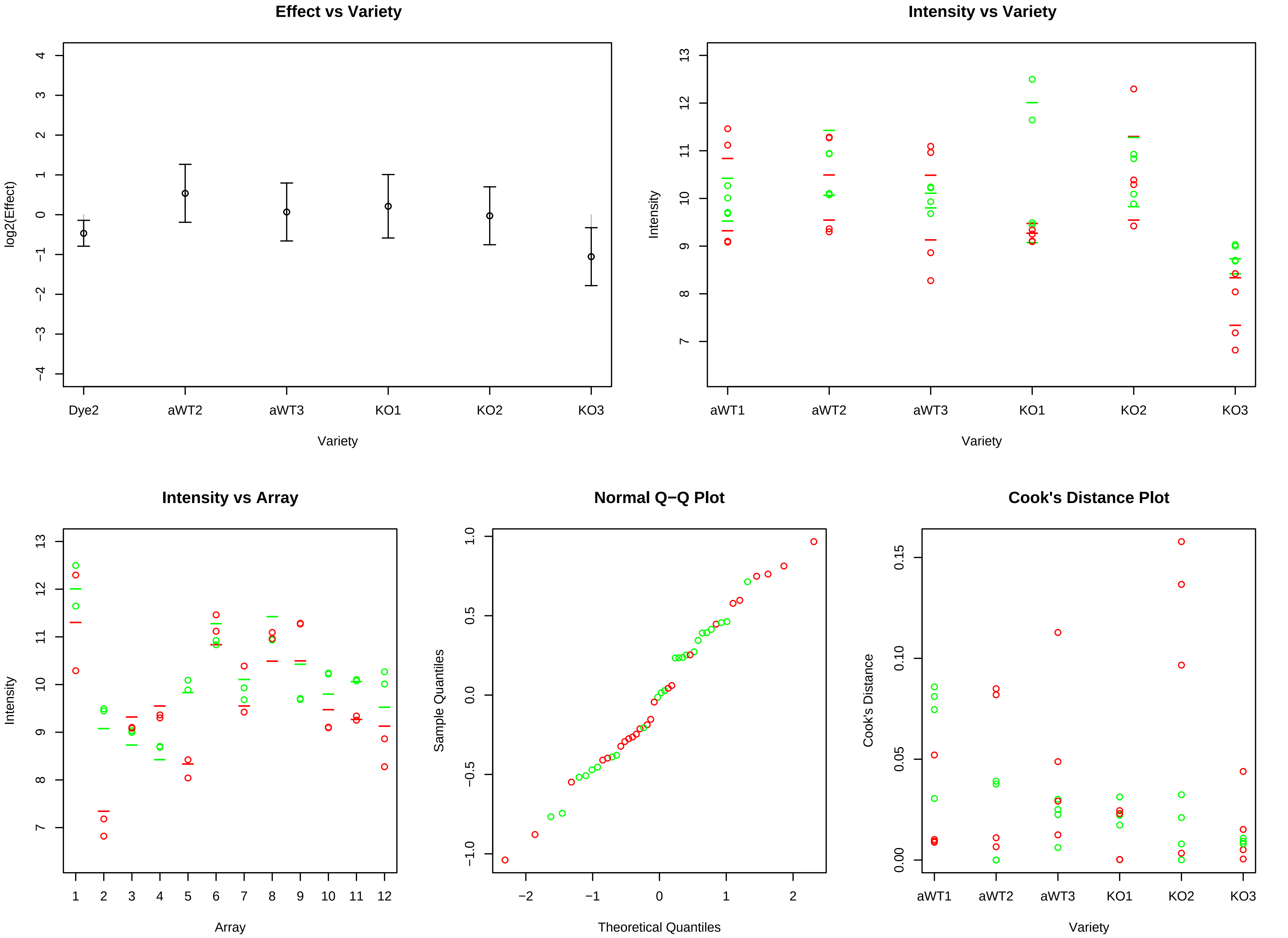

Flagged as: 101

Va = 0.4459

Probability > 0.05.
