# Supplementary material for: CARMA: A platform for analyzing microarray datasets that incorporate replicate measures
Source: BMC Bioinformatics. 2006 Mar 17;7:149. doi: 10.1186/1471-2105-7-149 (PMC1450302; doi:10.1186/1471-2105-7-149)
Supplement: Additional File 4 — CARMAAquaporin1.zip The configuration files used to process the aquaporin-1 example dataset using CARMA. [file 1471-2105-7-149-S4.zip › Microarray/Aquaporin1/Output/PlotSelect.pdf]

4 − 1200006L06RIK PROTEIN (FRAGMENT) homolog [Mus musculus]

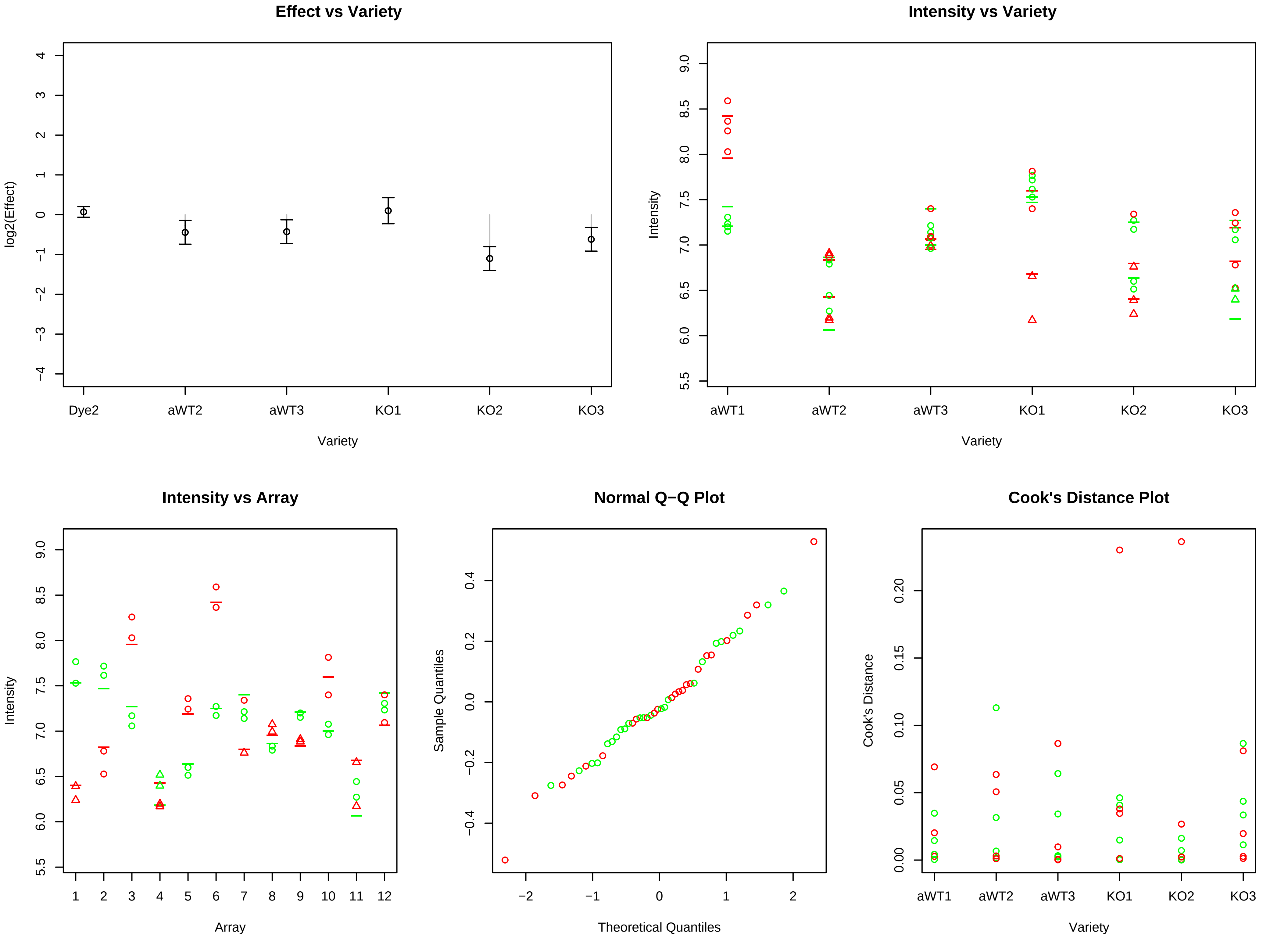

Flagged as: 111

Va = 0.03965

7 − Mus musculus RIKEN cDNA 1210001E11 gene (1210001E11Rik), mRNA

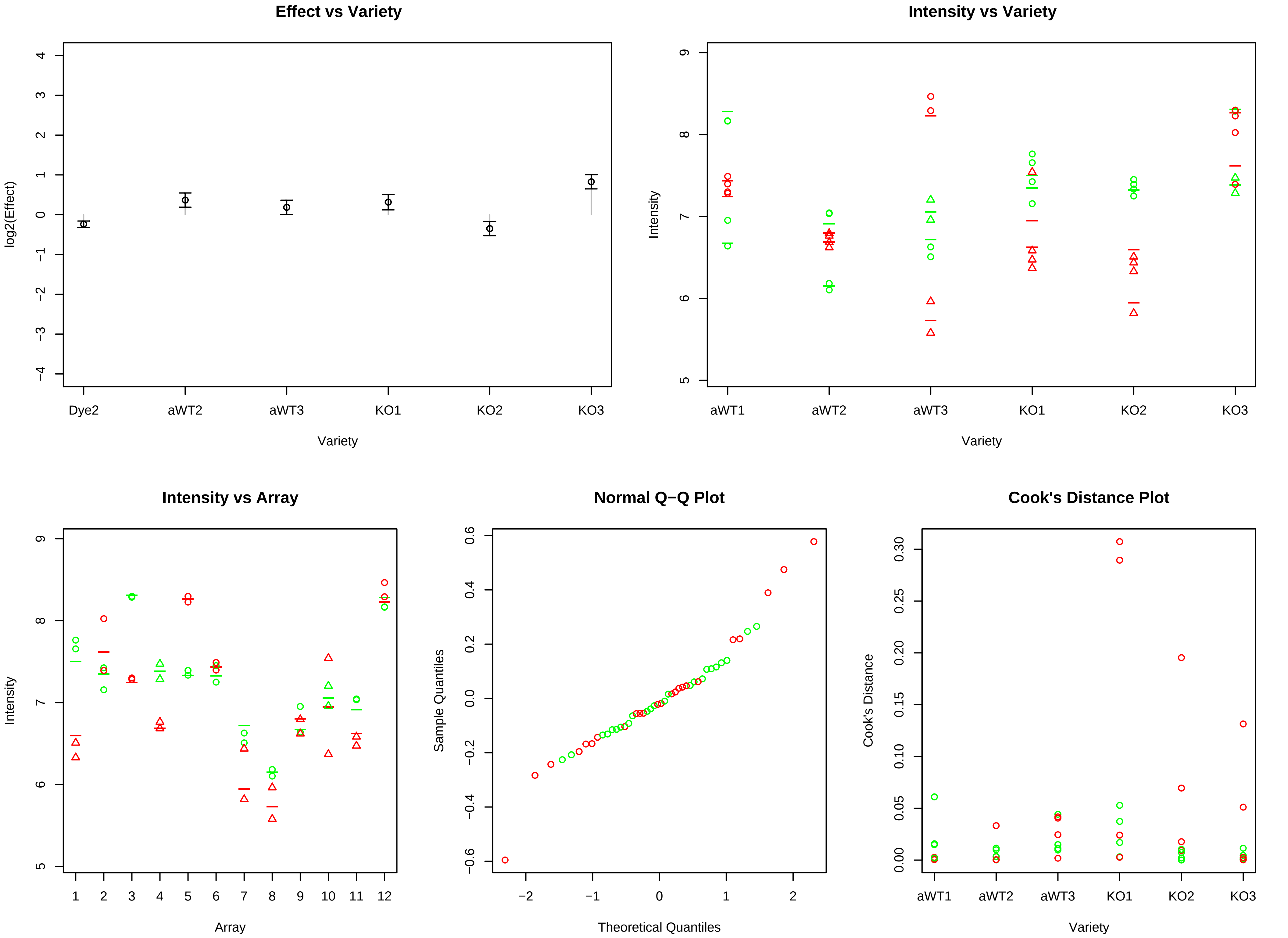

Flagged as: 111

Va = 0.008267

26 − Mus musculus insulin−like growth factor binding protein 3 (Igfbp3), mRNA

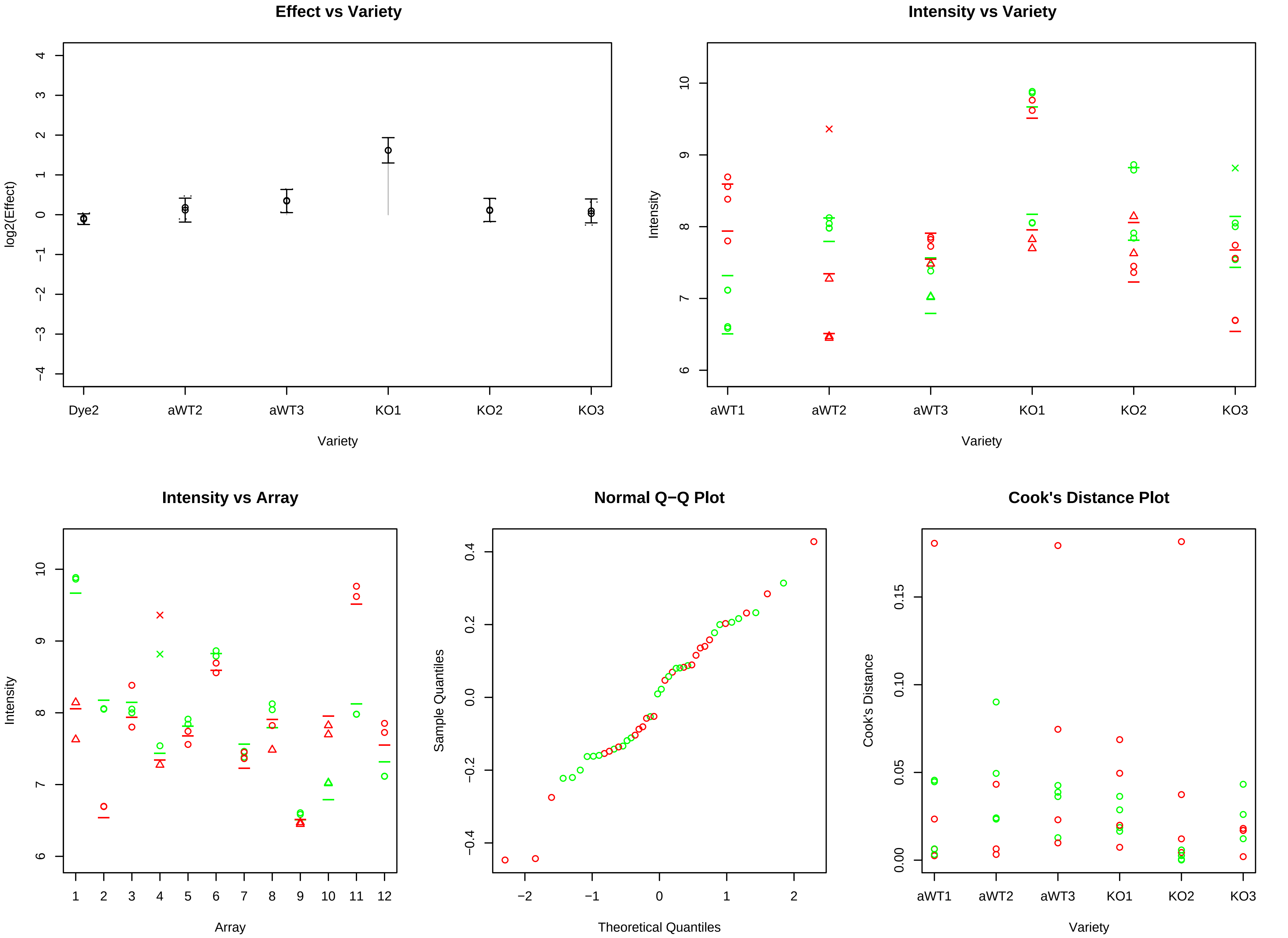

Flagged as: 111

Va = 0.01046

32 − Mus musculus secretory carrier membrane protein 2 (Scamp2), mRNA

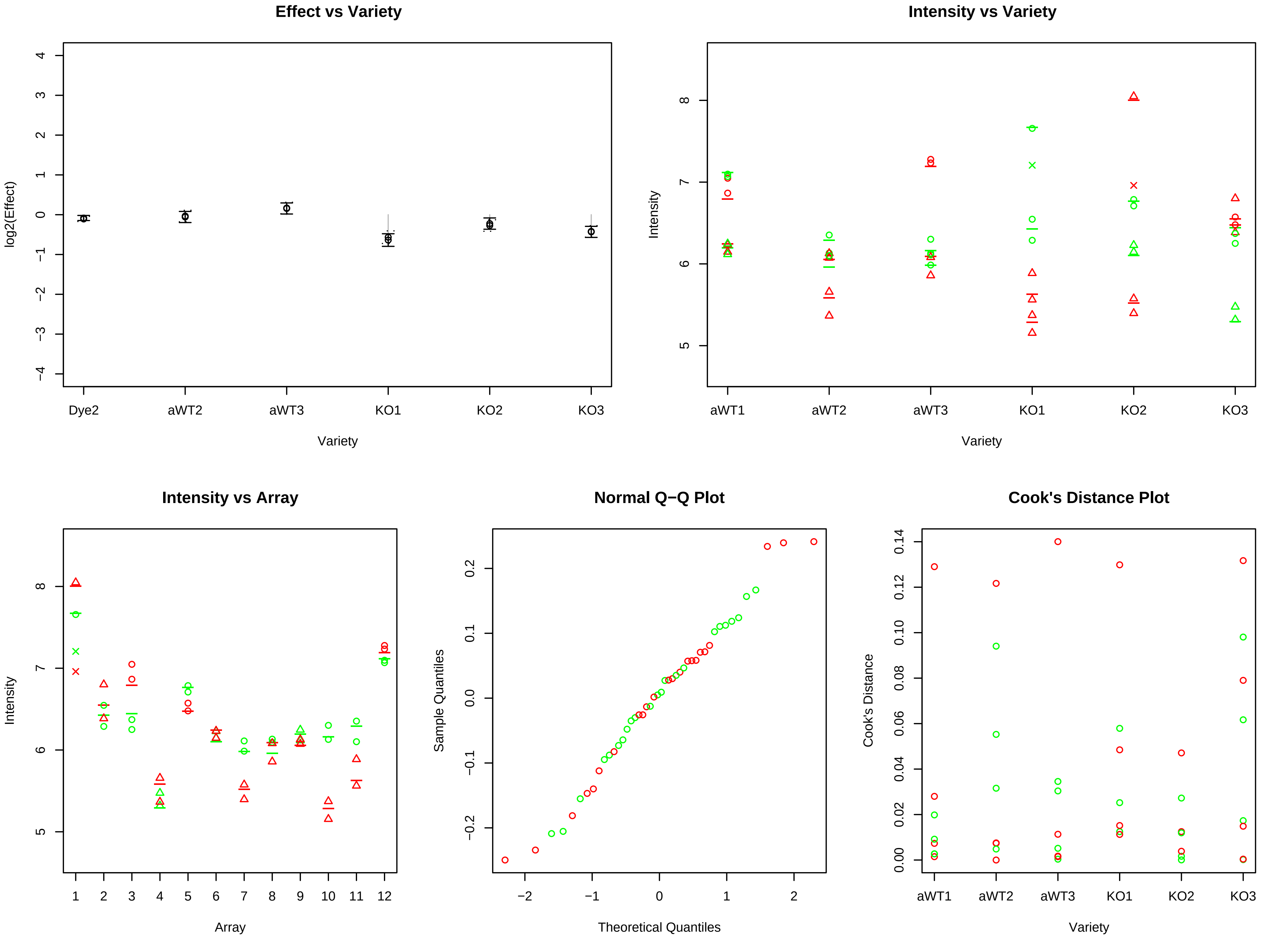

Flagged as: 111

Va = 0.01476

80 − Mus musculus transmembrane 4 superfamily member 6 (Tm4sf6), mRNA

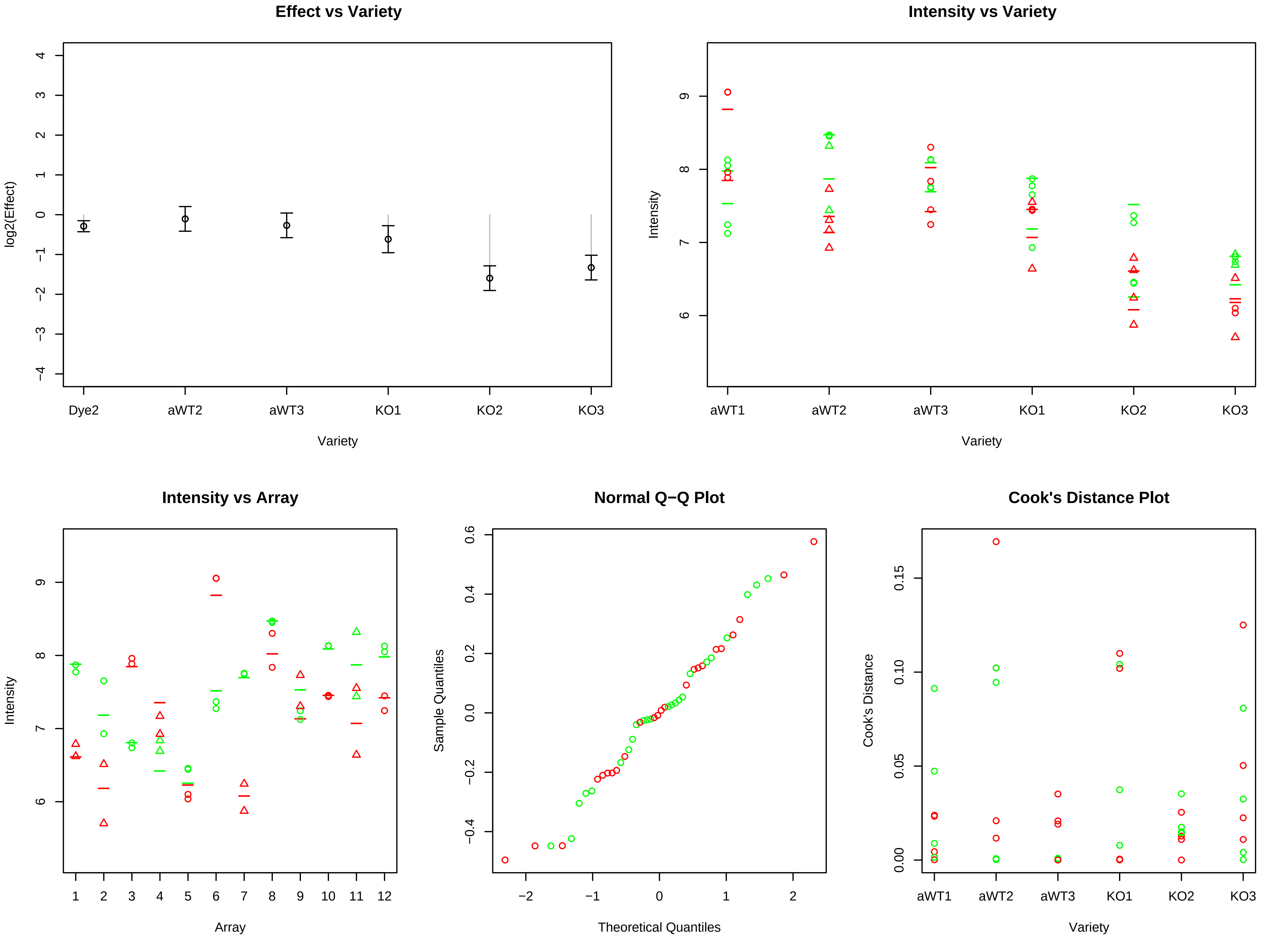

Flagged as: 111

Va = 0.01127

83 − H3118G01

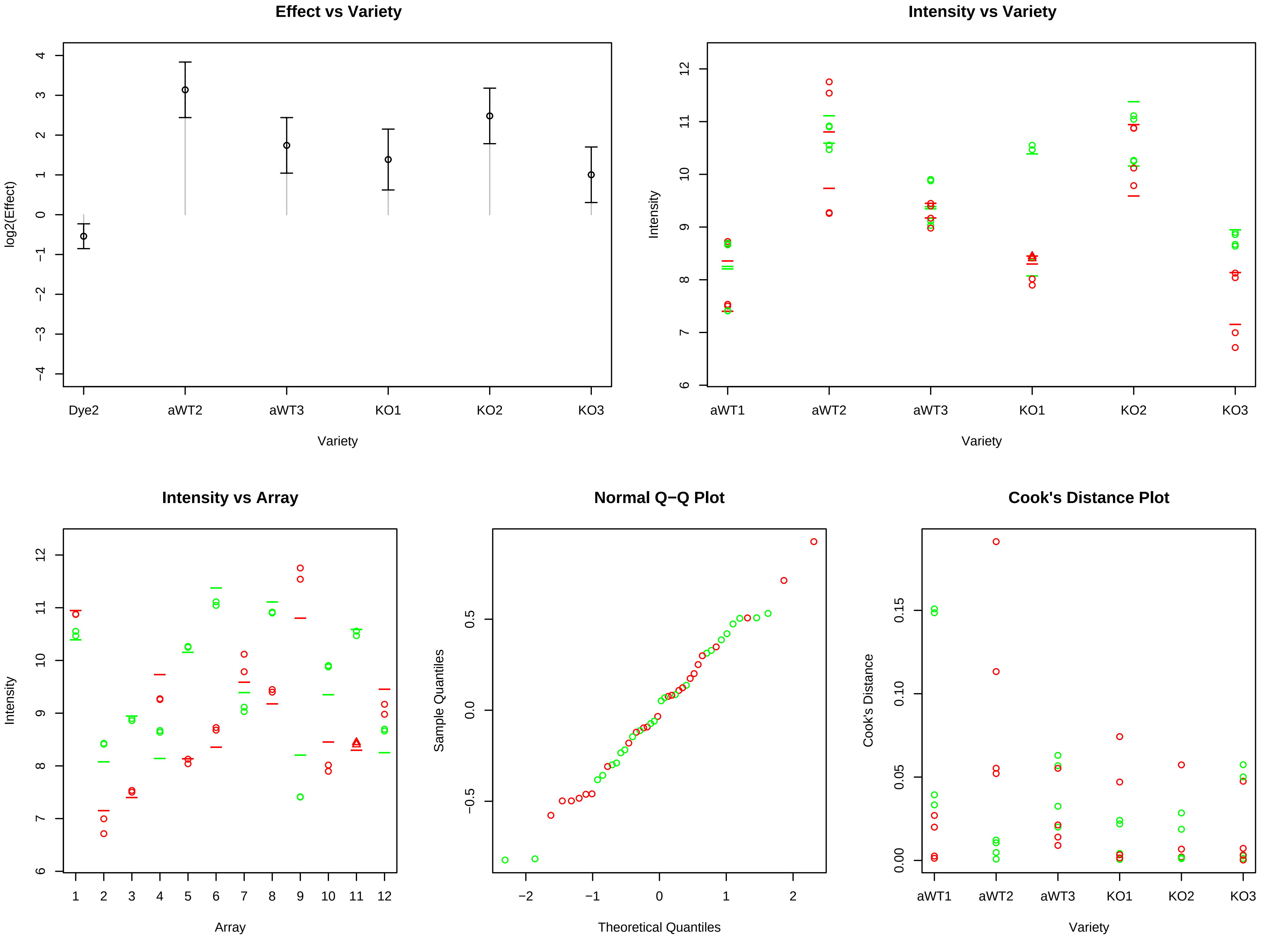

Flagged as: 111

Va = 0.02700

89 − Mus musculus RIKEN cDNA 0610007P06 gene (0610007P06Rik), mRNA

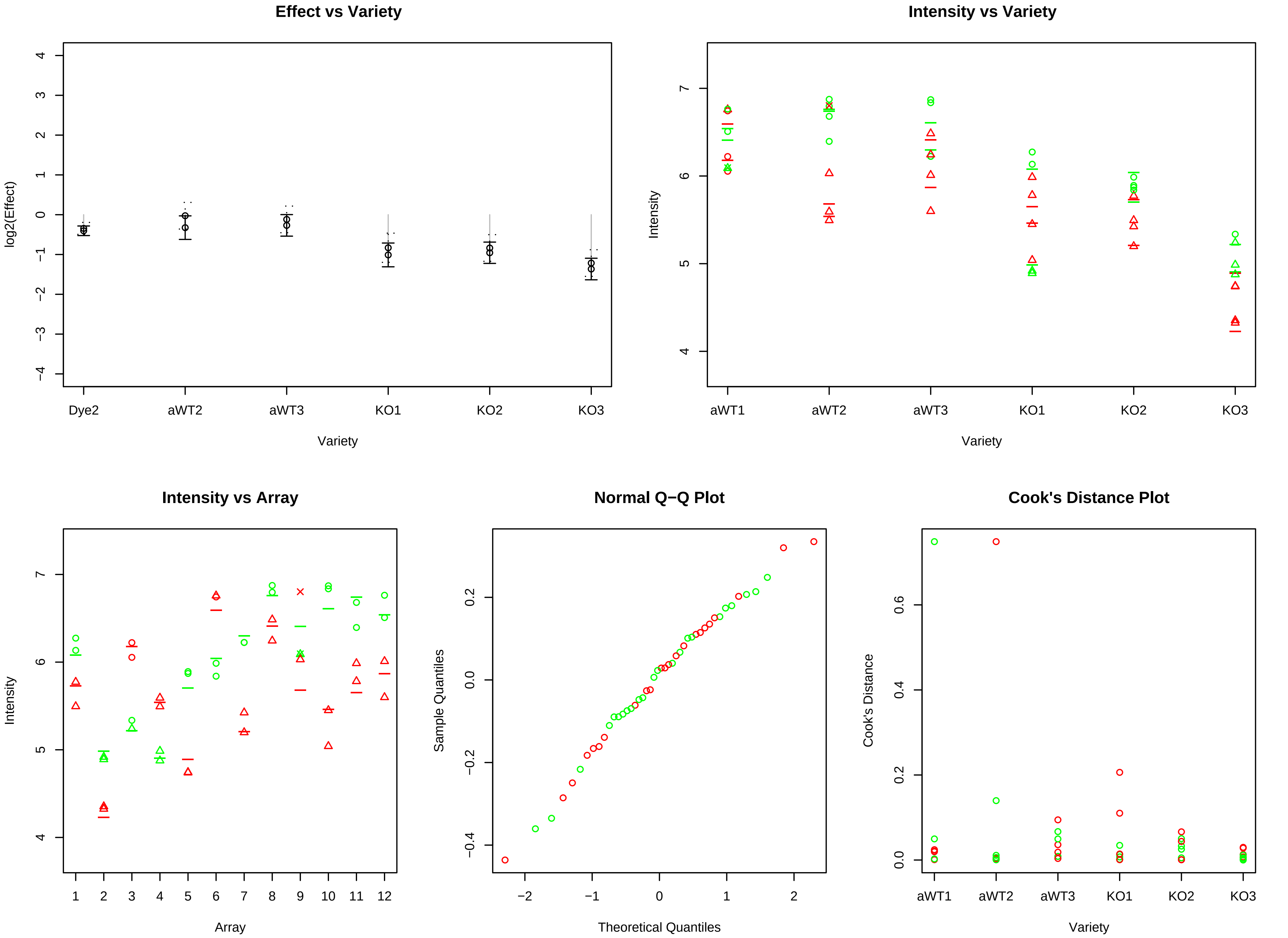

Flagged as: 111

Va = 0.01719
