# Supplementary figures and images for: CARMA: A platform for analyzing microarray datasets that incorporate replicate measures
Source: BMC Bioinformatics. 2006 Mar 17;7:149. doi: 10.1186/1471-2105-7-149 (PMC1450302; doi:10.1186/1471-2105-7-149)

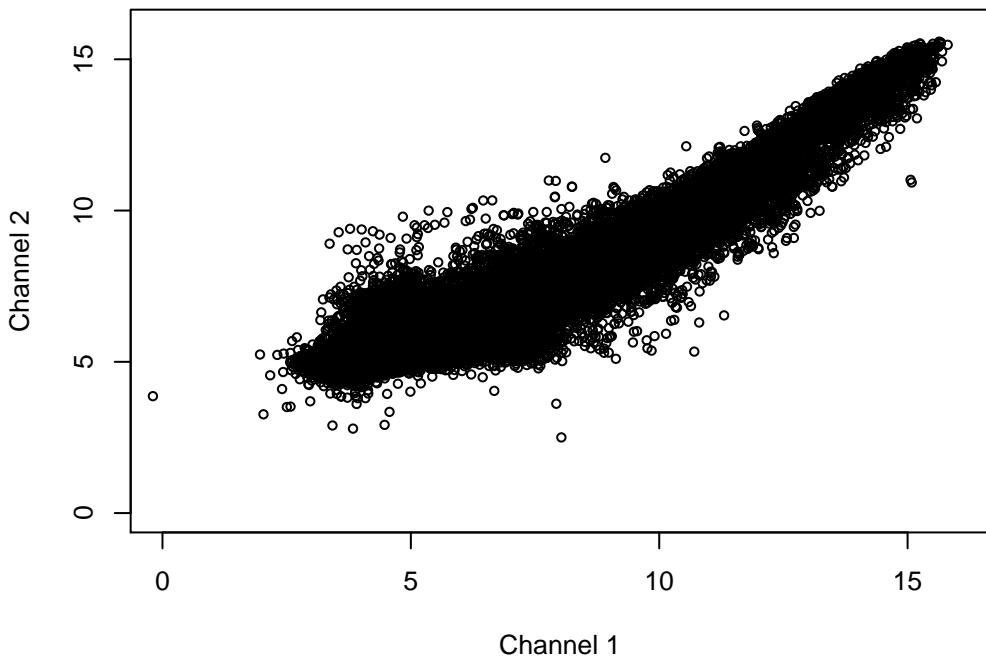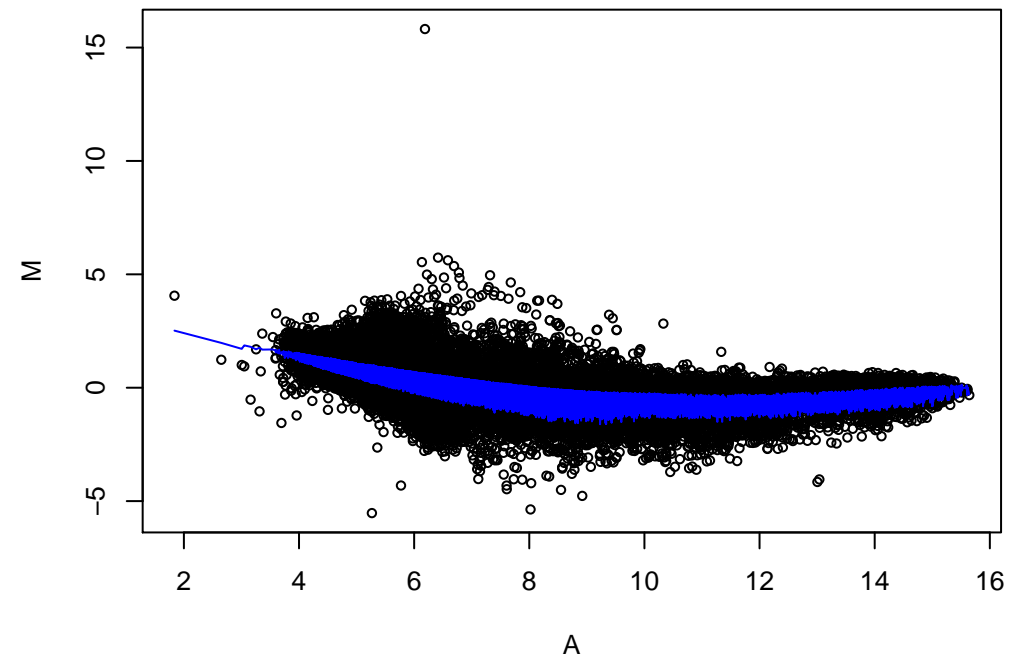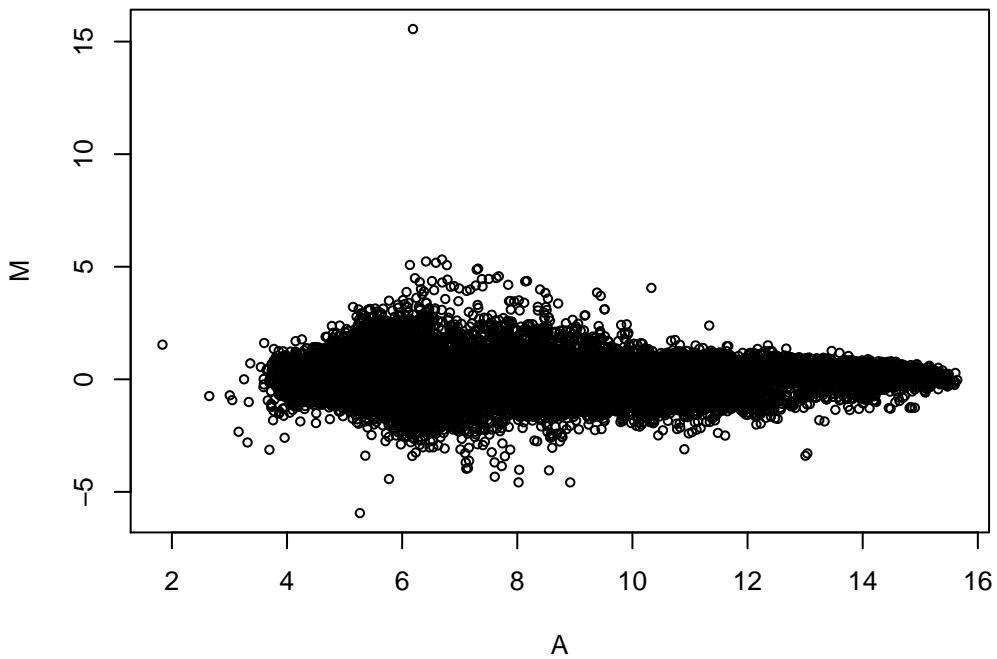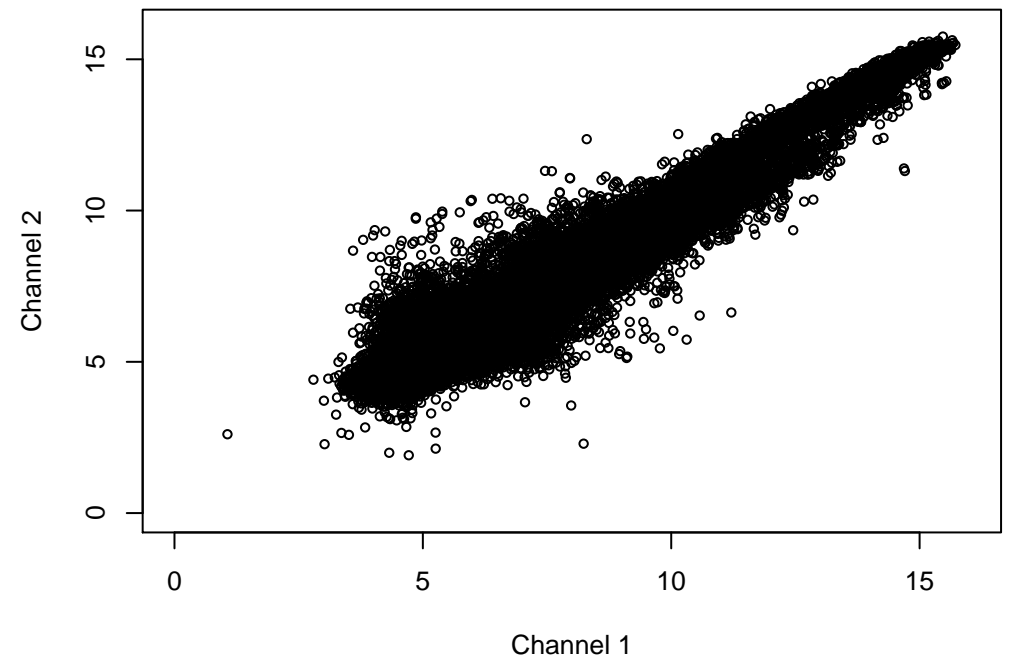

Supplement: Additional File 4 — CARMAAquaporin1.zip The configuration files used to process the aquaporin-1 example dataset using CARMA. [file 1471-2105-7-149-S4.zip › Microarray/Aquaporin1/Output/PlotNormalize.pdf]
